# Supporting Self-Management of Cardiovascular Diseases Through Remote Monitoring Technologies: Metaethnography Review of Frameworks, Models, and Theories Used in Research and Development

**DOI:** 10.2196/16157

**Published:** 2020-05-21

**Authors:** Roberto Rafael Cruz-Martínez, Jobke Wentzel, Rikke Aune Asbjørnsen, Peter Daniel Noort, Johan Magnus van Niekerk, Robbert Sanderman, Julia EWC van Gemert-Pijnen

**Affiliations:** 1 Department of Psychology, Health and Technology Faculty of Behavioural, Management and Social Sciences, Technical Medical Centre University of Twente Enschede Netherlands; 2 Saxion University of Applied Sciences Deventer Netherlands; 3 Research and Innovation Department Vestfold Hospital Trust Tønsberg Norway; 4 Embedded Information Services Library, ICT Services & Archive University of Twente Enschede Netherlands; 5 GZW-Health Psychology–GZW-General University Medical Center Groningen University of Groningen Groningen Netherlands

**Keywords:** eHealth, telemedicine, development, implementation, evaluation, multidisciplinary, qualitative evidence synthesis, meta-ethnography, systematic review, remote monitoring, self-management, cardiovascular diseases, framework, model, theory

## Abstract

**Background:**

Electronic health (eHealth) is a rapidly evolving field informed by multiple scientific disciplines. Because of this, the use of different terms and concepts to explain the same phenomena and lack of standardization in reporting interventions often leaves a gap that hinders knowledge accumulation. Interventions focused on self-management support of cardiovascular diseases through the use of remote monitoring technologies are a cross-disciplinary area potentially affected by this gap. A review of the underlying frameworks, models, and theories that have informed projects at this crossroad could advance future research and development efforts.

**Objective:**

This research aimed to identify and compare underlying approaches that have informed interventions focused on self-management support of cardiovascular diseases through the use of remote monitoring technologies. The objective was to achieve an understanding of the distinct approaches by highlighting common or conflicting principles, guidelines, and methods.

**Methods:**

The metaethnography approach was used to review and synthesize researchers’ reports on how they applied frameworks, models, and theories in their projects. Literature was systematically searched in 7 databases: Scopus, Web of Science, EMBASE, CINAHL, PsycINFO, Association for Computing Machinery Digital Library, and Cochrane Library. Included studies were thoroughly read and coded to extract data for the synthesis. Studies were mainly related by the key ingredients of the underlying approaches they applied. The key ingredients were finally translated across studies and synthesized into thematic clusters.

**Results:**

Of 1224 initial results, 17 articles were included. The articles described research and development of 10 different projects. Frameworks, models, and theories (n=43) applied by the projects were identified. Key ingredients (n=293) of the included articles were mapped to the following themes of eHealth development: (1) it is a participatory process; (2) it creates new infrastructures for improving health care, health, and well-being; (3) it is intertwined with implementation; (4) it integrates theory, evidence, and participatory approaches for persuasive design; (5) it requires continuous evaluation cycles; (6) it targets behavior change; (7) it targets technology adoption; and (8) it targets health-related outcomes.

**Conclusions:**

The findings of this review support and exemplify the numerous possibilities in the use of frameworks, models, and theories to guide research and development of eHealth. Participatory, user-centered design, and integration with empirical evidence and theoretical modeling were widely identified principles in the literature. On the contrary, less attention has been given to the integration of implementation in the development process and supporting novel eHealth-based health care infrastructures. To better integrate theory and evidence, holistic approaches can combine patient-centered studies with consolidated knowledge from expert-based approaches.

**Trial Registration:**

PROSPERO CRD42018104397; https://tinyurl.com/y8ajyajt

**International Registered Report Identifier (IRRID):**

RR2-10.2196/13334

## Introduction

### Holistic Electronic Health Research and Development

Electronic health (eHealth) is the use of technology to support health, well-being, and health care [1]. Research and development approaches in this field are derived from multiple disciplines such as computer, medical, behavioral, and design sciences [2]. Because of this, the use of different terms and concepts to explain the same phenomena [3] and the lack of standardization in reporting interventions [4] often leaves a gap that hinders accumulation of knowledge across the field.

A holistic approach—to recognize the importance of the whole and the interdependence of its parts [5]—to eHealth development could bridge this gap. In practice, this proposes that the interaction and reciprocal influence between contextual, technological, and human factors should be emphasized early and often during eHealth development and be informed by multidisciplinary perspectives [5,6].

### Frameworks, Models, and Theories for Development and Design

Researchers and practitioners of eHealth are frequently engaged in iterative phases of development that entail various activities aimed at increasing the success of an intervention (eg, exploring user preferences or assessing usability). These approaches to development are often grounded in a wide and overwhelming variety of frameworks, models, theories, guidelines, or methods [2] that extend across and within scientific disciplines. For example, a recently published framework for development of digital behavior interventions in health care outlines a process of conceptualization, development, implementation, and promotion [7]. In terms of holistic approaches, guidelines have also been used for research and development of eHealth [5,6]. Similarly, some frameworks focus on specific aspects or outcomes such as the adoption, scalability, and sustainability of health care technologies [8]. System development approaches like these are characterized by key ingredients that inform or guide eHealth research, development, implementation, or evaluation. Key ingredients can be principles, guidelines, assumptions, constructs, quality criteria, or grounding themes and ideas.

However, eHealth development is not just about the system’s development but also the decision-making process of designing intervention content or features. To this end, system development approaches are often paired with models or theories that, through their own key ingredients, describe knowledge or phenomena or propose predicted relations of constructs. Such theoretical models can be used to understand what mechanism of the intervention works best for whom, instead of evaluating the intervention as a whole (ie, as in traditional effectiveness trials). Key ingredients from theoretical models often relate to behavior change, technology adoption, or the improvement of health [9,10]. While behavior change and improvement of health are typical ingredients of face-to-face interventions, technology adoption is seen as an important outcome of eHealth implementation (because the mode of delivery is often unfamiliar to the user and continued use must be promoted). For example, the persuasive systems design model proposes means for selecting persuasive technology features (ie, promoting adoption) that work best to help users reach their own personal goals (eg, change behavior or improve health) [11]. Considering this type of knowledge, it is plausible that analyzing the use of frameworks, models, or theories applied to system development or intervention design could be a way to understand the potential of eHealth applications to complex health care issues. This focus could enable an understanding of how different approaches can interplay to increase the impact and uptake of health care technology.

### Case Study: Supporting Self-Management of Cardiovascular Diseases Through Remote Monitoring Technologies

The integration of self-management support for cardiovascular diseases (CVDs) in technology-based interventions is a case study of interest because it is a contemporary phenomenon, with boundaries not yet clearly evident, in need of answers to questions of how (ie, how does it work?) and why (ie, why does it work?) [12]. In general, CVDs constitute an alarming worldwide health care challenge [13,14] where the provision of remote self-management support is proposed to lessen their burden [15]. Because of the dynamic and continuous (daily) nature of self-management [15,16], monitoring technologies such as internet-enabled blood pressure monitors and weight scales have arguably become a prerequisite of remote care interventions. This approach can also go beyond simply monitoring by integrating mobile apps or wearables that provide coaching on self-management [17].

The potential of technology-supported remote care relies on real-time monitoring by health care teams, which could facilitate the early detection of symptom deterioration and provision of personalized and timely feedback [15] and also create a feeling of safety for patients who know they are being monitored [18]. Accumulated evidence backs up this potential, as metareviews have shown that technology-supported interventions can be at least as effective as usual care in the promotion of self-management for chronic conditions [19,20].

The complexity of CVD self-management as a health care problem and the required adaptability of the proposed technological solutions [21] naturally cause research in this area to be spread across literature from medical, behavioral, and other disciplines. For example, the expert assessment and recommended treatment of a health care provider (medical science) must be translated into motivational prompts or messages that promote adherence to a desired behavior (behavioral science) and delivered in a salient and understandable way to the patient (human-technology interaction science). Thus, the multidisciplinary gap that characterizes eHealth could also be present in the case of CVD applications.

By and large, it can be recognized that much is generally known about eHealth development processes, based on the many examples that exist, and that research in self-management and CVD is at least equally extensive. Frameworks, models, and theories can be valuable sources of knowledge but are numerous and difficult to compare. Therefore, what is lacking at this point is an analysis that relates the knowledge across different disciplines, the variety of development approaches, and their operationalization in specific settings such as eHealth applications to self-management of CVD.

### Aim and Focus

The aim of this review was to identify and compare the underlying approaches that have contributed to the research and development of interventions focused on self-management support of CVDs through the use of remote monitoring technologies. The focus was on frameworks, models, and theories and the reported operationalization of their key ingredients in published studies. The review aimed to create a synthesis that highlighted common (or conflicting) principles, guidelines, and methods to create an understanding of the distinct approaches that have been used for the case at hand.

The review seeks to answer the following research questions. First, what frameworks, models, or theories have been used to develop, implement, or evaluate interventions to support self-management of patients with CVD through the use of remote monitoring technologies? Second, what are the key ingredients of these frameworks, models, or theories that inform or guide the system’s (1) development, implementation, or evaluation; (2) content design to promote behavior change and technology adoption; and (3) proposed effects in terms of health-related outcomes? Third, to what extent do the key ingredients of these frameworks, models, or theories fit with the principles of the holistic research and development approach of eHealth [5,6]?

### Selecting Metaethnography

The review was based on metaethnography, a qualitative evidence synthesis method originally developed by Noblit and Hare [22]. Metaethnography is an interpretive approach that seeks to generate a new understanding of a topic. In practice, it involves open coding and constant comparison of key metaphors, which can be phrases, ideas, concepts, perspectives, organizers, or themes identified across the reviewed studies [22]. Metaethnography was selected by following guidelines on choosing qualitative evidence synthesis methods [23]. Mainly, metaethnography was preferred because it aims for an interpretive examination of themes, relationships, and contradictions in the literature, while preserving the social and theoretical contexts of the original findings [24-27]. Additionally, metaethnography fits perfectly with the study aims because it explicitly compares and analyzes texts with the intention to build a holistic interpretation [22]. The aims and methods of the review were described in more detail in a protocol that was preregistered [CRD42018104397] and published [28], complying with best-practice recommendations [24].

## Methods

### Review Phases

**Figure 1:**
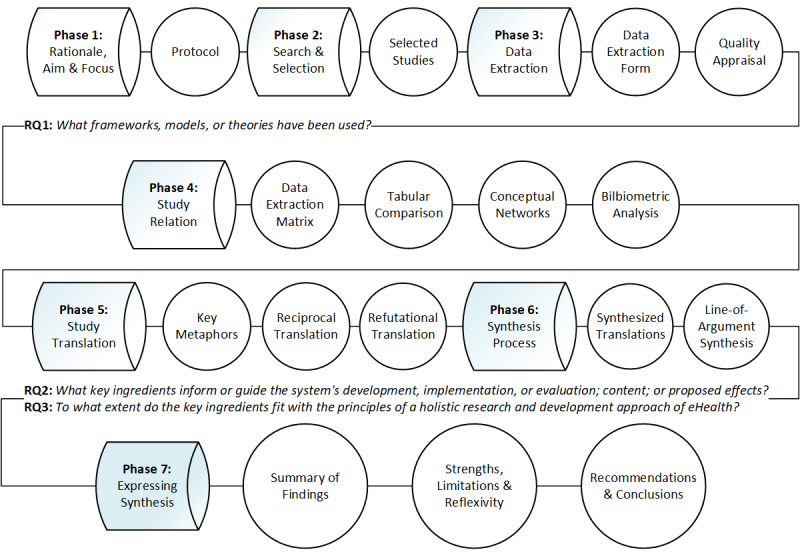
Phases and key output of the metaethnography review. RQ: research question.

Figure 1 visualizes the phases and key output of this review in relation to the research questions. Phase 1 generated the published protocol [28]. Phases 2 and 3 sought to answer the first research question regarding the identification of frameworks, models, and theories [28]. Phases 4 to 6 operationalized an answer to the second and third research questions through an interpretive characterization of the key ingredients of frameworks, models, and theories [28]. This paper adheres to the recently developed Meta-Ethnography Reporting Guidance (eMERGe) [27].

### Search Strategy

A comprehensive search was conducted to find published studies (articles) of interest [28]. The search consisted of a systematic database search. Seven databases were used: Scopus, Web of Science, EMBASE, CINAHL, PsycINFO, Association for Computing Machinery Digital Library, and Cochrane Library. The databases were chosen based on their coverage of various fields of science related to eHealth. Multiple key terms were used as part of a highly structured query consisting of four sets aiming for results about frameworks, models, and theories (set 1), eHealth interventions (set 2), self-management (set 3), and cardiovascular diseases (set 4) [28]. The database search was conducted by the main reviewer (RRCM). The search results were uploaded to EndNote X8 (Clarivate Analytics), and duplicates were eliminated.

### Study Selection

The selection was performed using the Covidence Web-based software platform (Veritas Health Innovation Ltd). Records were screened by two reviewers, first by title and abstract, and then by full text. The main reviewer conducted the screening throughout all stages. A co-reviewer (RAA) screened at least 15% of the records at each stage and also those tagged as maybe in Covidence by the main reviewer. While 100% dual review would be ideal [29], the approach taken strived for a balance between thoroughness and speed, which is a common strategy for systematic reviews [30] (for full details about study selection see the protocol [28]). Discrepancies between reviewers were discussed and resolved at every stage. Multimedia Appendix 1 presents the selection criteria.

### Reading Studies and Extracting Data

The included articles were uploaded to the qualitative software package ATLAS.ti version 8 (ATLAS.ti Scientific Software Development GmbH). Their content was coded according to the elements of a highly detailed data extraction form created to fit the scope of this review (see Multimedia Appendix 1). The data extraction form was based on the Consolidated Standards of Reporting Trials of Electronic and Mobile Health Applications and Online Telehealth (CONSORT-EHEALTH) checklist v.1.6 [31,32]. The form was piloted and iteratively refined throughout all phases. All coded data were also translated to a single form per study. The main reviewer iteratively read, coded, and updated all of the data extraction output based on input from three co-reviewers (RAA, JW, and JGP). The main reviewer also appraised the quality of studies using items from the Critical Appraisal Skills Program’s checklists. These checklists were employed because they are a suggested and frequently used tool for metaethnographies [25,26,33-38]. Quality appraisal is not a strict requirement for metaethnography because the richness and relevance of the content is more important [36], but it is considered good practice and was used to get further familiarized with the studies [26].

### Determining How Studies Are Related

The relation of studies was performed at three levels with the aim to provide a deep analysis of the data and its context. At the first level, the frameworks, models, theories, and their key ingredients were compared in tabular form, along with their definitions and occurrence in studies. Additionally, conceptual networks were created in ATLAS.ti using key metaphors as nodes to visualize and explore potential relations (see example in Multimedia Appendix 2).

At the second level, the characteristics of included studies and their overarching projects were compared in tabular form. Moreover, the general context of the underlying approaches was compared. To do this, a matrix was created to visualize the perceived level of clarity and extent of the extracted data [28].

The third level focused on the sources of the underlying approaches identified or cited in included studies (eg, the cited reference of a framework applied in a study). This approach used an explorative bibliometric analysis to assess the multidisciplinary range of such literature and investigate any potential relationships or trends. The references were snowballed and accompanied with co-citation analysis and topic modeling [39-41]. Snowballing can be an alternative to traditional systematic review methods used to identify literature pertaining to a topic of interest by scanning reference lists of studies [42].

### Translating Studies

Key metaphors were systematically translated across studies in order to arrive at concepts that embodied more than one study [43]. The translation in a metaethnography is idiomatic (translating the meaning of the text) rather than literal (word for word), and it must take into account the context of the study [22,43]. This stage is characterized by two types of translation: reciprocal and refutational. Reciprocal translations aim to identify or generate metaphors that can better enable holistic accounts of phenomena [22]. On the other hand, refutational translations aim to give explicit attention to incongruities and inconsistencies in the data [43].

To avoid missing valuable insights, the review collected two types of metaphors distinguished by their source. Primary key metaphors were the key ingredients of frameworks, models, or theories operationalized by the authors of a study. Secondary key metaphors were remarkable phrases, concepts, ideas or perspectives by the authors of a study but not apparently derived from a structured underlying approach. Moreover, to assist the translation process it was decided to use the principles of the Center for eHealth Research (CeHRes) roadmap [5,6]. The roadmap is a guideline for holistic eHealth development that is itself based on a review of multiple frameworks and grounded in the integration of persuasive technology design [11], human-centered design, and business modeling [6]. It proposes five principles: (1) eHealth development is a participatory development process; (2) eHealth development creates new infrastructures for improving health care, health, and well-being; (3) eHealth development is intertwined with implementation; (4) eHealth development is coupled with persuasive design; and (5) eHealth development requires continuous evaluation cycles. The roadmap was only used to confront what has been done in the literature with an initial assumption of principles for a holistic approach. This step was operationalized by collectively characterizing both types of metaphors under one or several principles. This process created five clusters, each representing a principle. In the same manner, metaphors were also characterized to clusters of behavior change, technology adoption, or health-related outcomes if they were identified as potential enablers of intervention effectiveness. Clustering the metaphors allowed reviewers to deal with a smaller amount of metaphors at a time so idiomatic translations were performed under each cluster.

### Synthesis Process

The relation and translation of studies as well as the synthesis process are considered complex analytical and highly overlapping phases of a metaethnography without a one-size-fits-all recipe [43]. To clarify the resulting approach of this review, Multimedia Appendix 2 includes an overview of the activities undertaken in phases 4 to 6 (see Figure 1). From the interpretive findings of the previous phases, a textual line-of-argument synthesis was created. A line-of-argument synthesis is a new storyline or overarching explanation of a phenomenon [43] (the third type of relation after reciprocal and refutational analysis). The synthesis was structured by revising the assumed holistic principles and emphasizing the key metaphors that extended over several themes. Each key metaphor could either contribute to the understanding of specific approaches, highlight important relations across multidisciplinary literature, or even suggest new knowledge derived from integrating unrelated approaches.

## Results

### Selected Studies

**Figure 2:**
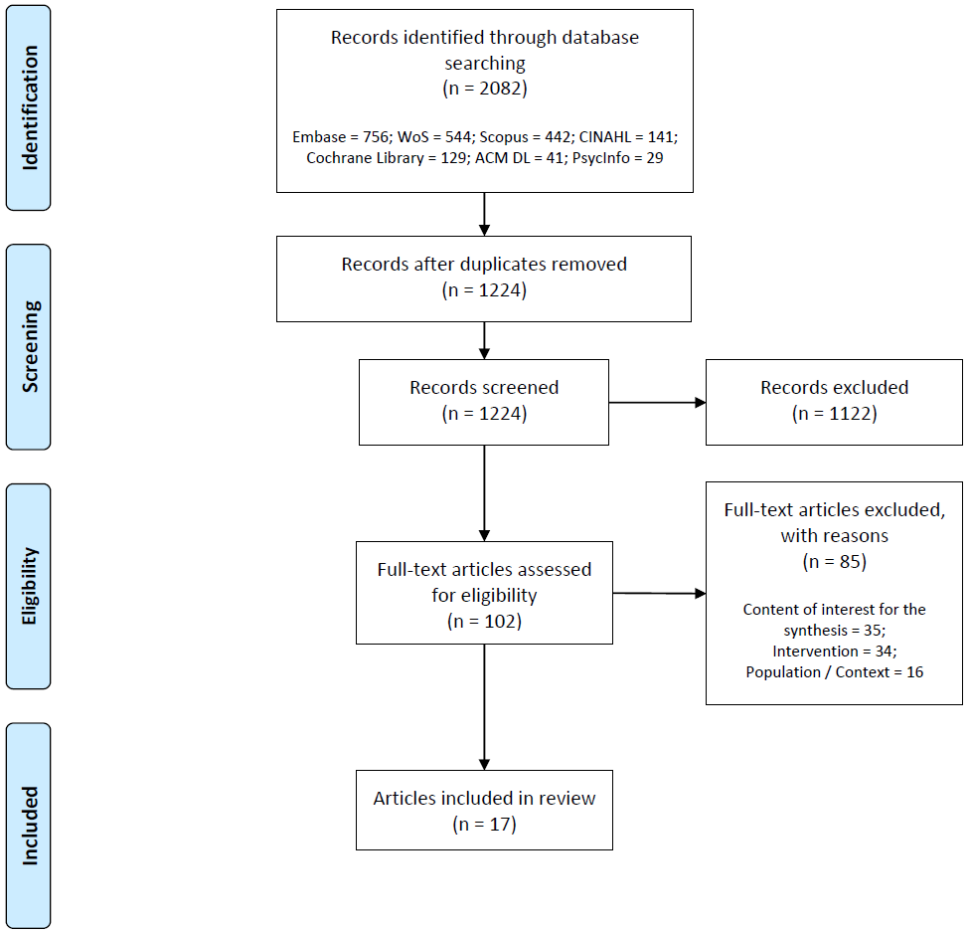
Flowchart of included and excluded articles.

The initial search resulted in 1224 eligible records after removing duplicates. During the title and abstract screening, 1122 records were excluded; 85 more articles were excluded during full-text screening. Of articles excluded by full-text, 41% (35/85) had no relevant content for the synthesis (eg, no apparent framework applied), 40% (34/85) described an intervention that was out of scope (eg, not focused on self-management), and 19% (16/85) had an irrelevant target population or context (eg, not focused on CVD). Multimedia Appendix 3 lists all articles excluded by full-text. In the end, 17 articles were included for the review. Figure 2 summarizes the selection process via the Preferred Reporting Items for Systematic Review and Meta-Analyses (PRISMA) flowchart [44]. The keywords, search string, and any additional settings used for the database search are reported as preliminary results in the protocol of this review [28].

### Study Characteristics

An overview of the characteristics of included articles can be found in Table 1 (see full table in Multimedia Appendix 4). The articles address 10 different overarching projects, identified as HeartMapp [45-47], Home and Online Management and Evaluation of Blood Pressure (HOME BP) [48-50], Seamless User-Centered Proactive Provision of Risk-Stratified Treatment for Heart Failure (SUPPORT HF) [51-53], PATHway [54,55], Congestive Heart Failure—Personalized Self-Management System (CHF PSMS) [56], MedFit [57], Smartphone Medication Adherence Stops Hypertension (SMASH) [58], Engage [59], MyHeart [60], and a mock-up [61] standalone study. The year of publication of the articles ranged from 2009 to 2018. The United Kingdom (n=7) and United States (n=5) were the most common affiliations of the authors. The most frequent journals in which the articles were published are the Journal of Medical Internet Research (3/17), BMC Medical Informatics and Decision Making (2/17), and Applied Nursing Research (2/17). The articles were also divided across three target conditions: heart failure (9/17), hypertension (4/17), and CVDs in general (4/17).

Study design classification was done according to the Oxford Centre for Evidence-Based Medicine [62]. Analytic experimental studies are those in which the researcher manipulates the exposure, allocating subjects to the intervention or exposure group. Analytic observational studies are those in which the researcher simply measures the exposure or treatments of the groups without manipulating the exposure or allocation of subjects. Descriptive (qualitative) studies do not try to quantify the relationship but try to give a picture of what is happening in a population.

A total of 35% (6/17) of articles [46,48,49,52,54,60] focused on describing the development process of an intervention and often generally discussed results from more than one study. In these cases, classification by study design was not applicable. For the remaining articles, study design classification revealed three types of study designs used for eHealth research: analytic observational [45,51,56,59,61], descriptive qualitative [47,50,53,55,57], and analytic experimental [58]. Multimedia Appendix 5 presents the quality appraisal of selected studies.

**Table 1:** Characteristics of included articles.

Project	Author; country^a^; year	Journal	Target
MyHeart	Villalba et al [60]; Spain; 2009	Conference publication; International Conference on eHealth, Telemedicine, and Social Medicine	HF^b^
SMASH^c^	McGillicuddy et al [58]; US; 2012	Conference publication; Wireless Health	HTN^d^
CHF PSMS^e^	Bartlett et al [56]; UK; 2014	BMC Medical Informatics and Decision Making	HF
SUPPORT HF^f^	Rahimi et al [51]; UK; 2015	European Heart Journal—Quality of Care and Clinical Outcomes	HF
SUPPORT HF	Triantafyllidis et al [52]; UK; 2015	International Journal of Medical Informatics	HF
SUPPORT HF	Chantler et al [53]; UK; 2016	Digital Health	HF
HOME BP^g^	Band et al [48]; UK; 2016	BMJ Open	HTN
HOME BP	Band et al [49]; UK; 2017	Implementation Science	HTN
HOME BP	Bradbury et al [50]; UK; 2017	BMC Medical Informatics and Decision Making	HTN
HeartMapp	Athilingam et al [45]; US; 2016	Applied Nursing Research	HF
HeartMapp	Athilingam et al [46]; US; 2018a	CIN: Computers, Informatics, Nursing	HF
HeartMapp	Athilingam et al [47]; US; 2018b	Applied Nursing Research	HF
Engage	Srinivas et al [59]; US; 2017	International Journal of Human—Computer Interaction	HF
MedFit app	Duff et al [57]; Ireland; 2018	JMIR Formative Research	CVDs^h^
no project	Baek et al [61]; South Korea; 2018	JMIR Cardio	CVDs
PATHway	Walsh et al [54]; Ireland, Belgium, Italy, Greece; 2018a	Translational Behavioral Medicine	CVDs
PATHway	Walsh et al [55]; Ireland, Belgium; 2018b	Journal of Medical Internet Research	CVDs

^a^Countries are included according to the reported affiliations of the authors.

^b^HF: heart failure.

^c^SMASH: Smartphone Medication Adherence Stops Hypertension.

^d^HTN: hypertension.

^e^CHF PSMS: Congestive Heart Failure—Personalized Self-Management System.

^f^SUPPORT HF: Seamless User-Centered Proactive Provision of Risk-Stratified Treatment for Heart Failure.

^g^HOME BP: Home and Online Management and Evaluation of Blood Pressure.

^h^CVD: cardiovascular diseases (in general).

### Frameworks, Models, and Theories Applied to Research and Development

In total, 43 frameworks, models, or theories were identified as underlying approaches of the included studies. Textbox 1 and 2 present all of the identified approaches. Multimedia Appendix 6 includes the list of all key ingredients with their definitions per study. Multimedia Appendix 7 and 8 present the full relation between each underlying approach and the operationalized key ingredients by the included studies. In total, 27 different approaches were used to inform the system’s development, implementation, or evaluation [52,58,63-92] (Textbox 1 and Multimedia Appendix 7). In contrast, 16 theoretical models were used to inform the system’s content [50,93-113] (Textbox 2 and Multimedia Appendix 8).

Multimedia Appendix 7 shows that approaches to system development undertaken by the included studies often promote a participatory, user-centered approach—for example, the development and evaluation process for mHealth [71] or the person-based approach [81,82]. Several types of user-centered models were also identified [61,87,88,90]. Similarly, some frameworks were used to broaden the designer’s perspective. For example, the systems engineering initiative for patient safety 2.0 [85] and the patient work lens for consumer-facing health [80] “encouraged the design team to ‘think systems’ and ‘think bigger,’ which in this case meant consideration of patients’ long-term goals, overall workload, and integration of self-care recommendations into daily life” [59]. Among some focused approaches were, for example, the business-oriented frameworks applied in the HeartMapp project [47].

**Frameworks and models that informed the system’s development, implementation, or evaluation.**

- 5E usability approach [63]
- Action research [64,65]
- Agile software development [66]
- Behavior change wheel/capability, opportunity, motivation, and behavior model [68,69]
- Business model canvas
- Business research method [70]
- Development and evaluation process for mHealth [71]
- Goal-directed design [72]
- Holistic patient interaction model [73]
- Intervention mapping [74]
- Iterative design model [58]
- Iterative refinement and patient participatory approach [52]
- Iterative software design process [73]
- Iterative software development [75]
- Medical Research Council’s guidance for developing and evaluating complex interventions [76-78]
- Multiphase optimization strategy [79]
- Patient work lens for consumer-facing health [80]
- Person-based approach [81,82]
- Practical guide to usability testing [67]
- Realistic evaluation framework [83,84]
- Startup owner’s manual [86]
- Systems engineering initiative for patient safety 2.0 [85]
- User-centered design (ad hoc) [87,88]
- User-centered design [89]
- User-centered design of consumer-facing health information technology [90]
- User-centered design (International Organization for Standardization 1999) [91]
- Usability framework [92]

**Theoretical models that informed the system’s content.**

- Cognitive load theory [93]
- Cognitive theory of multimedia learning [94]
- Common sense model of self-regulation [95]
- Congratulate, ask, reassure, encourage approach [50]
- Control theory framework for personality—social, clinical, and health psychology [96]
- Domestication of technology theory [97,98]
- Information, motivation, behavioral skills model [99]
- Instructional design approach using a pedagogical agent [100]
- Multidimensional framework for patient and family engagement in health and health [113]
- Normalization process theory [101-103]
- Problem-based learning [104]
- Self-determination theory [105,106]
- Social cognitive theory [107-109]
- Social ecological model
- Technology acceptance model [110]
- Unified theory of acceptance and use of technology model [111,112]

Multimedia Appendix 8 shows the wide variety of theoretical models that were used to inform the system’s content in the included studies. For instance, social cognitive theory [107-109] was used to outline the behavioral perspective of three different projects. Additionally, some theories were used to understand the process of technology adoption as an outcome, such as the domestication of technology theory [97,98] or the normalization process theory [101-103]. Comparably, technology acceptance was also analyzed through the unified theory of acceptance and use of technology model [111,112] and the technology acceptance model [110].

In general, the integration of multidisciplinary frameworks was frequent in the included studies and their overarching projects. Multimedia Appendix 9 presents how the overarching projects of included studies and their underlying approaches were compared across several levels. Multimedia Appendix 7 and 8 make evident that the HeartMapp [45-47], HOME BP [48-50], SUPPORT HF [51-53], PATHway [54,55], CHF PSMS [56], MedFit [57], SMASH [58], and MyHeart [60] projects were all informed by a combination of approaches from different areas of science. In contrast, the Engage [59] project focused on macroergonomic sociotechnical frameworks while the mock-up [61] study concentrated on a user-centered design research process. However, comparability across projects was influenced by the clarity and extent of the reported data in the selected articles. Multimedia Appendix 10 exemplifies the differences in clarity across studies and projects, while the full comparative analysis can be observed in Multimedia Appendix 9.

The multidisciplinary-based approach was sometimes an explicit goal of researchers. For example, the MedFit study aimed to adopt a “multidisciplinary approach to development [...] drawing on theories from engineering, computer science, and health psychology” [57]. In this line, frameworks were sometimes used to inspire tailored approaches. The most remarkable case was the guidance from the Medical Research Council (MRC) for developing and evaluating complex interventions [76-78], which informed four projects and in general was cited repeatedly in the included studies (see also its relative importance identified in the bibliometric analysis in Multimedia Appendix 9). However, sometimes how a framework informed another one was not completely clear. For example, the iterative software design approach of the MyHeart project [60] was stated to be informed by goal-directed design [72] and user-centered design [91] principles, but this statement was not elaborated in the selected article. Another example is the iterative refinement and patient participatory approach of the SUPPORT HF project [52], which is informed by action research [64,65] and agile software development [66] frameworks. Although such approach is clearly described, its explicit relation to the underpinning frameworks is not explicitly established.

### Key Ingredients That Inform or Guide Development, Content, or Outcomes

The key ingredients as presented in Multimedia Appendix 7 and 8 facilitate a more detailed comparison of how underlying approaches were used. Mainly, the approaches to system development contain key ingredients that mostly represent stages of development, implementation, or evaluation. The stage-based ingredients that focus on creating a fit between the user and the proposed solution (eg, through co-design and formative evaluation) are eHealth-specific frameworks [71,81,82], guidelines for (software) iterative evaluation [52,58,73], or user-centered design methods [87-91]. On the other hand, some stage-based key ingredients guided systematic exploration, selection, and integration of theory with empirical evidence (eg, establishing why or how the intervention works through theoretical modeling). These ingredients are instead derived from research and intervention-building frameworks from behavioral [74], medical [76-78], or sociological [83,84] areas of science. Other key ingredients did not represent stages of development but were constructs used to broaden the designers’ perspectives—for instance, to understand human-technology interaction [73], the patient’s work system [80,85] (ie, the workflow), ecosystem levels in health care [55], or key insights for business modeling [47]. The bibliometric analysis on the cited references of underlying approaches also observed a distinction between topics of intervention development, behavior change, and health care (see Multimedia Appendix 9).

In contrast, theoretical models provide key ingredients that were used to inform the content or outcomes of interventions. These ingredients could be psychological determinants [68,69,95,99,105-109] (eg, self-efficacy), mechanisms of action (self-monitoring [96]), or mediators (engagement [113]) for behavior change. Other key ingredients are about eHealth adoption, such as determinants of technology acceptance [110-112] (eg, ease of use) or processes and mediators of adoption [97,98,101-103] (eg, objectification).

In sum, the included studies highlighted participatory, user-centered, and iterative approaches with multiple perspectives about how to effectively influence the uptake of eHealth at several levels (eg, from individual cognition to the elements of a macroergonomic work system). Multimedia Appendix 6 shows how these ingredients and other insights (metaphors) of the included studies were compared and translated within and across studies. In the sections below, the included studies [45-61] (Multimedia Appendix 4) are mentioned by the first author’s name in the text, their underlying approaches (Textbox 1 and 2 and Multimedia Appendix 7 and 8) are named and referenced when applicable.

### Behavior Change

The effectiveness of eHealth systems in the included studies in terms of behavior change was operationalized by their success in improving self-management behaviors. In this regard, the operationalization of key ingredients could be better understood through the sociotechnical perspective which broadly conceptualizes self-management as a complex biopsychosocial process, as proposed by the systems engineering initiative for patient safety 2.0 [85] and the patient work lens for consumer–facing health [80] model. Throughout the included studies, the proposed general solution was the provision of tailored, personalized, or timely support (Band et al [48]), grounded in the potential of eHealth to deliver behavior change techniques that can facilitate long-term sustained behavior change (Duff et al [57]). Key ingredients were mostly informed by psychological theories such as social cognitive theory, which highlights determinants like self-efficacy, outcome expectancy, individual goals, and perceived impediments and facilitators [107-109]. Likewise, information, motivation, behavioral skills, and social opportunity were also parameters used by the selected studies to facilitate behavior change, based on the behavior change wheel [68,69] or the information, motivation, behavioral skills model [99]. Behavior change was also proposed to be at play during the adoption of a technology according to the normalization process theory [101-103]—for example, to explain how patients or health care providers must integrate several behaviors into everyday life (interactional workability) or how patients must be able to adapt their self-care routines when required (reconfiguration). Finally, the review collected a long list of practical applications (translations of behavior change techniques into intervention components) that showcased the similarity of current approaches to support self-management through remote monitoring technologies. For example, a familiarization phase (Walsh et al [54]) with the technology was an approach used by several studies. The most common features of the technologies included assessment, self-monitoring, feedback (during activity and after performance), behavioral change support (eg, goal setting, promoting home exercising), and education (eg, on disease management).

### Technology Adoption

The effectiveness of eHealth systems in the included studies in terms of promoting technology adoption during the implementation process was operationalized mainly by the aim to create a fit between the system and the self-management routines of the patients. Primarily, technology adoption was informed in the included studies by the recognition of a diversity of user experiences (Chantler et al [53]), and the predominant strategy to undertake user-centered design [87,89] to address this heterogeneity. Once again, the tailored, personalized, and timely support (Band et al [48]) was the main driver during operationalization. Specifically, the adaptation to the personal routines of patients (Villalba et al [60]) was identified as a common idea across the included literature. In addition, the inclusion of a bidirectional service model (as in Baek et al [61]) or blended care which entailed communication between health care providers and patients was also an important theme across the included studies. This was in part because the sense of connection to a support team that a system provides to a patient could act as a key motivator for the use of the technology (Chantler et al [53]). Guidelines for health care providers to offer patient-centered support within a remote care context were applied by one of the included studies (Bradbury et al [50]). Remarkably, two major challenges of technology adoption were also identified. First, the technology knowledge gap (literacy) between younger and older generations (discussed by Duff et al [57]). Second, the inertia of disengagement, which was proposed to be tackled by the establishment of design goals that promote rather than assume baseline levels of engagement (Srinivas et al [59]). Technology adoption could also be assessed at multiple levels—for example, through a user interaction model (applied by Villalba et al [60]) that investigates the explicit and implicit interaction between the user and the technology or in terms of a multidimensional usability framework (applied by Walsh et al [54]). Notably, technology adoption could be explored through models such as the domestication of technology theory [97,98], which describes the processes of acceptance, rejection, and use of technology by its users (applied in Chantler et al [53]). Likewise, the unified theory of acceptance and use of technology and the technology acceptance model were other models of adoption that offered determinants such as behavioral intention, performance and effort expectancy, experience, and price value [110-112]. Finally, the key insights for building a minimum viable product (eg, value propositions), derived from the business model canvas, were also interpreted as key ingredients to enable the desired adoption during implementation of the technology (Athilingam et al [47]).

### Health-Related Outcomes

The paths to health improvement of the eHealth systems in the included studies were several. Overall, most of the listed ingredients could be categorized as engagement outcomes (eg, continued use and high usability), behavioral outcomes (eg, improved self-management), or health-related outcomes (eg, reducing admissions or increasing quality of life). In these terms, the operationalization of health-related outcomes in the selected studies focused notably on behavior change as the indirect path to increase health, an approach often grounded in the behavior change wheel and its capability, opportunity, motivation and behavior model [68,69]. For example, technologies were designed to include several intervention functions, such as enablement (increasing means and reducing barriers to perform the behavior), education (increasing knowledge or understanding), and environmental restructuring (changing the physical or social context). Moreover, the sociotechnical perspective of the systems engineering initiative for patient safety 2.0 [85] was used by Srinivas et al [59] to broaden the understanding of the various components of an intervention (eg, work processes) in relation to their impact on potential outcomes (proximal or distal, desirable or undesirable). An important challenge to improve health in the selected literature focused on hypertension (see Table 1 and Multimedia Appendix 4) was clinical inertia [58] (ie, the failure to establish appropriate targets and escalate treatment to achieve treatment goals). Additionally, the accurate measurement of changes in key determinants (eg, knowledge, as approached by Bartlett et al [56]) was also a possible methodological obstacle.

### Fit of Key Ingredients With Holistic Principles for Research and Development

Projects at the intersection of self-management, CVD, and eHealth have directly or indirectly applied holistic principles for research and development. Namely, the principle of eHealth as a participatory development process and the principle that eHealth development is intertwined with implementation have been explicitly endorsed in the included studies. On the other hand, the principle that eHealth development creates new infrastructures for improving health care, health, and well-being has been partially operationalized through the use of various frameworks but has remained unacknowledged as a key underlying theme. Similarly, the principle that eHealth requires continuous evaluation cycles has been indirectly addressed by a wide variety of aims and methods operationalized across many phases of the eHealth development process. Ultimately, the principle that eHealth development is coupled with persuasive design was unacknowledged across included studies, although varied and generic approaches to inform design were found. Multimedia Appendix 11 illustrates the terms and definitions (metaphors) that constituted each cluster through conceptual networks.

### Development Is a Participatory Process

The principle of participatory development has been widely operationalized as part of a fundamental integration of person-based approaches with theory and evidence (Band et al [49]) and directly grounded in the concept of user involvement, which was promoted throughout the included literature to conform with the guidelines of the MRC [76-78]. Moreover, the participatory approach was complemented with a socioecological perspective to secure inclusion of a diversity of user experiences (Chantler et al [53]) and multiple levels of the target group’s ecosystem (as applied by Walsh et al [55]). The aims and methods for participatory development of the included studies have been extensively underpinned by user-centered design [87,89] and applied to the full extent of eHealth development phases (from planning to deployment [80-82,88]).

### Development Creates New Infrastructures for Improving Health Care, Health, and Well-Being

Initially, the principle that eHealth development creates new infrastructures for improving health care, health, and well-being was thought to be self-evident given the scope of the review (remote care). The established aims of researchers and developers in the selected studies endorsed this principle, such as providing tailored, personalized, and timely support (Band et al [48]) or the unobtrusive remote delivery of system refinements (Triantafyllidis et al [52]). Key contextual factors were also highlighted by the included studies, such as the facilitating conditions (perceptions of the resources and support available to perform a behavior) defined by the unified theory of acceptance and use of technology model [111,112]. In this regard, as posed by the behavior change wheel [68,69], context can also include the policy categories surrounding technology-supported interventions (decisions made by authorities that help to support and enact an intervention). An early step to create an infrastructure can be to develop a program plan to describe the scope and sequence of intervention components, its required materials, and the protocols for implementation (as in intervention mapping [74]). In addition, the use of interdisciplinary methods (eg, factorial or fractionated evaluation designs discussed by Walsh et al [54]) and a socioecological perspective (Walsh et al [55]) are approaches that can facilitate the understanding of eHealth infrastructures and ecosystems (ie, identifying what works, who should be involved, and how in remote care support).

### Development Is Intertwined With Implementation

An implementation focus such as the one promoted by the development and evaluation process for mHealth [71] was prominent across the selected literature, directly supporting this principle. However, the aims and methods to accomplish this were often vaguely and partially described. For example, business modeling [70,86] approaches have been used for research (Athilingam et al [47]), but only for an initial conceptualization of the technology (a first concept of the solution that still requires validation, as defined by the iterative software design process [73]). A highlighted example of development intertwined with implementation was the aim to provide remote delivery of system refinements as proposed in the iterative refinement and patient participatory approach applied by Triantafyllidis et al [52]. This approach intended to facilitate continuous system updates without the use of valuable human resources. For this principle, only formative research (eg, focus groups in Duff et al [57]) and field studies (eg, Bartlett et al [56]) have been employed as methods that can be intertwined with the development process to understand and ideally increase the uptake of the technology.

### Development Integrates Theory, Evidence, and Participatory Approaches for Persuasive Design

The term of persuasive design, prominent in the field of human-computer interaction, was completely omitted in the included literature. However, it was evident that the integration of theory-, evidence- and person-based approaches (Band et al [49]) was used to increase persuasiveness. In other words, the selected studies implicitly set persuasiveness as part of their development aims—for example, by the proposed personalization and tailoring (Chantler al [53]) of the intervention, the creation of habits in the use of a technology, or the leverage on the hedonic (fun, pleasure) (Duff et al [57]) and automatic motivation (emotional reactions) of end users (Band et al [49] or Walsh et al [54]). In this regard, theoretical approaches were often related to theoretical modeling (eg, the logic model of Band et al [49]), while evidence was explored through preclinical or theoretical research (eg, literature reviews) conforming to the MRC’s guidelines [76-78]. As mentioned before, participatory or person-based approaches were more often applied as part of user-centered design. Hence, this revised principle highlighted how the included studies coped with the challenge of knowledge translation across different areas of research and its application to a specific aim (ie, integrating multidimensional ingredients that contribute to a common goal). To exemplify this, the aim for personalization and tailoring was derived from evidence that prioritized “the need to tailor...systems to user’s capacity and preferences” (Chantler et al [53]), rather than preassuming these aspects as key principles to increase technology persuasiveness via the facilitation of task support (as proposed by the persuasive systems design model [11]). One trade-off made apparent by this revised principle and example is that the identified approaches were not related to theory developed specifically for technology-based interventions, and therefore their application to this area seemed to be open to the interpretation of researchers and developers.

### Development Requires Continuous Evaluation Cycles

The requirement of continuous evaluation cycles in eHealth development revealed a contradiction within the included literature. The contradiction was outlined by the MRC’s [76-78] proposed stepwise development of complex interventions, as opposite to its own practical recommendation to undertake a parallel approach that can integrate stages with distinct aims into larger phases of development. For example, a large phase of development can include preclinical or theoretical research (eg, understanding the users and their environment through literature reviews) [76-78], early solution finding (eg, discussing solutions with the target group as defined by the iterative design model [58]), and an initial theoretical conceptualization (as defined by the development and evaluation process for mHealth [71]) or modeling of the eHealth technology and its components (eg, deciding on the theoretical basis and proposing how an intervention could work) [76-78]. In practical terms, evaluation cycles were often defined by either the choice of an agile (rapid and cyclical stages) or waterfall approach (long and sequential stages) to product development (as discussed by Srinivas et al [59]). This principle also highlighted the importance of integrating interdisciplinary methods (as proposed by Walsh et al [54]) that accommodate to the planned evaluation cycles. In this regard, the creation of an evaluation plan (as in intervention mapping [74]), where variables are defined in a measurable way in relation with the intervention objectives, methods, and strategies, seemed to be a key phase to bridge early design with formative evaluation processes of eHealth. To apply continuous evaluation cycles, the included studies made wide use of user-centered design methods [87,89] such as usability testing but also other frameworks such as realistic evaluation [84], which is a theory-driven approach to evaluate the complexity of social programs (applied by Bartlett et al [56]).

## Discussion

### Principal Findings

The findings of this review confirm and exemplify the remarkable challenges posed by the multidisciplinary gap in the field of eHealth. Mainly, the review listed 43 multidisciplinary frameworks, models, theories, and guidelines that have informed interventions within the scope of eHealth applications to self-management of CVD. Multidisciplinary approaches were often integrated and aimed to create a fit between users, the content of an intervention, and its context. The following sections summarize and assess the contributions of the principal findings with prior and related works.

### Bridging the Multidisciplinary Gap in Electronic Health Research and Development

In terms of development, the findings of this review place the integration of theory-, evidence- and participatory approaches to inform persuasive design as a newly generated overarching principle [49]. To do this, the studies often integrated knowledge from several disciplines, which in general has been argued as positive and desirable for eHealth [2]. However, in terms of design, one downside from the selected studies was that the approaches considered were often constrained to behavioral or sociological perspectives that were not focused on increasing the use and uptake of technology. In terms of implementation, this review suggests the importance of interdisciplinary methods that integrate broad perspectives such as the socioecological, sociotechnical [80,85], or business modeling [70,86] approaches. Specifically, the importance of workflow for the success of eHealth interventions has also been observed in another review [114]. Workflow can be defined as the way people interact with their work, communication pathways, and other people [114]. The inclusion of novel technological tools in the workflow of patients and health care providers was addressed in the reviewed studies through the lens of models such as the systems engineering initiative for patient safety 2.0 [85] framework or the domestication of technology theory [97,98]. For evaluation of eHealth, the reviewed literature acknowledged the iterative nature of this process, but some of the identified approaches seemed to still be restrained by fixed stages of postdevelopment testing of effectiveness. It must be noted that these fixed research programs can hinder the adaptability of interventions to the dynamic and flexible reality of the patients [115]. In this light, a previous review on the adoption of self-management solutions has also showed that a broad “consideration of preconceived barriers and facilitators for adoption” might be too simplistic, because what is perceived as a barrier or facilitator for one individual could have the opposite effect for another [116]. To maximize adoption, it is therefore recommended to iteratively reevaluate key social, motivational, cultural, moral, and financial factors [116]. The continuous evaluation of these factors can be matched with participatory and user-centered principles.

### Challenge of Reporting Intervention Content and Design

Overall, the findings of this review are in line with the general literature addressing several advantages to the use of theoretical frameworks for eHealth development and design and the different ways in which they can be operationalized [3]. However, the major challenge of adequate reporting of intervention design and content was also recognized (as discussed by Walsh et al [54] and Srinivas et al [59]). The lack of specification of the underlying approaches and their operationalization is still “suspected to be an artifact of publishing conventions and space constraints, as much as if not more than the nature of actual research being performed” [59]. All in all, the review included exemplary cases of publications with rich conceptual and descriptive data about eHealth development and design [46,49,54,56,57,59,60].

### Strengths and Limitations

This is considered to be the first metaethnography focused on bridging knowledge from multidisciplinary fields of science to better understand and improve eHealth research and development approaches. The review made great efforts to follow a thorough, systematic, multilevel approach [28], adhere to recently developed guidelines [27], and be informed by similar studies [22,25,26,33-35,37,38,43,117-121]. Although the number of papers included was relatively low, metaethnography is a complex methodology and synthesis process that entails numerous challenges and limitations [122]. For this review, a main limitation in the search phase was that no efforts were made to contact the authors to request additional information on their studies. This would have added additional time constraints that were not seen as feasible. For the same reason, although reference tracking was originally planned [28], no further inclusions through this method were considered. Although it was an exclusion criterion, it could arguably be seen as a limitation that some studies were excluded because they did not explicitly describe their underlying approaches. Including more papers could have arguably added new perspectives to the synthesis, but the added time to the interpretive task would have been too burdensome. In the translation phase, several concepts and themes required a high level of interpretation and study contextualization acquired by rereading the articles several times and with different intentions (eg, for data extraction, comparison, or verification) [25]. The main reviewer applied this approach, but co-reviewers followed a sequential approach focused on validation or identification of inconsistencies. Finally, it should also be noted that the key ingredients were sometimes extracted from the sources cited by the selected studies. Hence, the review could have missed updates and refined assumptions or principles. For example, the intervention mapping protocol has been continuously upgraded in comparison with the cited source of the selected studies [123].

### Conclusions and Recommendations

The multidisciplinary gap naturally constrains eHealth research and development to the structures and perspectives of discipline-specific frameworks that often miss key factors of the complex reality in health care. A holistic approach to the problem should consider multidisciplinary principles, such as those outlined by this review, to better define, structure, and report the underlying approaches to research and development of future eHealth interventions. The principles of the CeHRes roadmap mapped fairly well to what has been done in the selected literature. Positively, the use of participatory, user-centered design, and continuous evaluation cycles were commonly applied principles. On the contrary, less attention was given to the integration of implementation in the development process and implications of the new eHealth-based health care infrastructures as a whole. The integration of theory and evidence to inform (persuasive) design was an important principle that arose from the included studies, but the frameworks or models used to this purpose are not focused on creating a fit between human and technology.

Overall, it is recommended that researchers and developers make explicit and concrete statements about their approaches to eHealth. For instance, once a thoughtful decision has been made on guiding frameworks, models, or theories, it would be useful to also underline the holistic principles that are considered valuable by the research team (eg, will the approach consider existing evidence and theory or will it be solely guided by new data?). Unfortunately, there are no gold standards to report the content of eHealth interventions, beyond the CONSORT-EHEALTH checklist v.1.6 [31,32], which is specific to trials, and even less so to report underlying guiding principles. In the future, clearer operationalization (and reporting) of guiding frameworks and theoretical models is seen as vital to advance such knowledge in the field, as better predictive theories could provide an answer to the question “what works, for whom, in what settings, to change what behaviors, and how?” [124]. By and large, both theory and evidence must converge to determine the most effective mechanisms for technology-supported interventions. To accomplish this and move beyond what can be learned from published literature, holistic approaches can integrate patient-centered studies with consolidated knowledge from expert-based approaches (eg, via Delphi or other group decision-making methods [125]).

Finally, many questions still remain regarding the optimal use and advantages of specific frameworks or theories for eHealth development. Future reviews could aim to compare the effectiveness of theory-based eHealth interventions with those that do not make use of any. Moreover, more exploratory work is needed to understand how different frameworks or theories are more relevant or useful for specific settings and contexts (eg, which types of theories or frameworks are better suited to inform remote care interventions and why?).

## Appendix

Multimedia Appendix 1 Selection criteria and data extraction form.

Multimedia Appendix 2 Overview of relation, translation, and the synthesis process.

Multimedia Appendix 3 Excluded articles at full-text screening.

Multimedia Appendix 4 Characteristics of included articles.

Multimedia Appendix 5 Quality appraisal of included studies.

Multimedia Appendix 6 List of identified and translated metaphors.

Multimedia Appendix 7 Frameworks and models that informed the system’s development, implementation, or evaluation and their operationalized key ingredients by included studies.

Multimedia Appendix 8 Theoretical models that informed the system’s content and their operationalized key ingredients by included studies.

Multimedia Appendix 9 Comparison of included projects and their underlying approaches.

Multimedia Appendix 10 Perceived level of clarity and extent of the reported data related to development and design in included articles and projects.

Multimedia Appendix 11 Conceptual networks of clustered metaphors.

## Abbreviations

CeHRes: Center for eHealth Research
CHF PSMS: Congestive Heart Failure—Personalized Self-Management System
CONSORT-EHEALTH: Consolidated Standards of Reporting Trials of Electronic and Mobile Health Applications and Online Telehealth
CVD: cardiovascular diseases
eHealth: electronic health
eMERGe: Meta-ethnography Reporting Guidance
HOME BP: Home and Online Management and Evaluation of Blood Pressure
MRC: Medical Research Council
PRISMA: Preferred Reporting Items for Systematic Review and Meta-Analyses
SMASH: Smartphone Medication Adherence Stops Hypertension
SUPPORT HF: Seamless User-Centered Proactive Provision of Risk-Stratified Treatment for Heart Failure
