# Supplementary material for: Supporting Self-Management of Cardiovascular Diseases Through Remote Monitoring Technologies: Metaethnography Review of Frameworks, Models, and Theories Used in Research and Development
Source: J Med Internet Res. 2020 May 21;22(5):e16157. doi: 10.2196/16157 (PMC7273239; doi:10.2196/16157)
Supplement: Multimedia Appendix 1 [file jmir_v22i5e16157_app1.docx]

Multimedia Appendix 1 – Selection criteria and data extraction form

## Inclusion (✓) and exclusion criteria (🗶)

* Criteria was hierarchically ordered, thus during full text review and in case of multiple exclusion reasons, it was reported based on this sequence (e.g., 1a before 1c)

----------

**Population/Context**

✓1a. Focuses on cardiovascular diseases (Also when specifically mentioned: heart failure, hypertension, atrial fibrillation, coronary artery disease, peripheral artery disease; Include also if focused on CVD risk factors as long as the target is clear and specific)

✓1b. Focuses or includes target groups located outside the clinical setting (e.g., at home or in a community)

🗶1c. Target group is treated mainly within a clinical setting (e.g., hospital inpatients)

**Intervention**

✓2a. Refers to an eHealth supported intervention that focuses or includes self-management support as a key component (Consider as equivalent terms: self-care, self-treatment, self-regulation, self-help, self-monitoring, self-medication; Include if mentioned in terms of the patient's perspective: disease management, disease controllability)

✓2b. The eHealth technology provides feedback to the patient based on self-monitoring data (collected via remote monitoring technologies such as wearables, blood pressure monitors, or weigh scale)

🗶2c. Focuses only on disease management from the healthcare provider's (HCP) perspective

🗶2d. Feedback is provided only via remote consultation with the HCP (e.g., by chat, telephone or video)

🗶2e. Self-monitoring is performed only by self-reports and not by the use of a remote monitoring technology

**Content of interest**

✓3a. Refers to the use of a framework, model or theory applied to develop, implement, or evaluate the eHealth technology (Include when specifically mentioned: participatory design, persuasive design, user or human centred, and business modelling)

🗶3b. The framework, model or theory is focused on healthcare service delivery (e.g., Chronic Care Model) or economic evaluation rather than on design or development guidelines for an eHealth supported intervention (e.g., participatory design, iterative evaluations)

🗶3c. **[Full text screening only]** The framework, model or theory is not sufficiently described or not enough information is provided about its operationalization. **Sufficiency** is determined if two sub-criteria are met: **I)** The article includes a section that describes how a framework, model or theory was operationalized or applied for the development, implementation or evaluation of the intervention (e.g., a design/intervention section that describes development procedures, or a methods section that describes an –iterative– evaluation process). **II)** The article provides a description or background information about the underlying framework, model or theory applied (within the same text, via supplementary materials or by references to the original sources).

**Study characteristics**

✓4a. Quantitative or qualitative (Including protocols, reviews and articles published in conference proceedings) (Abstracts of conference proceedings are included if they hint towards an article of possible interest, which can be screened during the full text stage)

🗶4b. It is published before 2008

🗶4c. **[Full text screening only]** Full text cannot be accessed

🗶4d. **[Full text screening only]** It is not written in English, Dutch or Spanish language

🗶4e. **[Full text screening only]** It is a doctoral thesis

🗶4f. **[Full text screening only]** It is not peer reviewed

## Data extraction form

The present data extraction form is mainly based on the CONSORT-EHEALTH checklist v.1.6., furtherly adapted to facilitate the aim of the present review. A relation between the research questions, the data extraction categories, and the type of data extracted is presented below:

| **Research question** |  | **Data extraction category** | **Type of data extracted** |
| --- | --- | --- | --- |
| 1. What frameworks, models, or theories have been used to develop, implement, or evaluate interventions to support self-management of patients with CVD through the use of remote monitoring technologies? | 🡺 | I. Study description  II. eHealth intervention  III. Underlying framework, model or theory | - Methodological and contextual data: e.g., context and participants of study; underlying theoretical approach - Key ingredients of applied framework, model, or theory |
| 1. What are the key ingredients of these frameworks, models, or theories that inform or guide the system’s ***a)*** development, evaluation, or implementation; ***b)*** content design to promote behavior change and technology adoption; or ***c)*** proposed effects in terms of health-related outcomes? | 🡺 | IV. Characterization of key ingredients | - Key ingredients to enable intervention effectiveness in terms of behavior change, technology adoption, or health-related outcomes |
| 1. To what extent do the key ingredients of these frameworks, models, or theories fit with the five principles of a holistic research and development approach of eHealth? As proposed by the CeHRes Roadmap. | 🡺 | IV. Characterization of key ingredients | - Holistic principles for eHealth development as proposed by the CeHRes Roadmap |
|  |  | V. Overview of key metaphors | - Primary and secondary key metaphors |

The form was first piloted with a sample of selected studies and then iteratively adjusted as necessary during the review process. The main changes from the template reported in our published protocol (Cruz-Martinez et al. 2019) are listed in the table below:

| **Changes to research questions and data extraction form** |
| --- |
| - Research questions were refined throughout the metaethnography to better communicate the raw data of the review (e.g., key ingredients of frameworks) and the aims of the synthesis (e.g., characterization of key ingredients) - In section IV, one of the CeHRes Roadmap’s holistic principles used by the main reviewer during data extraction (phase 3) changed. Initially, the second principle stating that ‘eHealth development creates new infrastructures for improving health care, health, and well-being’ was thought to be self-evident given the narrow search and selection process (i.e., all selected studies would implicitly entail a new infrastructure). In turn, the underpinning pillar of ‘Business modelling’ was used in the previous form. However, preliminary analysis found that almost all studies neglected this. Therefore, the choice was reversed and the second principle was considered again for all phases of the review. The observed gap was noted in the resulting synthesis. - In section IV, pointers were added to data elements when it was necessary for the reviewer to make a certain categorization of the data (e.g., differentiating between ‘parameters of effectiveness’ or ‘practical applications’ in the characterization of behavior change key ingredients). - Section V was added to facilitate an overview of key metaphors identified from each study. This format was used to translate all key metaphors into a Microsoft Excel format, which was used during the translation process. - Minor changes in the language and the format of the data extraction form were applied. |

| **I. Study description** | | |
| --- | --- | --- |
| This section is focused on collecting key **methodological** **and contextual** information of the study. The data extracted here is thus strictly derived from the paper. However, a minor degree of interpretation is implicit by the reviewer when organizing the data. Notes are added when necessary to provide justification and transparency to the process. | | |
| **1a** Title |  | |
| **1b** Author(s) |  | |
| **1c** Affiliation(s) | i) Author(s) affiliations. *Include institutions and countries and mark the corresponding author.* | |
|  |  | |
|  | ii) Reported conflicts of interest | |
|  |  | |
| **1d** Year of publication |  | |
| **1e** Journal | i) Name |  |
|  | ii) Focus and scope. *Extracted from journal’s website.* | |
|  |  | |
| **1f** Target condition(s) |  | |
| **1g** Aim | i) General study aim. *Including description of overarching or related project(s).* | |
|  |  | |
|  | ii) Research question(s) and study objective(s) | |
|  |  | |
| **1h** Design | i) Study classification. *Based on the Oxford Centre for Evidence-based medicine (*[*https://www.cebm.net/2014/04/study-designs/*](https://www.cebm.net/2014/04/study-designs/)*). If necessary, clarify if design is cross-sectional or longitudinal, prospective or retrospective, single or multi-group, randomized or non-randomized, and if blinded or open-label.* | |
|  | Descriptive/Analytic/Not applicable; … | |
|  | ii) Setting. *General description of the organization of the study, including location(s) were study was conducted.* | |
|  |  | |
|  | iii) Institutions involved. *Hospitals, universities or other organizations involved. Which roles they had and how (if) these affiliations were displayed to participants.* | |
|  |  | |
| **1i** Participants | i) Eligibility criteria | |
|  |  | |
|  | ii) Recruitment procedure. *How participants were recruited (online vs. offline). If online-only, clarify if there were any anonymization measures. How participants were briefed for recruitment and in the informed consent procedures. Ethical approval information (if applicable).* | |
|  |  | |
|  | iii) Sample characteristics. *Baseline demographics, size, and other reported data.* | |
|  |  | |
|  | iv) Computer / Internet literacy | |
|  |  | |
| **1j** Study outcomes | i) Primary outcome(s) | |
|  |  | |
|  | ii) Secondary outcomes(s) | |
|  |  | |
|  | iii) Process outcome(s). *Including use or adoption metrics and how they were defined (e.g., what was considered a ‘session’).* | |
|  |  | |
|  | iv) Data collection method(s), tools, and analysis. *How outcomes were (self-)assessed, measured, monitored, and analyzed.* | |
|  |  | |

| **II. eHealth intervention** | |
| --- | --- |
| This section is focused on collecting key **methodological** **and contextual** information of the eHealth intervention described within the study. The data extracted here is thus strictly derived from the paper. However, a minor degree of interpretation is implicit by the reviewer when organizing the data. Notes are added when necessary to provide justification and transparency to the process. | |
| **2a** Name |  |
| **2b** Developers, sponsors, and owners | i) Developers & sponsors. *Clarify the relation of the study team towards the system being*  *evaluated. For example, if the authors are distinct from or identical with the developers of the intervention.* |
|  | Developers:  Sponsors: |
|  | ii) Owners. *Include names, credential and affiliations. Clarify if intervention/technology is still available and provide links for additional information if necessary.* |
|  |  |
| **2c** Development aim | i) General aim of development |
|  |  |
|  | ii) Specific objectives of development |
|  |  |
| **2d** Device(s) and main technical functionalities | *Include description (if) of interoperability between technological devices.* |
|  |  |
| **2e** Main content features | i) Summary of main content features |
|  |  |
|  | ii) In-depth description of content components. *Including behavior change techniques or persuasive design features with author(s) definitions. How (if) each component was tailored to individual circumstances.* |
|  |  |
| **2f** Mode of delivery and implementation | i) How participants accessed the intervention. *Required credentials to access the intervention components (e.g., web-based platform). Include if they had to pay (or were paid) to become members of a specific group.* |
|  |  |
|  | ii) Use parameters. *Intended ‘doses’ and optimal timing for use.* |
|  |  |
|  | iii) Instructions of use given to participants. *Such as timing, frequency or heaviness of use.* |
|  |  |
| **2g** Feedback | i) Main description of feedback process and features |
|  |  |
|  | ii) Level of human involvement. Automated only vs. blended care. *Number, specific roles and type of assistance of humans involved (e.g., care providers, health professionals, technical assistants), and medium by which involvement occurred.* |
|  | Automated/Blended care |
|  | iii) Communication channels. *Synchronous vs asynchronous. Textual vs. visual. If prompts or reminders were used and what triggered them (e.g., frequency).* |
|  |  |
|  | iv) Presentation principles or strategies. *Descriptive information about the design and aesthetics of the intervention. Include principles or strategies used in page design, as well as basic information such as average amount of text on pages.* |
|  |  |
| **2h** Development process | i) Historical summary. *Narrative commentaries, notes, and observations from the authors about the development process not derived from the underlying framework, model or theory.* |
|  |  |
|  | ii) Formative evaluations. *Include list of reported methods with keywords (e.g., focus groups, usability testing).* |
|  | Method 1  Method 2  … |
|  | iii) Digital preservation. *Include URL of the application, archived public materials (links to screenshots/videos/demo pages).* |
|  |  |
|  | iv) Published studies or grey literature. *List of related works by project. Include and mark references that were also screened for inclusion in the present review.* |
|  | Backward: Cited in paper  Forward: Cited by (Journal X, NCBI, WoS, Scopus) |
| **2i** Intervention results | i) Results on primary and secondary outcome(s) |
|  |  |
|  | ii) Report on process outcome(s). *Including attrition.* |
|  |  |
|  | iii) Report on technical problems or unintended effects. *Not only including physical “harm” to participants, but also incidents such as perceived or real privacy breaches and other unexpected/unintended incidents.* |
|  |  |
|  | iv) Interpretation and principal findings. *Presented and summarized as per study question. Include limitations of study/project when reported, as well as unanswered new questions and suggestions for future research.* |
|  |  |

| **III. Underlying framework, model or theory** | | |
| --- | --- | --- |
| This section contains a higher degree of interpretation by the reviewer. Namely, to identify the most suitable **description** of a framework, model or theory applied in the study, and to better reflect its **operationalization.** The reviewer also provides a **categorization** of the framework, model or theory**,** and identifies its general **approach to eHealth** according to the study. | | |
| **3a** Name |  | |
| **3b** Description | i) Original source(s) referenced by the study author(s) | |
|  |  | |
|  | ii) General description. *If provided by selected study, otherwise cite original source.* | |
|  | [Described by study authors] / [Extracted from original source] | |
|  | iii) Key framework, model or theory ingredients. *List of main principles, assumptions, concepts, components, parameters, conditions, phases or stages. Include definitions of each preferably provided by selected study, otherwise cite original source.* | |
|  | [Described by study authors] / [Extracted from original source] | |
|  | iv) Visual representation. *If applicable and provided by selected study, otherwise cite original source.* | |
|  | [Provided by study authors] / [Extracted from original source] | |
| **3c** Operationalization | List author(s) statements regarding use or operationalization of the framework, model or theory. *Key ingredients listed in* ***3b-iii*** *are highlighted in bold.* | |
|  | **Key ingredient** (Framework, model or theory)  Statement 1  Statement 2  … | |
| **3d** Categorization | Mark with an X if it meets any of the following definitions: | |
|  |  | Framework. *An* ***extensive*** s*et of principles, such as assumptions, constructs, quality criteria, and ideas that can guide research and development. It can also contain strategies such as guidelines, design heuristics, and methods to assist on a* ***staged****,* ***phased****, or* ***time*** ***oriented*** ***process****.* |
|  |  | Model. *A* ***simplified representation*** *of a reality, hypothesis, theory, or knowledge. It can contain a set of concepts, statements, or both that specify how constructs relate to each other. Although, it can be both* ***‘precise and quantified’*** *or ‘****imprecise and qualitative’****.* |
|  |  | Theory. *Set of concepts and/or statements with* ***specification of how phenomena relate to each other****. Theory provides an organizing description of a system that accounts for what is known, and* ***explains and predicts phenomena****.* |
| **3e** Approach to eHealth | Mark with an X if framework, model or theory was applied to any of the following: | |
|  |  | Development. *Refers to an* ***iterative process*** *of development of eHealth, entailing activities for pre-design, design, implementation and evaluation.* |
|  |  | Implementation. *Refers* ***exclusively*** *to activities that are undertaken to realize the adoption, dissemination and long-term use of a product in its intended context.* |
|  |  | Evaluation. *Refers* ***exclusively*** *to formative evaluation or summative evaluation.* ***Formative evaluation*** *englobes the activities throughout the entire development process that provide ongoing information on how to improve the development process, outcomes of activities and eHealth technology.* ***Summative evaluation*** *is the development phase which studies the influence and role of the technology on health, the context, behavior and stakeholder perspective via evaluations of impact and uptake of the technology.* |

| **IV. Characterization of key ingredients** | | |
| --- | --- | --- |
| This section contains the highest degree of interpretation by the reviewer. Namely, to interpret or characterize the key ingredients of a framework, model or theory according to holistic principles of eHealth development as proposed by the **CeHRes Roadmap**. Alternatively, to interpret or characterize them as proposed key ingredients to enable intervention effectiveness of an eHealth intervention to support self-management of CVD, whether in terms of **behavior change**, **technology adoption**, or **health-related** **outcomes**. | | |
| **4a** Key ingredient(s) addressing CeHRes Roadmap principles | Mark with an X if a principle is met and name the key ingredients **(3b-iii)** that relate to it: | |
|  |  | Participatory development. *The structural cooperation of eHealth developers with potential end users and other stakeholders during its development.* ***Including user or human centred design****, both generally defined as a framework that aims to develop solutions to problems by involving the human perspectives in all steps of the process, via observing the problem within context, brainstorming, conceptualizing, developing and implementing the solution.* |
|  | Ingredients | Framework, model or theory: Key ingredient 1; Key ingredient 2; … |
|  |  | Creation of new ecosystems for improving health and healthcare. *The creation of novel structures and processes for health and healthcare through eHealth. Some of these changing characteristics are a change in place-dependent delivery, a new division of labor, new regulations for the use of technology financing, and a shift from hospital to home-based care.* |
|  | Ingredients |  |
|  |  | Intertwined with implementation. *The inclusion in the development process of activities that are undertaken to realize the adoption, dissemination and long-term use of a product in its intended context.* ***Including business modelling****, defined as how an organization creates, delivers and captures values. It can be a conceptual and analytical framework to discuss the added values of an eHealth intervention.* |
|  | Ingredients |  |
|  |  | Persuasive technology design. *Designing technology that aims to reinforce, change, shape or influence behavior and attitudes by being compelling and without being coercive or deceptive.* |
|  | Ingredients |  |
|  |  | Continuous evaluation cycles. *Employment of iterative design methodologies based on a cyclic process of needs assessment, prototyping, testing, analyzing and refining a product, during which changes and refinements are made to the product based on the results of the most recent iteration of a design.* |
|  | Ingredients |  |
| **4b** Key ingredients proposed to enable effectiveness of an eHealth intervention to support self-management of CVD | Behavior change. *Key ingredients to enable intervention effectiveness in terms of* ***behavior change****, such as* ***practical applications*** *or* ***parameters of effectiveness*** *of behavior change methods.* ***Practical applications*** *are the translations of theoretical methods of behavior change to practical intervention elements. Applications are by definition specific, ideally tailored to populations, intervention contexts and behavioral domains.* ***Parameters for effectiveness*** *are the characteristics that a practical application must manifest for it to accurately reflect the theoretical method. When these parameters are lost in translation from method to application, effective behavior change is undermined and may even result in counterproductive effects. Evidence for the existence of such parameters can range from theoretical to meta-analytical.* | |
|  | **Parameters of effectiveness of behavior change methods**  Key ingredient 1  Key ingredient 2  …  **Practical applications of behavior change methods**  Key ingredient 3  Key ingredient 4  … | |
|  | Technology adoption. *Key ingredients to enable effectiveness in terms of* ***technology adoption****, such as those that aim to increase the engagement, use, adherence, uptake or adoption of the technology. For example, the use of* ***profiling mechanisms****, defined as ingredients that are employed to adapt an eHealth intervention to the characteristics of an individual or cohort (e.g., motivation levels as measured in a pre-test).* | |
|  | **Profiling mechanisms**  Key ingredient 1  Key ingredient 2  … | |
|  | Health-related outcomes. *Key ingredients to enable intervention effectiveness in terms of the* ***outcomes*** *of an intervention that directly or indirectly have an impact on the health or wellbeing of the target group. For example, changes in health parameters (e.g., blood pressure control), risk factors (e.g., weight), or performance of self-care or healthy behaviors (e.g., physical activity levels).* | |
|  |  | |

| **V. Overview of key metaphors** | | | | | | | | | | | |
| --- | --- | --- | --- | --- | --- | --- | --- | --- | --- | --- | --- |
| This section provides an overview of the raw data for the synthesis, referred to as the primary and secondary key metaphors of the selected papers. | | | | | | | | | | | |
| Primary key metaphors. Key ingredients of frameworks, models and theories applied in eHealth studies and projects aiming to support self-management of CVD. | | | | | | | | | | | |
| *Source* | *Key ingredient* | *Description* | *Exemplary quote(s)* | *CeHRes holistic principles* | | | | | *Effectiveness* | | |
|  |  |  |  | *PD* | *NE* | *IwI* | *PTD* | *CeC* | *BC* | *TA* | *O* |
|  |  |  |  |  |  |  |  |  |  |  |  |
|  |  |  |  |  |  |  |  |  |  |  |  |
| Secondary key metaphors. Key phrases, ideas, concepts, perspectives, organizers, and/or themes of a study that are not derived from an underlying framework, model or theory. | | | | | | | | | | | |
| *Concept/Idea* | | *Description* | *Exemplary quote(s)* | | | | | | | | |
|  | |  |  | | | | | | | | |
|  | |  |  | | | | | | | | |
