# Supplementary material for: Supporting Self-Management of Cardiovascular Diseases Through Remote Monitoring Technologies: Metaethnography Review of Frameworks, Models, and Theories Used in Research and Development
Source: J Med Internet Res. 2020 May 21;22(5):e16157. doi: 10.2196/16157 (PMC7273239; doi:10.2196/16157)

Multimedia Appendix 2 – Overview of relation, translation, and the synthesis process

| Phases | Key Output | Activity | Data from | Reviewers^a^ |
| --- | --- | --- | --- | --- |
| 4 –Relation of selected studies | Tabular comparison | Summarize data and create tables | All studies |  |
|  | Conceptual networks | Visualize potential relations | All studies |  |
|  | Data extraction matrix | Comparing perceived clarity of reported data | All studies | RAA^b^ |
|  | Bibliometric analysis | Conduct explorative analysis | All studies | JMN^c^ |
| 5 – Translation of selected studies^d^ | Key metaphors; Reciprocal and refutational translations | Piloting and refining collective interpretation and translation | Athilingam et al 2018a^e^ | JW, JGP |
|  |  | Collective interpretation and translation of two key studies and two non-key studies | Duff et al 2018^e^, Walsh et al 2018a^e^; Baek et al 2018, Walsh et al 2018b | JW, RAA |
|  |  | Collective interpretation and translation of key studies | Bartlett et al 2014^e^, Srinivas et al 2017^e^, Band et al 2017^e^, Villalba et al 2009^e^ | JW, RAA |
|  |  | Collective interpretation and translation of non-key studies | Athilingam et al 2016, Athilingam et al 2018b, Band et al 2016, Bradbury et al 2017 McGillicuddy et al 2012 Chantler et al 2016 Rahimi et al 2015, Triantafyllidis et al 2015 | JW, RAA |
|  | Clustering of metaphors | Collective clustering of key metaphors | All studies | JW, RAA |
| 6 –Synthesis process | Synthesized translations | Development of overarching concepts, themes, and principles | All studies | JW, JGP |
|  | Line-of-argument synthesis | Development of line-of-argument | All studies | JW, JGP |
| ^a^The main reviewer (RRCM) was involved in all phases and iteratively revised all key output.  ^b^RAA co-reviewed a first sample of studies and provided feedback on the first versions of the data extraction form and the Data Extraction Matrix.  ^c^JMN conducted the bibliometric analysis based on input from the main reviewer.  ^d^The translation was mainly conducted using conceptual networks in ATLAS.ti (visualization of relations and themes) and Microsoft Excel (columns with terms, definitions, sources, and independent characterization of metaphors).  ^e^Key studies were those considered to have rich descriptive data for the synthesis. | | | | |

The process of translation began by identifying the final sample of studies with rich conceptual or descriptive data for the synthesis. Rich conceptual or descriptive data are those which provide sufficient detail that can be further interpreted to develop conceptual insights. By this metric, 7 out of 17 (41%) studies ([see table](#_Overview_of_activities)) were labelled as a “key study”, all of them addressing different projects: HeartMapp, MedFit, PATHway, CHF PSMS, Engage, HOME BP, MyHeart. Eight studies ([see table](#_Overview_of_activities)) still made significant contributions to the synthesis because they contained data of interest (eg, additional metaphors), although not as extensively described as in the other studies. One article (Band et al 2016) was a research protocol that provided only one secondary key metaphor. In sum, although the richness of data varied across selected studies, no exclusions were made for the synthesis. The figure below is a worked example of using ATLAS.ti’s conceptual networks to visualize the relation and translation links between the data.

# Example of a conceptual network visualizing the relation, translation, and synthesis of key metaphors
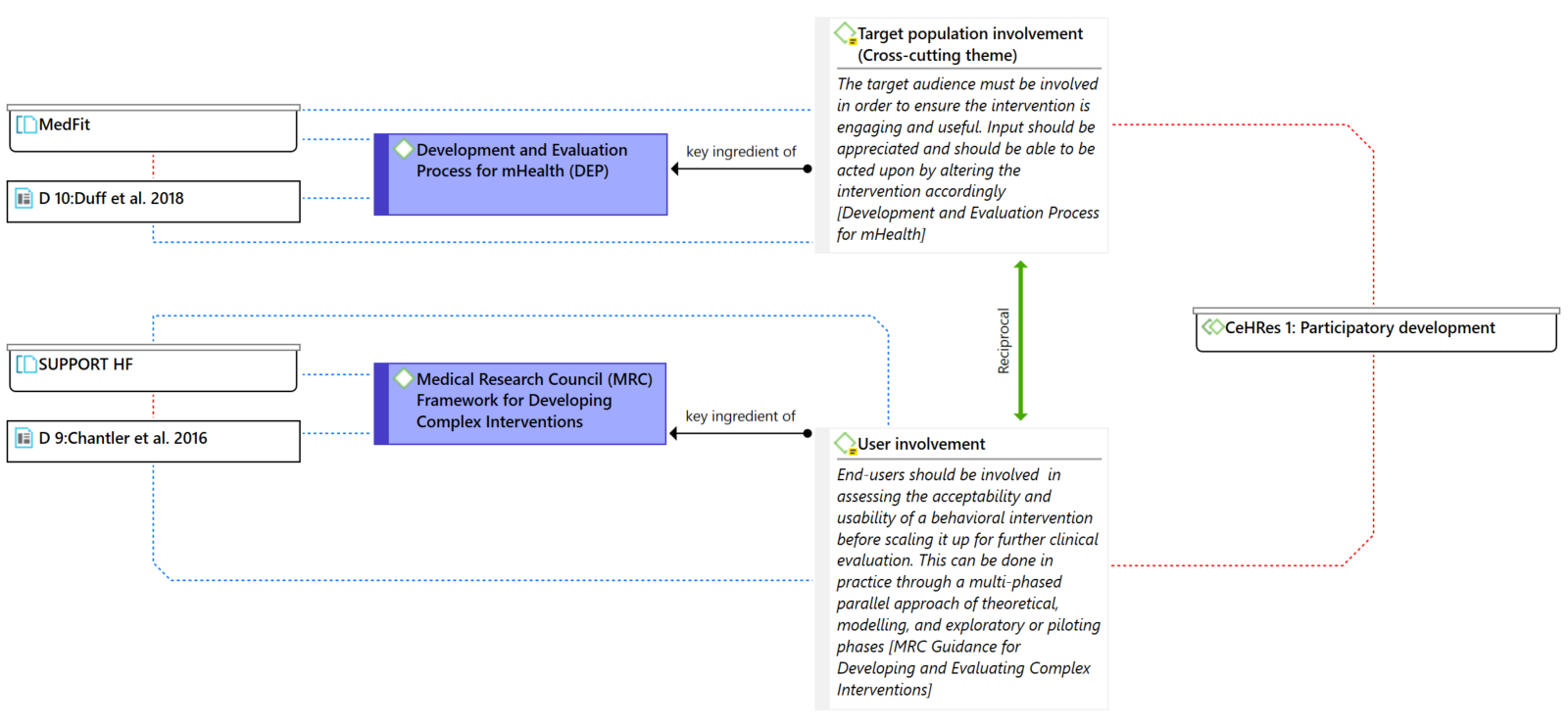

Supplement: Multimedia Appendix 2 [file jmir_v22i5e16157_app2.docx]
