# Supplementary material for: Supporting Self-Management of Cardiovascular Diseases Through Remote Monitoring Technologies: Metaethnography Review of Frameworks, Models, and Theories Used in Research and Development
Source: J Med Internet Res. 2020 May 21;22(5):e16157. doi: 10.2196/16157 (PMC7273239; doi:10.2196/16157)
Supplement: Multimedia Appendix 3 [file jmir_v22i5e16157_app3.docx]

Multimedia Appendix 3 – Excluded articles at full text screening

|  | Study | Reason for exclusion | Reviewers' notes |
| --- | --- | --- | --- |
| 1 | 2017 AAHFN 13th Annual Conference. Heart and Lung: Journal of Acute and Critical Care. 2017;46(3). | **Intervention**; 2a Doesn't focus or include self-management as key component |  |
| 2 | Acheampong F, Vimarlund V. Innovating Healthcare through Remote Monitoring: Effects and Business Model. International Journal of Information System Modeling and Design. 2016;7(1):49-66. doi: 10.4018/ijismd.2016010104. | **Intervention**; 2a Doesn't focus or include self-management as key component |  |
| 3 | Ahern DK, Stinson LJ, Uebelacker LA, Wroblewski JP, McMurray JH, Eaton CB, et al. E-health blood pressure control program. Journal of Medical Practice Management. 2012;28(2):91-100. | **Content of interest for the synthesis**; 3b Framework, model or theory is not focused on eHealth design or development | Refers only to a healthcare service model (Chronic Care Model). |
| 4 | Aidemark J, Askenas L, Martensson J, Stromberg A, Varajao J, Cunha M, et al. Challenges for Heart Failure Patients' Self-Care Systems Analysis of Patients' Needs. 2014;16:1256-64. doi: 10.1016/j.protcy.2014.10.141. | **Content of interest for the synthesis**; 3a No framework, model or theory reported |  |
| 5 | Alluhaidan A, Lee E, Alnosayan N, Chatterjee S, Houston-Feenstra L, Dysinger W, et al. Designing Patient-Centered mHealth Technology Intervention to Reduce Hospital Readmission for Heart-Failure Patients. IEEE Computer Society; 2015. | **Content of interest for the synthesis**; 3c Framework, model or theory is not sufficiently described or not enough information is provided about its operationalization | Fogg's behavior model is mentioned used for design but it is not sufficiently described how. Messages are used as an "external trigger". They elaborate on the messages provided by the system, but not much in relation to the model. |
| 6 | Alnosayan N, Lee E, Alluhaidan A, Chatterjee S, Houston-Feenstra L, Kagoda M, et al. MyHeart: An Intelligent mHealth Home Monitoring System Supporting Heart Failure Self-Care2014. 311-6 p. | **Content of interest for the synthesis**; 3c Framework, model or theory is not sufficiently described or not enough information is provided about its operationalization | Fogg's behavior model as being used for design but it is not sufficiently described how. The paper mention that Fogg´s behavior model is being used as the system sends messages to patients that aim to encourage self-care. |
| 7 | Alrawashdeh M, Zomak R, Al-Zaiti S, Sereika S, Parmanto B, DeVito Dabbs A. Clinicians' acceptance of interactive health technologies for self-management among patients with chronic cardiopulmonary disorders. Journal of Heart and Lung Transplantation. 2018;37(4):S37-S8. | **Intervention**; 2c Focuses on disease management from HCP perspective |  |
| 8 | Alshurafa N, Eastwood J, Pourhomayoun M, Liu JJ, Nyamathi S, Sarrafzadeh M. A Framework for Predicting Adherence in Remote Health Monitoring Systems. Bethesda, MD, USA: ACM; 2014. | **Content of interest for the synthesis**; 3a No framework, model or theory reported |  |
| 9 | Alshurafa N, Sideris C, Pourhomayoun M, Kalantarian H, Sarrafzadeh M, Eastwood J-A. Remote Health Monitoring Outcome Success Prediction Using Baseline and First Month Intervention Data. IEEE journal of biomedical and health informatics. 2017;21(2):507-14. doi: 10.1109/jbhi.2016.2518673. | **Content of interest for the synthesis**; 3a No framework, model or theory reported |  |
| 10 | Ammenwerth E, Modre-Osprian R, Fetz B, Gstrein S, Krestan S, Dörler J, et al. HerzMobil, an integrated and collaborative telemonitoring-based disease management program for patients with heart failure: A feasibility study paving the way to routine care. Journal of Medical Internet Research. 2018;20(4). doi: 10.2196/cardio.9936. | **Content of interest for the synthesis**; 3b Framework, model or theory is not focused on eHealth design or development | Refers to the 'DeLone and McLean Model of Information Systems Success' which was adapted (but not validated) as a survey to measure user satisfaction after a 3-months pilot. The model is not focused on design or development guidelines and is just used as a simple evaluation measure. |
| 11 | Andersen T, Kensing F, Kjellberg L, Moll J. From Research Prototypes to a Marketable eHealth System. Studies in health technology and informatics. 2015;218:9-14. | **Content of interest for the synthesis**; 3c Framework, model or theory is not sufficiently described or not enough information is provided about its operationalization | The paper mention that they use "Business Model Canvas" and "The Stage Gate Model", inspired by Scrum. The 'Stage Gate Model' is mentioned within the text as a way to tackle the challenge of integrating all stakeholders’ interests. The paper provides little further information on these models and how they are used. |
| 13 | Athilingam P, Osorio RE, Kaplan H, Oliver D, O'Neachtain T, Rogal PJ. Embedding Patient Education in Mobile Platform for Patients With Heart Failure Theory-Based Development and Beta Testing. Cin-Computers Informatics Nursing. 2016;34(2):92-8. doi: 10.1097/cin.0000000000000216. | **Intervention**; 2b Feedback to the patient is not based on self-monitoring data via remote monitoring technologies |  |
| 12 | Athilingam P. A mobile application to improve self-care of patients with heart failure application (HeartMapp) to improve self-management of patients with heart failure. Journal of Cardiac Failure. 2016;22:S102-S3. | **Content of interest for the synthesis**; 3c Framework, model or theory is not sufficiently described or not enough information is provided about its operationalization | Refers to the “Information, Motivation, and Behavioral skills” model but doesn't provide sufficient information. |
| 14 | Bai d, Cdg Dg System L, Ibm, Professional Computer Company L. 6th International Conference on Advances in Information Technology 2013, IAIT 2013. 2013;409. | **Intervention**; 2a Doesn't focus or include self-management as key component |  |
| 15 | Banchs JE, Scher DL. Emerging Role of Digital Technology and Remote Monitoring in the Care of Cardiac Patients. Medical Clinics of North America. 2015;99(4):877-96. doi: 10.1016/j.mcna.2015.02.013. | **Intervention**; 2a Doesn't focus or include self-management as key component |  |
| 16 | Bardram JE. Pervasive healthcare as a scientific discipline. Methods of Information in Medicine. 2008;47(3):178-85. doi: 10.3414/me9107. | **Population/Context**; 1a Doesn't focus or include CVD |  |
| 17 | Barley EA, Clifton A, Lee G, Norman IJ, O'Callaghan D, Tierney K, et al. The Space From Heart Disease Intervention for People With Cardiovascular Disease and Distress: A Mixed-Methods Study. JMIR research protocols. 2015;4(3). doi: 10.2196/resprot.4280. | **Intervention**; 2b Feedback to the patient is not based on self-monitoring data via remote monitoring technologies |  |
| 18 | Berry E, Silvera A, Whitney Downer W, Agar D, Beske Radford S, Chiromeras N, et al. Patient engagement improvement following implementation of a remote monitoring system. European Journal of Heart Failure. 2017;19:545. doi: 10.1002/ejhf.833. | **Content of interest for the synthesis**; 3a No framework, model or theory reported |  |
| 19 | Cairns A, Mort S, Tucker K, Leeson P, Mackillop L, Crawford C, et al. A novel self-management Intervention for adjustment of postnatal antihypertensive treatment2017; 38(Supplement 1):[850-1 pp.]. Available from: http://cochranelibrary-wiley.com/o/cochrane/clcentral/articles/838/CN-01468838/frame.html. | **Content of interest for the synthesis**; 3a No framework, model or theory reported |  |
| 20 | Cajita MI, Gleason KT, Hae-Ra H. A Systematic Review of mHealth-Based Heart Failure Interventions. Journal of Cardiovascular Nursing. 2016;31(3):E10-E22. doi: 10.1097/JCN.0000000000000305. | **Content of interest for the synthesis**; 3a No framework, model or theory reported |  |
| 21 | Cajita MI, Hodgson NA, Budhathoki C, Hae-Ra H. Intention to Use mHealth in Older Adults With Heart Failure. Journal of Cardiovascular Nursing. 2017;32(6):E1-E7. doi: 10.1097/JCN.0000000000000401. | **Content of interest for the synthesis**; 3a No framework, model or theory reported |  |
| 22 | Carrera PM, Lambooij MS. Implementation of out-of-office blood pressure monitoring in The Netherlands: From clinical guidelines to patients' adoption of innovation. Medicine (United States). 2015;94(43). doi: 10.1097/MD.0000000000001813. | **Intervention**; 2b Feedback to the patient is not based on self-monitoring data via remote monitoring technologies |  |
| 23 | Catherine Demers C, Wali S, Nguyen L, Mulvale G, McKay E, Mbuagbaw L, et al. Starting with the patient: Designing an electronic application with patient and caregiver feedback to promote heart failure self-care in the home setting. European Journal of Heart Failure. 2018;20:463. doi: 10.1002/ejhf.1197. | **Intervention**; 2b Feedback to the patient is not based on self-monitoring data via remote monitoring technologies |  |
| 24 | Chen CW, Fanjiang YY, Chiang YT, Ho CL. Mobile health program to promote self management in youths with congenital heart disease design and development of the cool randomized controlled trial2017; 27(4):[S89 p.]. Available from: http://cochranelibrary-wiley.com/o/cochrane/clcentral/articles/349/CN-01452349/frame.html. | **Content of interest for the synthesis**; 3c Framework, model or theory is not sufficiently described or not enough information is provided about its operationalization | Refers that 'both active **Intervention**s were designed based on self-regulation theory' but no sufficient information is provided. The citation is a conference session. |
| 25 | Claes J, Buys R, Woods C, Briggs A, Geue C, Aitken M, et al. PATHway I: design and rationale for the investigation of the feasibility, clinical effectiveness and cost-effectiveness of a technology-enabled cardiac rehabilitation platform2017; 7(6) (no pagination). Available from: http://cochranelibrary-wiley.com/o/cochrane/clcentral/articles/762/CN-01395762/frame.html. | **Content of interest for the synthesis**; 3a No framework, model or theory reported |  |
| 26 | Clarke DM, Perera DN, Casey MF. Psychological and behavioral contributions to rehabilitation and recovery in heart disease. 2016:957-79. doi: 10.1007/978-981-287-206-7_49. | **Intervention**; 2a Doesn't focus or include self-management as key component |  |
| 27 | Cornet VP, Daley CN, Srinivas P, Holden RJ. User-centered evaluations with older adults: Testing the usability of a mobile health system for heart failure self-management. 2017;2017-October:6-10. doi: 10.1177/1541931213601497. | **Intervention**; 2b Feedback to the patient is not based on self-monitoring data via remote monitoring technologies |  |
| 28 | Davidson T, McGillicuddy J, Mueller M, Brunner-Jackson B, Favella A, Anderson A, et al. Evaluation of an mHealth medication regimen self-management program for African American and Hispanic uncontrolled hypertensives2015; 5(4):[389-405 pp.]. Available from: http://cochranelibrary-wiley.com/o/cochrane/clcentral/articles/520/CN-01161520/frame.html. | **Content of interest for the synthesis**; 3c Framework, model or theory is not sufficiently described or not enough information is provided about its operationalization | Refers that the **Intervention** was developed using a patient and HCP-centered iterative design process and principles of SDT. However, no information is provided about how these were operationalized. |
| 29 | Dimengo J, Stegall G. Team-Based Care for External Telemonitoring in Patients with Heart Failure. Heart Failure Clinics. 2015;11(3):451-+. doi: 10.1016/j.hfc.2015.03.008. | **Content of interest for the synthesis**; 3a No framework, model or theory reported |  |
| 30 | Dorough AE, Winett RA, Anderson ES, Davy BM, Martin EC, Hedrick V. DASH to Wellness: Emphasizing Self-Regulation Through E-Health in Adults With Prehypertension. Health Psychology. 2014;33(3):249-54. doi: 10.1037/a0030483. | **Intervention**; 2b Feedback to the patient is not based on self-monitoring data via remote monitoring technologies |  |
| 31 | Dou K, Yu P, Deng N, Liu F, Guan Y, Li Z, et al. Patients' Acceptance of Smartphone Health Technology for Chronic Disease Management: A Theoretical Model and Empirical Test. Jmir Mhealth and Uhealth. 2017;5(12). doi: 10.2196/mhealth.7886. | **Intervention**; 2b Feedback to the patient is not based on self-monitoring data via remote monitoring technologies |  |
| 32 | Eklind H. A hub clinician's perspective on supported self-care through technology. Primary Health Care. 2017;27(1):20-5. doi: 10.7748/phc.2017.e1147. | **Population/Context**; 1a Doesn't focus or include CVD |  |
| 33 | Eklund C, Eriksson Y, Elfström ML, Söderlund A. Development of an E-health program for self-management of stress related problems. Physiotherapy (United Kingdom). 2015;101:eS348-eS9. doi: 10.1016/j.physio.2015.03.558. | **Intervention**; 2b Feedback to the patient is not based on self-monitoring data via remote monitoring technologies |  |
| 34 | El-Gayar O, Sarnikar S, Wahbeh A, Sprague RH. On the Design of IT-enabled Self-care Systems: A Socio-technical Perspective. 2013:2484-93. doi: 10.1109/hicss.2013.425. | **Population/Context**; 1a Doesn't focus or include CVD |  |
| 35 | Evans J, Papadopoulos A, Silvers C, Charness N, Boot W, Schlachta-Fairchild L, et al. Remote Health Monitoring for Older Adults and Those with Heart Failure: adherence and System Usability2016; 22(6):[480-8 pp.]. Available from: http://cochranelibrary-wiley.com/o/cochrane/clcentral/articles/882/CN-01426882/frame.html. | **Content of interest for the synthesis**; 3a No framework, model or theory reported |  |
| 36 | Finkelstein J, Wood J, Macedo M. DESIGNING PERVASIVE TELEMEDICINE APPLICATIONS USING VARIOUS GAMING PLATFORMS2012. 97-104 p. | **Intervention**; 2b Feedback to the patient is not based on self-monitoring data via remote monitoring technologies |  |
| 37 | Fraiche AM, Eapen ZJ, McClellan MB. Moving Beyond the Walls of the Clinic Opportunities and Challenges to the Future of Telehealth in Heart Failure. Jacc-Heart Failure. 2017;5(4):297-304. doi: 10.1016/j.jchf.2016.11.013. | **Intervention**; 2a Doesn't focus or include self-management as key component |  |
| 38 | Glynn L, Casey M, Walsh J, Hayes PS, Harte RP, Heaney D. Patients' views and experiences of technology based self-management tools for the treatment of hypertension in the community: A qualitative study. BMC Family Practice. 2015;16(1):1-10. doi: 10.1186/s12875-015-0333-7. | **Intervention**; 2b Feedback to the patient is not based on self-monitoring data via remote monitoring technologies |  |
| 39 | Hagglund E, Lynga P, Frie F, Ullman B, Persson H, Melin M, et al. Patient-centred home-based management of heart failure Findings from a randomised clinical trial evaluating a tablet computer for self-care, quality of life and effects on knowledge. Scandinavian Cardiovascular Journal. 2015;49(4):193-9. doi: 10.3109/14017431.2015.1035319. | **Content of interest for the synthesis**; 3a No framework, model or theory reported |  |
| 40 | Hallberg I, Taft C, Ranerup A, Bengtsson U, Hoffmann M, Hofer S, et al. Phases in development of an interactive mobile phone-based system to support self-management of hypertension. Integrated blood pressure control. 2014;7:19-28. doi: 10.2147/ibpc.S59030. | **Intervention**; 2e Self-monitoring is performed only via self-reports |  |
| 41 | Hamper A, Eigner I, Neitzel L, Wickramasinghe N, Bodendorf F, Bentley U, et al. Towards a medical tricorder: Defining medical conditions for consumer self-care with focus on non-invasive technologies. 2017;2017-August. | **Population/Context**; 1a Doesn't focus or include CVD |  |
| 42 | Haywood A, Brownsell S, Hawley MS, Mountain GA, Gelderblom GJ, Soede M, et al. Development of an integrated telehealth system to facilitate self management of long term conditions. 2011;29:1051-7. doi: 10.3233/978-1-60750-814-4-1051. | **Content of interest for the synthesis**; 3c Framework, model or theory is not sufficiently described or not enough information is provided about its operationalization | Authors were contacted to obtain full text but the citation turned out to be a conference presentation abstract. When criteria is applied to the abstract, it can't be clearly assessed in which framework, model or theory the 'user-centred specification' mentioned was based, or how it was operationalized. |
| 43 | Heryana A, Suhardi, Ieee. Smart Personal Health Care Monitoring Services Design using UML2014. 124-30 p. | **Intervention**; 2b Feedback to the patient is not based on self-monitoring data via remote monitoring technologies |  |
| 44 | Holden R. Characterizing the self-care “work” structures and processes of chronic heart failure patients. Clinical and Translational Science. 2013;6(2):118. doi: 10.1111/cts.12047. | **Intervention**; 2b Feedback to the patient is not based on self-monitoring data via remote monitoring technologies |  |
| 45 | Jongstra S, Beishuizen C, Andrieu S, Barbera M, Dorp M, Groep B, et al. Development and Validation of an Interactive Internet Platform for Older People: the Healthy Ageing Through Internet Counselling in the Elderly Study2017; 23(2):[96-104 pp.]. Available from: http://cochranelibrary-wiley.com/o/cochrane/clcentral/articles/643/CN-01426643/frame.html. | **Intervention**; 2b Feedback to the patient is not based on self-monitoring data via remote monitoring technologies |  |
| 46 | Kitsiou S, Thomas M, Marai GE, Maglaveras N, Kondos G, Arena R, et al. Development of an innovative mHealth platform for remote physical activity monitoring and health coaching of cardiac rehabilitation patients2017. 133-6 p. | **Content of interest for the synthesis**; 3c Framework, model or theory is not sufficiently described or not enough information is provided about its operationalization | Refers to user centred design as the development philosophy, and generally mentions several steps taken in the process. However, it is not sufficiently described how it was operationalized and the results section mainly describes the technical features of the technology. |
| 47 | Klein M, Mogles N, van Wissen A. Intelligent mobile support for therapy adherence and behavior change. Journal of Biomedical Informatics. 2014;51:137-51. doi: 10.1016/j.jbi.2014.05.005. | **Population/Context**; 1a Doesn't focus or include CVD |  |
| 48 | Knight EP, Shea K. A Patient-Focused Framework Integrating Self-Management and Informatics. Journal of Nursing Scholarship. 2014;46(2):91-7. doi: 10.1111/jnu.12059. | **Population/Context**; 1a Doesn't focus or include CVD |  |
| 49 | Kotooka N, Asaka M, Sato Y, Kinugasa Y, Nochioka K, Mizuno A, et al. Home telemonitoring study for Japanese patients with heart failure (HOMES-HF): protocol for a multicentre randomised controlled trial2013; 3(6). Available from: http://cochranelibrary-wiley.com/o/cochrane/clcentral/articles/032/CN-00906032/frame.html. | **Intervention**; 2b Feedback to the patient is not based on self-monitoring data via remote monitoring technologies |  |
| 50 | Kuklyte J, Gualano L, Prabhu G, Venkataraman K, Walsh D, Woods C, et al. MedFit: A Mobile Application for Patients in CVD Recovery. Mountain View, California, USA: ACM; 2017. | **Content of interest for the synthesis**; 3a No framework, model or theory reported |  |
| 51 | Lakshminarayan K, Westberg S, Northuis C, Fuller CC, Ikramuddin F, Ezzeddine M, et al. A mHealth-based care model for improving hypertension control in stroke survivors: Pilot RCT. Contemporary clinical trials. 2018;70:24-34. doi: 10.1016/j.cct.2018.05.005. | **Intervention**; 2b Feedback to the patient is not based on self-monitoring data via remote monitoring technologies |  |
| 52 | Lee ML, Dey AK. Real-time feedback for improving medication taking. Toronto, Ontario, Canada: ACM; 2014. | **Population/Context**; 1a Doesn't focus or include CVD |  |
| 53 | Lv N, Lan X, Rosas LG, Simmons ML, Chan A, Entwistle M, et al. Personalized Hypertension Management Using Patient-Generated Health Data Integrated With Electronic Health Records (EMPOWER-H): Six-Month Pre-Post Study. Journal of Medical Internet Research. 2017;19(9):13-. doi: 10.2196/jmir.7831. | **Intervention**; 2d Feedback only provided via remote consultation with HCP |  |
| 54 | Martinez VI, Marquard JL, Saver B, Garber L, Preusse P. Consumer health informatics Interventions must support user workflows, be easy-to-use, and improve cognition: Applying the SEIPS 2.0 model to evaluate patients’ and clinicians’ experiences with the CONDUIT-HID Intervention. International Journal of Human-Computer Interaction. 2017;33(4):333-43. doi: 10.1080/10447318.2016.1278340. | **Population/Context**; 1a Doesn't focus or include CVD |  |
| 55 | Matias N, Sousa MJ, Rocha A, Reis LP, Cota MP, Suarez OS, et al. Mobile Health as a Tool for Behaviour Change in Chronic Disease Prevention A Systematic Literature Review. 2016. | **Population/Context**; 1a Doesn't focus or include CVD |  |
| 56 | Matthew-Maich N, Harris L, Ploeg J, Markle-Reid M, Valaitis R, Ibrahim S, et al. Designing, Implementing, and Evaluating Mobile Health Technologies for Managing Chronic Conditions in Older Adults: A Scoping Review. Jmir Mhealth and Uhealth. 2016;4(2):164-81. doi: 10.2196/mhealth.5127. | **Population/Context**; 1a Doesn't focus or include CVD |  |
| 57 | Mattila J, Ding H, Mattila E, Sarela A, Ieee. Mobile tools for home-based cardiac rehabilitation based on heart rate and movement activity analysis. 2009:6448-+. doi: 10.1109/iembs.2009.5333540. | **Intervention**; 2a Doesn't focus or include self-management as key component |  |
| 58 | [McGillicuddy J, Anderson A, Favela A, Sox L, Wilder S, Brunner-Jackson B, et al. Impact of a patient and provider centered health technology enabled medical regimen self management program among African American uncontrolled hypertensives2015; 77(3):[A57 p.]. Available from: http://cochranelibrary-wiley.com/o/cochrane/clcentral/articles/470/CN-01080470/frame.html.](http://cochranelibrary-wiley.com/o/cochrane/clcentral/articles/470/CN-01080470/frame.html) | **Content of interest for the synthesis**; 3c Framework, model or theory is not sufficiently described or not enough information is provided about its operationalization | Refers to a model and theory used for the development but doesn't provide sufficient detail, citation is a poster abstract. |
| 59 | McGillicuddy J, Sox L, Brunner-Jackson B, Taber D, Mueller M, Chavin K, et al. Sustainability of mobile health medication adherence and blood pressure control program among kidney transplant patients: A one year follow up. Psychosomatic Medicine. 2015;77(3):A89. | **Population/Context**; 1a Doesn't focus or include CVD |  |
| 60 | McGillicuddy JW, Gregoski MJ, Weiland AK, Rock RA, Brunner-Jackson BM, Patel SK, et al. Mobile Health Medication Adherence and Blood Pressure Control in Renal Transplant Recipients: A Proof-of-Concept Randomized Controlled Trial. JMIR research protocols. 2013;2(2):e32-e. doi: 10.2196/resprot.2633. | **Content of interest for the synthesis**; 3c Framework, model or theory is not sufficiently described or not enough information is provided about its operationalization | Refers to 'guidelines for user-centered, iterative based, theory-guided development of empirically validated mHealth programs' but doesn't provide sufficient description of how it was operationalized. Rather focuses on presenting the results of the RCT. |
| 62 | McKay E, Askenas L, Aidemark J, Kommers P, Peng GC. PARTICIPATORY LEARNING: DESIGNING FOR MOBILE ENHANCED SELF-MANAGED HEALTHCARE (MESH)2015. 89-96 p. | **Intervention**; 2b Feedback to the patient is not based on self-monitoring data via remote monitoring technologies |  |
| 61 | McKay E, Askenas L, Aidemark J. Investigating an HCI evaluation criteria framework for patient-centered eHealth solutions a case study of self-management of heart failure. 2014:99-104. doi: 10.1109/IC3e.2014.7081249. | **Content of interest for the synthesis**; 3c Framework, model or theory is not sufficiently described or not enough information is provided about its operationalization | Refers to an evaluation framework that could be of interest. Moreover, it generally refers to eHealth supported **Intervention** on self-management of HF although it doesn't specifically address a **Intervention**/technology under development. Therefore, it is not sufficiently clear how the presented framework can be applied to eHealth development. |
| 63 | Nct. Effects of Telemonitoring on the Outcome of Heart Failure Patients After an Incidence of Acute Decompensation2017. Available from: http://cochranelibrary-wiley.com/o/cochrane/clcentral/articles/981/CN-01583981/frame.html. | **Content of interest for the synthesis**; 3c Framework, model or theory is not sufficiently described or not enough information is provided about its operationalization | Citation is an online protocol that refers to several 'psychological theories' but doesn't appear to be linked to eHealth development, rather just to a specific content part of the **Intervention**. However, in the project's homepage further documentation can be found regarding 'human centred design', with supplementary newsletters and deliverable reports that could be used for the analysis. Citation was be discussed with the research team if this project and its documentation should be included in its present format. It was decided to exclude to maintain cut for published studies. |
| 64 | Nct. Personal Decision Support System for Heart Failure Management2018. Available from: http://cochranelibrary-wiley.com/o/cochrane/clcentral/articles/071/CN-01568071/frame.html. | **Content of interest for the synthesis**; 3a No framework, model or theory reported |  |
| 65 | Neubeck L, Coorey G, Peiris D, Mulley J, Heeley E, Hersch F, et al. Development of an integrated e-health tool for people with, or at high risk of, cardiovascular disease: The Consumer Navigation of Electronic Cardiovascular Tools (CONNECT) web application. International Journal of Medical Informatics. 2016;96:24-37. doi: 10.1016/j.ijmedinf.2016.01.009. | **Intervention**; 2b Feedback to the patient is not based on self-monitoring data via remote monitoring technologies |  |
| 66 | Noergaard B, Sandvei M, Rottmann N, Johannessen H, Wiil U, Schmidt T, et al. Development of a Web-Based Health Care Intervention for Patients With Heart Disease: Lessons Learned From a Participatory Design Study. JMIR research protocols. 2017;6(5). doi: 10.2196/resprot.7084. | **Intervention**; 2b Feedback to the patient is not based on self-monitoring data via remote monitoring technologies |  |
| 67 | Novak LL, Unertl KM, Holden RJ. Realizing the Potential of Patient Engagement: Designing IT to Support Health in Everyday Life. Studies in health technology and informatics. 2016;222:237-47. | **Population/Context**; 1a Doesn't focus or include CVD |  |
| 68 | O'Connor M, Asdornwised U, Dempsey ML, Huffenberger A, Jost S, Flynn D, et al. Using Telehealth to Reduce All-Cause 30-Day Hospital Readmissions among Heart Failure Patients Receiving Skilled Home Health Services. Applied Clinical Informatics. 2016;7(2):238-47. doi: 10.4338/aci-2015-11-soa-0157. | **Intervention**; 2b Feedback to the patient is not based on self-monitoring data via remote monitoring technologies |  |
| 69 | O'Connor S, Hanlon P, O'Donnell CA, Garcia S, Glanville J, Mair FS. Understanding factors affecting patient and public engagement and recruitment to digital health Interventions: a systematic review of qualitative studies. BMC medical informatics and decision making. 2016;16. doi: 10.1186/s12911-016-0359-3. | **Population/Context**; 1a Doesn't focus or include CVD |  |
| 70 | Ozkaynak M, Valdez R, Holden RJ, Weiss J. Infinicare framework for integrated understanding of health-related activities in clinical and daily-living contexts. Health Systems. 2018;7(1):66-78. doi: 10.1080/20476965.2017.1390060. | **Population/Context**; 1a Doesn't focus or include CVD |  |
| 71 | Puijk-Hekman S, van Gaal BGI, Bredie SJH, Nijhuis-van der Sanden MWG, van Dulmen S. Self-Management Support Program for Patients With Cardiovascular Diseases: User-Centered Development of the Tailored, Web-Based Program Vascular View. JMIR research protocols. 2017;6(2). doi: 10.2196/resprot.6352. | **Intervention**; 2b Feedback to the patient is not based on self-monitoring data via remote monitoring technologies |  |
| 72 | Roesler V, Binotto APD, Iochpe C, Palomba EB, Tizatto LAP. Improving Preventive Healthcare with an User-centric Mobile Tele-monitoring Model. Studies in health technology and informatics. 2015;216:648-52. | **Population/Context**; 1a Doesn't focus or include CVD |  |
| 73 | Salisbury C, Thomas C, O'Cathain A, Rogers A, Pope C, Yardley L, et al. TElehealth in CHronic disease: mixed-methods study to develop the TECH conceptual model for Intervention design and evaluation. BMJ open. 2015;5(2). doi: 10.1136/bmjopen-2014-006448. | **Content of interest for the synthesis**; 3b Framework, model or theory is not focused on eHealth design or development | Although the framework referred in the paper is of potential interest, its aim is focused on content and service delivery from the healthcare system perspective (authors also built their model in a comparative manner to the CCM). Although it is proposed to be usable for design and evaluation, it is not the type of model or theory of interest for the review. |
| 74 | Shalan A, Abdulrahman A, Habli I, Tew G, Thompson A. YORwalK: Desiging a Smartphone Exercise Application for People with Intermittent Claudication. Studies in Health Technology & Informatics. 2018;247:311-5. doi: 10.3233/978-1-61499-852-5-311. | **Content of interest for the synthesis**; 3c Framework, model or theory is not sufficiently described or not enough information is provided about its operationalization | The paper refers to the use of a centred design approach but lacks detail in the information that this framework is based on, as well as how it was operationalized. |
| 75 | Siegers K, Mechelen MV, Vanattenhoven J. Mapping the health technology needs of congestive heart failure patients: user needs vs. feasibility. Barcelona, Spain: ACM; 2017. | **Intervention**; 2b Feedback to the patient is not based on self-monitoring data via remote monitoring technologies |  |
| 76 | Suter P, Suter WN, Johnston D. Theory-Based Telehealth and Patient Empowerment. Population Health Management. 2011;14(2):87-92. doi: 10.1089/pop.2010.0013. | **Population/Context**; 1a Doesn't focus or include CVD |  |
| 77 | Triantafyllidis A, Velardo C, Shah SA, Tarassenko L, Chantler T, Paton C, et al. Supporting heart failure patients through personalized mobile health monitoring. 2015:287-90. doi: 10.1109/MOBIHEALTH.2014.7015967. | **Content of interest for the synthesis**; 3c Framework, model or theory is not sufficiently described or not enough information is provided about its operationalization | Refers to an iterative patient-centered approach, but is not sufficiently described. |
| 78 | Varnfield M, Ding H, Karunanithi M. Use of mobile phone based health applications in home care delivery of cardiac rehabilitation. 2012:360-5. doi: 10.2316/P.2012.763-019. | **Content of interest for the synthesis**; 3b Framework, model or theory is not focused on eHealth design or development | Refers to "using the advances in ICT to deliver an alternative and comprehensive home based program [15]" which is the only source mentioned for development, therefore appears to be focused on service and economic analysis rather than eHealth development. |
| 79 | Varnfield M, Karunanithi MK, Särelä A, Garcia E, Fairfull A, Oldenburg BF, et al. Uptake of a technology-assisted home-care cardiac rehabilitation program. Medical Journal of Australia. 2011;194(4):S15-S9. | **Content of interest for the synthesis**; 3a No framework, model or theory reported |  |
| 80 | Vosbergen S, Colkesen EB, Lacroix J, Mosis G, Stappers PJ, Kraaijenhagen RA, et al. Assessment of user needs for self-management services in coronary heart disease: a designerly approach. Delft, Netherlands: ACM; 2010. | **Content of interest for the synthesis**; 3a No framework, model or theory reported |  |
| 81 | Walters DL, Sarela A, Fairfull A, Neighbour K, Cowen C, Stephens B, et al. A mobile phone-based care model for outpatient cardiac rehabilitation: the care assessment platform (CAP). BMC Cardiovascular Disorders. 2010;10:5-. doi: 10.1186/1471-2261-10-5. | **Content of interest for the synthesis**; 3b Framework, model or theory is not focused on eHealth design or development | Refers to a 'Care model' rather than one focused on development of eHealth. |
| 82 | Wheeler T, Abidi S, Splendiani A, Marshall MS, Paschke A, Romano P, et al. Approach to an ontology-based mobile Intervention for patients with hypertension. 2017;2042. | **Intervention**; 2e Self-monitoring is performed only via self-reports |  |
| 83 | White C, Ihegword B, Patterson M, Motz D, Williamson R, Caron JL, et al. Feasibility testing of mhealth for brainhealth: a mobile Intervention to support secondary stroke prevention2016; 47(no pagination). Available from: http://cochranelibrary-wiley.com/o/cochrane/clcentral/articles/401/CN-01139401/frame.html. | **Content of interest for the synthesis**; 3c Framework, model or theory is not sufficiently described or not enough information is provided about its operationalization | Refers to user centred approach but citation is a poster abstract so operationalization cannot be sufficiently assessed. |
| 84 | Woods L, Cummings E, Duff J, Walker K. Design Thinking for mHealth Application Co-Design to Support Heart Failure Self-Management. Studies in health technology and informatics. 2017;241:97-102. | **Intervention**; 2b Feedback to the patient is not based on self-monitoring data via remote monitoring technologies |  |
| 85 | Woods L, Cummings E, Duff J, Walker K. The development and use of personas in a user-centred mHealth design project. Brisbane, Queensland, Australia: ACM; 2017. | **Intervention**; 2b Feedback to the patient is not based on self-monitoring data via remote monitoring technologies |  |
