# Supplementary material for: Supporting Self-Management of Cardiovascular Diseases Through Remote Monitoring Technologies: Metaethnography Review of Frameworks, Models, and Theories Used in Research and Development
Source: J Med Internet Res. 2020 May 21;22(5):e16157. doi: 10.2196/16157 (PMC7273239; doi:10.2196/16157)
Supplement: Multimedia Appendix 4 [file jmir_v22i5e16157_app4.docx]

Multimedia Appendix 4 – Characteristics of included articles

| Year; Author; Country^a^; Project | Journal | Target | Aim | Study design^e^; method(s) |
| --- | --- | --- | --- | --- |
| 2009; Villalba et al [45]; Spain; MyHeart | Conference publication; International Conference on eHealth, Telemedicine, and Social Medicine | HF^b^ | Present iterative validation of user interaction with intervention | Not applicable |
| 2012; McGillicuddy et al [46]; USA; SMASH | Conference publication; Wireless Health | HTN^c^ | Assess patients and HCP^f^ acceptability and adherence to care protocols | Analytic experimental; proof-of-concept RCT^g^ |
| 2014; Bartlett et al [47]; UK; CHF PSMS | BMC Medical Informatics and Decision Making | HF^b^ | Describe system usage and usability | Analytic observational; usability scale, interviews |
| 2015; Rahimi et al [48]; UK; SUPPORT HF | European Heart Journal – Quality of Care and Clinical Outcomes | HF^b^ | Develop system adaptability and evaluate usability | Analytic observational; survey, log data analysis |
| 2015; Triantafyllidis et al [49]; UK; SUPPORT HF | International Journal of Medical Informatics | HF^b^ | Present design and iterative development approach | Not applicable |
| 2016; Chantler et al [50]; UK; SUPPORT HF | Digital Health | HF^b^ | Evaluate usability and interaction with system | Descriptive (qualitative); observations, interviews |
| 2016; Band et al [51]; UK; HOME BP | BMJ Open | HTN^c^ | RCT^g^ protocol to assess feasibility, acceptability, and (cost-)effectiveness | Not applicable |
| 2017; Band et al [52]; UK; HOME BP | Implementation Science | HTN^c^ | Describe intervention planning and development | Not applicable |
| 2017; Bradbury et al [53]; UK; HOME BP | BMC Medical Informatics and Decision Making | HTN^c^ | Examine acceptability and feasibility with HCP^f^ | Descriptive (qualitative); focus groups |
| 2016; Athilingam et al [54]; USA; HeartMapp | Applied Nursing Research | HF^b^ | Assess feasibility and usability with patients and HCP^f^ | Analytic observational; questionnaire and observation |
| 2018a; Athilingam et al [55]; USA; HeartMapp | CIN: Computers, Informatics, Nursing | HF^b^ | Describe intervention mapping and development approach | Not applicable |
| 2018b [56]; Athilingam et al; USA; HeartMapp | Applied Nursing Research | HF^b^ | Explore and test adoption of proof-of-concept app with patients and HCP^f^ | Descriptive (qualitative); Interviews |
| 2017; Srinivas et al [57]; USA; Engage | International Journal Of Human–Computer Interaction | HF^b^ | Describe multi-phased design and development | Analytic observational; observations; interviews, surveys, usability test |
| 2018; Duff et al [58]; Ireland; MedFit App | JMIR Formative Research | CVDs^d^ | Test usability and acceptance with patients | Descriptive (qualitative); Focus group, field trial |
| 2018; Baek et al [59]; South Korea; No project | JMIR Cardio | CVDs^d^ | Test usability and inform development | Analytic observational; survey, interviews, focus group, usability test |
| 2018a; Walsh et al [60]; Ireland, Belgium, Italy, Greece; PATHway | Translational Behavioral Medicine | CVDs^d^ | Describe development, intervention content, and method of delivery | Not applicable |
| 2018b; Walsh et al [61]; Ireland, Belgium; PATHway | Journal of Medical Internet Research | CVDs^d^ | Explore context of implementation with patient and stakeholders | Descriptive (qualitative); interviews |
| ^a^Countries are included according to the reported affiliations of the authors.  ^b^HF: Heart failure.  ^c^HTN: Hypertension.  ^d^CVDs: Cardiovascular diseases (in general).  ^e^Study design classification according to the Oxford Centre for Evidence-Based Medicine [62]. Analytic experimental studies are those in which the researcher manipulates the exposure, allocating subjects to the intervention or exposure group. Analytic observational studies are those in which the researcher simply measures the exposure or treatments of the groups without manipulating the exposure or allocation of subjects. Descriptive (qualitative) studies do not try to quantify the relationship but try to give a picture of what is happening in a population.  ^f^HCP: Health care providers.  ^g^RCT: Randomized controlled trial. | | | | |
