# Supplementary material for: Supporting Self-Management of Cardiovascular Diseases Through Remote Monitoring Technologies: Metaethnography Review of Frameworks, Models, and Theories Used in Research and Development
Source: J Med Internet Res. 2020 May 21;22(5):e16157. doi: 10.2196/16157 (PMC7273239; doi:10.2196/16157)
Supplement: Multimedia Appendix 5 [file jmir_v22i5e16157_app5.docx]

Multimedia Appendix 5 – Quality appraisal of included studies

# Analytic observational studies

|  | **HeartMapp** | **SUPPORT HF** | **Mock-up** | **CHF PSMS** | **Engage** |
| --- | --- | --- | --- | --- | --- |
| *CASP Checklists Item* | Athilingam et al. 2016 | Rahimi et al. 2015 | Baek, et al. 2018 | Bartlett et al. 2014 | Srinivas et al. 2017 |
| **Section A: Are the results of the study valid?** | | | | | |
| Did the study address a clearly focused issue? | Yes | Yes | Yes | Yes | Yes |
| Was the sample recruited in an acceptable way? | Yes | Yes | Yes | No | Can’t tell |
| Was the exposure accurately measured to minimize bias? | Yes | Yes | Yes | Can’t tell | Can’t tell |
| Was the outcome accurately measured to minimize bias? | Yes | Yes | Yes | Yes | Yes |
| Have the authors identified all important confounding factors? | Can’t tell | Can’t tell | Can’t tell | Yes | Yes |
| Have they taken account of the confounding factors in the design and/or analysis? | Can’t tell | Yes | Yes | Yes | Can’t tell |
| Was the follow up of subjects complete enough? | Yes | Yes | No | Can’t tell | Yes |
| Was the follow up of subjects long enough? | Can’t tell | Yes | No | Yes | Yes |
| **Section B: What are the results?** | | | | | |
| Do you believe the results? | Yes | Yes | Yes | Can’t tell | Yes |
| **Section C: Will the results help locally?** | | | | | |
| Can the results be applied to the local population? | Yes | Yes | Yes | No | Can’t tell |
| Do the results of this study fit with other available evidence? | Yes | Yes | Yes | Yes | Yes |

# Descriptive (qualitative) studies

|  | **HeartMapp** | **HOME BP** | **SUPPORT HF** | **PATHway** | **MedFit** |
| --- | --- | --- | --- | --- | --- |
| *CASP Checklists Item* | Athilingam 2018b | Bradbury et al. 2017 | Chantler et al. 2016 | Walsh et al. 2018b | Duff et al. 2018 |
| **Section A: Are the results valid?** | | | | | |
| Was there a clear statement of the aims of the research? | Yes | Yes | Yes | Yes | Yes |
| Is qualitative methodology appropriate? | Yes | Yes | Yes | Yes | Yes |
| Was the qualitative research design appropriate to address the aims of the research? | Yes | Yes | Yes | Yes | Yes |
| Was the recruitment strategy appropriate to the aims of the research? | Yes | Can’t tell | Yes | Yes | Can’t tell |
| Was the data collected in a way that addressed the research issue? | Yes | Yes | Yes | Yes | Yes |
| Has the relationship between researcher and participants been adequately considered? | Can’t tell | Can’t tell | Can’t tell | Can’t tell | Can’t tell |
| **Section B: What are the results?** | | | | | |
| Have ethical issues been taken into consideration? | Yes | Yes | Yes | Can’t tell | Can’t tell |
| Was the data analysis sufficiently rigorous? | Can’t tell | Yes | Yes | Yes | Yes |
| Is there a clear statement of findings? | Yes | Yes | Yes | Yes | Yes |

# Analytic experimental study

|  | **SMASH** |
| --- | --- |
| *CASP Checklists Item* | McGillicuddy et al. 2012 |
| **Section A: Are the results of the trial valid?** | |
| Did the trial address a clearly focused issue? | Can’t tell |
| Was the assignment of patients to treatments randomized? | Yes |
| Were all of the patients who entered the trial properly accounted for at its conclusion? | Can’t tell |
| Were patients, health workers and study personnel ‘blind’ to treatment? | No |
| Were the groups similar at the start of the trial? | Can’t tell |
| Aside from the experimental intervention, were the groups treated equally? | Yes |
| **Section B: Will the results help locally?** | |
| Can the results be applied to the local population, or in your context? | No |
| Were all clinically important outcomes considered? | No |
| Are the benefits worth the harms and costs? | Can’t tell |

# Articles not applicable for appraisal

Athilingam et al. 2018a (HeartMapp)

Band et al. 2016 (HOME BP)

Band et al. 2017 (HOME BP)

Triantafyllidis et al. 2015 (SUPPORT HF)

Walsh et al. 2018a (PATHway)

Villalba et al. 2009(My Heart)
