# Supplementary material for: Supporting Self-Management of Cardiovascular Diseases Through Remote Monitoring Technologies: Metaethnography Review of Frameworks, Models, and Theories Used in Research and Development
Source: J Med Internet Res. 2020 May 21;22(5):e16157. doi: 10.2196/16157 (PMC7273239; doi:10.2196/16157)
Supplement: Multimedia Appendix 6 [file jmir_v22i5e16157_app6.docx]

Multimedia Appendix 6 – List of identified and translated metaphors

Contents

[Highlights of identification and translation of key metaphors 2](#_Toc16603634)

[List of key metaphors 3](#_Toc16603635)

[Sources and abbreviations 3](#_Toc16603636)

[Interpretive codes 4](#_Toc16603637)

[Key metaphors and definitions per study 5](#_Toc16603638)

[Translation of key metaphors 19](#_Toc16603639)

[Clusters of key metaphors 19](#_Toc16603640)

[Idiomatic reciprocal and refutational translations 27](#_Toc16603641)

#

# Highlights of identification and translation of key metaphors

**In total, 333 key metaphors were initially identified across all studies.** These were reduced to 293 after merging repeated cases across studies (eg, the planning stage from the Person-Based Approach was operationalized by different studies and projects). 190 out of 293 (65%) were primary key metaphors derived from the identified frameworks, models, and theories. 103 out of 293 (35%) were other meaningful phrases, concepts, ideas or perspectives of authors from included studies.

**The collective characterization process assigned metaphors to at least one of eight possible clusters (5 assumed holistic principles and 3 categories of effectiveness).** From largest to smallest: 33 out of 293 (11%) metaphors were characterized under the principle that *eHealth development is a participatory development process*; 30 out of 293 (10%) metaphors were related to the principle that *eHealth development requires continuous evaluation cycles*; 18 out of 293 (6%) metaphors were clustered under the principle that *eHealth development is intertwined with implementation*; 15 out of 293 (5%) metaphors were grouped under the principle that *eHealth development creates new infrastructures for improving health care, health, and well-being*; lastly, 8 out of 293 (3%) metaphors were related to the principle that *eHealth development is coupled with persuasive design*. In the characterization for effectiveness: 48 out of 293 (16%) metaphors were clustered as key ingredients for *technology adoption*; 46 out of 293 (16%) metaphors were clustered as key ingredients for *behavior change*; and 35 out of 293 (12%) metaphors were clustered as key *health-related* *outcomes* of an intervention.

**Through idiomatic translation, 13 metaphors were found to embody the meaning of other concepts and themes (reciprocal translation).** For example, the concepts of *user involvement* and *target population involvement* were reciprocal metaphors. Likewise, the meaning of the metaphor of *development and evaluation of acceptability and feasibility* could integrate the meaning of the *pretesting* and *evaluate* concepts. On the other hand, the *parallel approach* and *stepwise approach* metaphors were considered refutational.

# List of key metaphors

## Sources and abbreviations

| Abbreviation | Description | Abbreviation | Description |
| --- | --- | --- | --- |
| *5E* | 5E Usability Approach | *MedFit* | Overarching project |
| *BCW* | Behavior Change Wheel | *MFPE* | Multidimensional Framework for Patient and family Engagement |
| *BMC* | Business Model Canvas | *MRC* | Medical Research Council’s (MRC) Guidance for Developing and Evaluating Complex Interventions |
| *CARE* | Congratulate, Ask, Reassure, Encourage Approach | *NPT* | Normalization Process Theory |
| *CHF PSMS* | Overarching project: Congestive Heart Failure Personalized Self-Management System | *PATHway* | Overarching project: Physical Activity Towards Health |
| *COM-B* | Capability, Opportunity, Motivation-Behavior model | *PBA* | Person Based Approach |
| *CSM* | Common-Sense Model of Self-Regulation | *PWL* | Patient Work Lens |
| *CT* | Control Theory | *REF* | Realistic Evaluation Framework |
| *DEP* | Development and Evaluation Process for mHealth | *SCT* | Social Cognitive Theory |
| *DTT* | Domestication of Technology Theory | *SDT* | Self-Determination Theory |
| *HOME BP* | Overarching project: Home and Online Management and Evaluation of Blood Pressure | *SEIPS 2.0* | Systems Engineering Initiative for Patient Safety 2.0 |
| *HPI* | Holistic Patient Interaction model | *SEM* | Social Ecological Model |
| *IDM* | Iterative Design Model | *TAM* | Technology Acceptance Models |
| *IM* | Intervention Mapping | *UCD (Ad hoc)* | User-Centred Design (Ad hoc approach) |
| *IMB* | Information, Motivation, Behavioral skills model | *UCD (Monk)* | User-Centred Design (Monk 2000) |
| *IRA* | Iterative Refinement Approach | *UCD-CHIT* | User-Centred Design of Consumer-facing Health IT |
| *ISD* | Iterative Software Design process | *UF* | Usability Framework |

## Interpretive codes

These themes were open coded by the main reviewer during data extraction in ATLAS.ti. Below are their working definitions.

| Theme | Definition | Theme | Definition |
| --- | --- | --- | --- |
| *Development assumption/*  *Development state-of-the-art* | Remarkable idea or phrase by the authors of a study regarding eHealth development, considered ‘state-of-the-art’ if a clear reference is provided, or the own authors ‘assumption’ if not. | *Practical applications* | Practical applications are the translations of theoretical methods of behavior change to practical intervention elements. Applications are by definition specific, ideally tailored to populations, intervention contexts and behavioral domains. |
| *eHealth added value* | Remarkable idea or phrase by the authors of a study regarding the added value of eHealth interventions. | *Parameters of effectiveness* | Parameters for effectiveness are the characteristics that a practical application must manifest for it to accurately reflect the theoretical method. When these parameters are lost in translation from method to application, effective behavior change is undermined and may even result in counterproductive effects. Evidence for the existence of such parameters can range from theoretical to meta-analytical. |
| *eHealth state-of-the-art* | Remarkable idea or phrase by the authors of a study regarding eHealth in general and backed up by a clear reference. | *Self-management assumption/*  *Self-management state-of-the-art* | Remarkable idea or phrase by the authors of a study regarding the operationalization of self-management, considered ‘state-of-the-art’ if a clear reference is provided, or the own authors ‘assumption’ if not. |
| *Evidence gap* | Remarkable idea or phrase by the authors of a study regarding an evidence gap in eHealth research and development. | *Technology acceptance* | Remarkable idea or phrase by the authors of a study regarding technology acceptance in eHealth interventions. |
| *Heterogeneity* | Remarkable idea or phrase by the authors of a study regarding differences in patients’ characteristics, and how to address these. It could be condition-specific. | *Framework/theory/model*  *state-of-the-art* | Remarkable idea or phrase by the authors of a study regarding a specific framework, model, or theory and backed up by a clear reference. |
| *Limitations of study/project* | Remarkable idea or phrase by the authors of a study regarding limitations of eHealth research and development. |  |  |

## Key metaphors and definitions per study

| # | Source | Key metaphor | Interpretive definition |
| --- | --- | --- | --- |
|  | **Athilingam et al. 2016** | | |
| 1 | HeartMapp | CHF info/knowledge | *Practical application*; Enabling patients to read and listen to CHF educational information on daily self-management, medications, diet, and physical activity, managing other chronic diseases or conditions, and managing feelings (emotions) about CHF. |
| 2 | HeartMapp | Personal Health Buddy/Coach | *Practical application*; A personal health buddy coach that serves as a conduit to empower patients through daily reminders and feedback; including features promoting (self-)assessment, monitoring exercises, vital signs, and summary stats |
| 3 | HeartMapp | Adherence to medication | Health outcomes |
| 4 | HeartMapp | Diet | Health outcomes |
| 5 | HeartMapp | Improved self-care skills | Health outcomes |
| 6 | HeartMapp | Physical activity | Health outcomes |
| 7 | IMB | Behavioral skills | *Parameters of effectiveness*; The adoption or acting out of healthy behavior skills that result in desired behavior change |
| 8 | IMB | Information | *Parameters of effectiveness*; Information about ones’ health and/or ways to change unhealthy behavior |
| 9 | IMB | Motivation (IMB) | *Parameters of effectiveness*; Personal or intrinsic motivation and social motivation from support of family and friends to change the unhealthy behavior |
| 10 | MFPE | Patient engagement | *Parameters of effectiveness*; Key mediator for behavior change in patients with CHF |
|  | **Athilingam et al. 2018a** | | |
| 11 | HeartMapp | Assessment | *Practical application*s; This feature includes daily patient alerts to check weight and complete a symptom questionnaire. |
| 12 | HeartMapp | Education (Practical application) | *Practical application*s; This feature provides users with interactive, audio-enabled educational information and reference materials |
| 13 | HeartMapp | Exercises and feedback | *Practical application*s; This feature includes an animated deep breathing exercise and measures physical activity using distance walked. |
| 14 | HeartMapp | Global health | Health outcomes |
| 15 | HeartMapp | HF knowledge | Health outcomes |
| 16 | HeartMapp | HF self-management | Health outcomes |
| 17 | HeartMapp | Adherence to medication | Health outcomes |
| 18 | HeartMapp | Medication list and reminder alerts | *Practical application*s; This feature includes an editable personalized medication list that reminds patients of their medication schedule and demands an entry to indicate medication taken or reason for missing the medication. |
| 19 | HeartMapp | Quality of life | Health outcomes |
| 20 | HeartMapp | Real-time vital signs monitoring | *Practical application*s; Tracking continuous heart rate, distance walked, and sleep quality. |
| 21 | HeartMapp | Reduce readmissions | Health outcomes |
| 22 | HeartMapp | Stats | *Practical application*s; This feature is a graphic module displaying patient performance trends in weight, blood pressure, symptoms, exercise, and vital signs |
| 23 | IM | Proximal program objective(s) | Stage of intervention mapping that provides the foundation for the intervention by specifying who and what will change as result of the intervention. |
| 24 | IM | Selection of theory-based methods | Stage of intervention mapping that produces the intervention methods and strategies that match the proximal program objectives. |
| 25 | IM | Program plan | Stage of intervention mapping that includes a description of the scope and sequence of the components of the intervention, the completion of program materials, and protocols for implementation. |
| 26 | IM | Needs assessment | Stage of intervention mapping that entails a systematic study of quality of life and health status and those factors that influence them, such as health behavior and environment. |
| 27 | IM | Adaptation and implementation plan | Stage of intervention mapping that includes consideration of program sustainability. Matrices are developed with adoption and implementation objectives juxtaposed to personal and external determinants. |
| 28 | IM | Evaluation plan | Stage of intervention mapping where variables are defined in a measurable way regarding the decisions about learning and change objectives, methods, strategies, and implementation. |
| 29 | IMB | Behavioral skills | *Parameters of effectiveness*; It includes ensuring that the patient has the specific behavioral tools or strategies necessary to perform behaviors such as daily weighing, symptom assessment, and exercise. |
| 30 | IMB | Information | *Parameters of effectiveness*; It is the basic knowledge about a medical condition that might include how it develops, its expected course, and effective strategies for its management. |
| 31 | IMB | Motivation (IMB) | *Parameters of effectiveness*; It encompasses intrinsic motivation of personal attitudes toward adherence to the behavior and perceived social support for such behavior. |
| 32 | MFPE | Patient engagement | *Parameters of effectiveness*; Defined as patients, families, their representatives, and health professionals working in active partnership at various levels across the health care system—direct care, organizational design and governance, and policy making—to improve health and health care. |
|  | **Athilingam et al. 2018b** | | |
| 33 | BMC | Channels | Key insights for building a minimum viable product; The communication and distribution channels to reach clients and offer them the value proposition |
| 34 | BMC | Cost structure | Key insights for building a minimum viable product; The cost structure resulting from the business model |
| 35 | BMC | Customer relationships | Key insights for building a minimum viable product; The relationships established with clients |
| 36 | BMC | Customer segments | Key insights for building a minimum viable product; The segment(s) of clients that are addressed by the value proposition |
| 37 | BMC | Key activities | Key insights for building a minimum viable product; The key activities necessary to implement the business model |
| 38 | BMC | Key partners | Key insights for building a minimum viable product; The key partners and their motivations to participate in the business model |
| 39 | BMC | Key resources | Key insights for building a minimum viable product; The key resources needed to make the business model possible |
| 40 | BMC | Revenue streams | Key insights for building a minimum viable product; The revenue streams generated by the business model (constituting the revenue model) |
| 41 | BMC | Value propositions | Key insights for building a minimum viable product; The value proposition of what is offered to the market |
| 42 | Authors | Patient activation | *Self-management state-of-the-art*; The term ‘patient activation’ is employed to represent patients who are ‘more eﬀective managers of their health and healthcare’ (Lupton, 2013). However, even these digitally engaged, tech savvy patients may ﬁnd the obligation of self-care surveillance overwhelming, forcing them to confront their illness, engage in routine actions they would rather avoid, or deal with digital interactions that are tiresome (Oudshoorn, 2012). |
|  | **Baek et al. 2018** | | |
| 43 | Authors | Bidirectional service model | *Development assumption*; Considering the patients’ perspective, advice from doctors or communication with them is proposed as an important factor in a successful eHealth support model |
| 44 | UCD (Ad hoc) | Focus group interview with HCP | Qualitative data collection technique used to obtain detailed information on the thoughts, emotions, attitudes, or experiences of participants |
| 45 | UCD (Ad hoc) | Surveys and interviews with patients | User research can be conducted through face-to-face interviews and surveys |
| 46 | UCD (Ad hoc) | Mock-up design | Mock-ups are visualizations that enable users to experience the functions that will be implemented in a technology |
| 47 | UCD (Ad hoc) | Usability test | Method to evaluate how easily the end user understands, learns, and uses software or an app under specific conditions |
|  | **Band et al. 2016** | | |
| 48 | Authors | Tailored, personalized, and timely support | *eHealth added value*; *eHealth state-of-the-art*; Increasingly widespread access to the internet and mobile phones means that eHealth can be accessible to the majority of patients and can be used to provide information and support at any time the patient needs it. eHealth can empower patients by providing better access to personalized information and support for active involvement in treatment and self-management |
|  | **Band et al. 2017** | | |
| 49 | BCW/COM-B | Automatic Motivation | *Parameters of effectiveness*; Target constructs (sources of behaviors); Automatic processes involving emotional reactions, desires (wants and needs), impulses, inhibitions, drive states and reflex responses |
| 50 | BCW/COM-B | Physical Opportunity | *Parameters of effectiveness*; Target constructs (sources of behaviors); Physical skill, strength or stamina |
| 51 | BCW/COM-B | Psychological Capability | *Parameters of effectiveness*; Target constructs (sources of behaviors); Knowledge or psychological skills, strength or stamina to engage in the necessary mental processes |
| 52 | BCW/COM-B | Reflective Motivation | *Parameters of effectiveness*; Target constructs (sources of behaviors); Reflective processes involving plans (self-conscious intentions) and evaluations (beliefs about what is good and bad) |
| 53 | BCW/COM-B | Social Opportunity | *Parameters of effectiveness*; Target constructs (sources of behaviors); Opportunity afforded by interpersonal influences, social cues and cultural norms that influence the way that we think about things., e.g., the words and concepts that make up our language |
| 54 | BCW/COM-B | Education (Intervention function) | Intervention functions |
| 55 | BCW/COM-B | Enablement | Intervention functions |
| 56 | BCW/COM-B | Environmental Restructuring | Intervention functions |
| 57 | BCW/COM-B | Persuasion | Intervention functions |
| 58 | BCW/COM-B | Training | Intervention functions |
| 59 | CSM | Illness beliefs | *Parameters of effectiveness*; Proposed mediating variables (Logic model) |
| 60 | CSM | Treatment beliefs | *Parameters of effectiveness*; Proposed mediating variables (Logic model); Outcome expectancy is defined as a person’s estimate that a given behavior will lead to certain outcomes (equivalent to SCT’s ‘outcome expectancy’) |
| 61 | Authors | Integrating theory-, evidence- and person based approaches | *Approach to eHealth*; A combination of theory-, evidence- and person based approaches are important to increase the acceptability, engagement with, and effectiveness of an intervention. |
| 62 | Authors | Theoretical modelling | *Development state-of-the-art*; Theoretical modelling is vital in success complex intervention development, in order to identify and change the determinants of behavior. |
| 63 | HOME BP | Automated BP feedback | *Practical application*s |
| 64 | HOME BP | Automated e-mails prompts/reminders | *Practical application*s |
| 65 | HOME BP | Behavioral support provision | Health professional intervention components |
| 66 | HOME BP | Enacting medication titration procedures | Health professional intervention components |
| 67 | HOME BP | HCP: Pre-planned medication titration schedule | *Practical application*s |
| 68 | HOME BP | HCP: Support for lifestyle changes & BP monitoring | *Practical application*s |
| 69 | HOME BP | Health professional engagement with the intervention | Health professional intervention components |
| 70 | HOME BP | Patient engagement with the intervention | Patient intervention components |
| 71 | HOME BP | Patient home blood pressure self-monitoring | Patient intervention components |
| 72 | HOME BP | Patient lifestyle change | Patient intervention components |
| 73 | HOME BP | Patient medication adherence and titration | Patient intervention components |
| 74 | HOME BP | Patient: Self-monitoring & BP entry | *Practical application*s |
| 75 | HOME BP | Patient: Self-selected lifestyle changes | *Practical application*s |
| 76 | HOME BP | Website sessions (Patient & HCP) | *Practical application*s |
| 77 | MRC | Modelling process and outcomes | To provide a diagram (logic model) representing the hypothesised causal relationships mediating intervention outcomes |
| 78 | NPT | CA: Contextual integration | Target constructs (Logic model); Having the right social and financial resources; integration of the illness in to social situation |
| 79 | NPT | CA: Interactional workability | Target constructs (Logic model); Taking medications, medication side-effects, engaging with DI, lifestyle changes – integrating all of these in to everyday life. |
| 80 | NPT | CA: Relational integration | Target constructs (Logic model); Developing relationships with HCPs; Building confidence in the system and in relationships with HCPs |
| 81 | NPT | CA: Skillset workability | Target constructs (Logic model); Developing the skills and routine to self-monitor |
| 82 | NPT | CH: Communal specification | Target constructs (Logic model); Gaining info about the condition and its management with the help of others; Discussing or altering current management plans with others (family, HCPs) |
| 83 | NPT | CH: Differentiation | Target constructs (Logic model); Understanding and differentiating between aspects of the illness & treatment – for example, when titration is necessary and when it is not |
| 84 | NPT | CH: Individual specification | Target constructs (Logic model); Gathering own info about condition, or developing own understanding; Patient chooses to continue with self-monitoring or treatment escalation regime |
| 85 | NPT | CH: Internalization | Understanding the implications of treatment and when titration is necessary |
| 86 | NPT | Cognitive participation (CP) | Target constructs (Logic model) |
| 87 | NPT | Coherence (CH) | Target constructs (Logic model) |
| 88 | NPT | Collective action (CA) | Target constructs (Logic model) |
| 89 | NPT | CP: Activation | Arranging help from HCPs or others |
| 90 | NPT | CP: Enrolment | Target constructs (Logic model); Engaging with others (family, HCP) to enable them to support BP management |
| 91 | NPT | CP: Initiation | Target constructs (Logic model); Organizational aspects of behavior involved in management of blood pressure |
| 92 | NPT | CP: Legitimation | Target constructs (Logic model); Seeking reassurance from others about appropriateness of management plans |
| 93 | NPT | Reflexive monitoring (RM) | Target constructs (Logic model) |
| 94 | NPT | RM: Communal appraisal | Discussing or altering current management plans with others (family, HCPs) |
| 95 | NPT | RM: Individual appraisal | Patient chooses to continue with self-monitoring or treatment escalation regime |
| 96 | NPT | RM: Reconfiguration | Target constructs (Logic model); Altering a set routine (such as medication escalation) when required |
| 97 | NPT | RM: Systematization | Ways to keep up with newly available treatments |
| 98 | PBA | Design (PBA) | Creation of guiding principles to help developers summarize and easily refer to features of the intervention identified as central to achieving the intervention objectives. This stage can be operationalized, for example, through theoretical modelling via a theory-based behavioral analysis or the creation of a logic model. |
| 99 | PBA | Development and evaluation of acceptability and feasibility | All intervention components evaluated in detail and optimized from user perspective. This stage can be operationalized, for example, by collating and analyzing evidence derived from primary mixed- methods research such as a feasibility study. |
| 100 | PBA | Planning | Identification of key behavioral issues, needs, and challenges the intervention must address. This stage can be operationalized, for example, through collating and analyzing evidence via a quantitative or qualitative literature synthesis, as well as through primary qualitative research. |
| 101 | SCT | Outcome expectancy | *Parameters of effectiveness*; Proposed mediating variables (Logic model); Outcome expectancy is defined as a person’s estimate that a given behavior will lead to certain outcomes. |
| 102 | SCT | Self-efficacy | *Parameters of effectiveness*; Proposed mediating variables (Logic model); The expectation that one can successfully execute the behavior required to produce the outcomes. |
|  | **Bartlett et al. 2014** | | |
| 103 | CHF PSMS | Ability to walk further, lose weight or meet personal goals | Outcomes (CMO hypotheses); This outcome can result from the combination of the patient's activation (C) and the provision of a walking intervention (e.g., via a mobile device) (M) |
| 104 | CHF PSMS | Acceptability and fit of hardware in everyday life | Mechanisms (CMO hypotheses) |
| 105 | CHF PSMS | Access to technical support | Context (CMO hypotheses) |
| 106 | CHF PSMS | All patients are able to use the system and continue to use it for the duration of the evaluation | Outcomes (CMO hypotheses); This outcome can result from a combination of the eHealth literacy of the users (C) and the application of a user-centred design process (M) |
| 107 | CHF PSMS | Balance between activity and rest | Outcomes (CMO hypotheses); This outcome can result from the combination of the over activity/ rest cycle of the patient (C) and the provision of feedback on activity (M) |
| 108 | CHF PSMS | Behavior change that is sustainable over the long term | Outcomes (CMO hypotheses); This outcome can result from the combination of the patient's engagement with a variety of lifestyle changes (C) and the provision of behavioral change techniques such as self-monitoring of symptoms, goal setting according to the user's lifestyle, or feedback on activity performance (M) |
| 109 | CHF PSMS | Co-morbidities | Context (CMO hypotheses); Patients might have more than one chronic condition |
| 110 | CHF PSMS | Condition stability | Context (CMO hypotheses) |
| 111 | CHF PSMS | Consideration of physical and psychological barriers to lifestyle changes | *Parameters of effectiveness*; Mechanisms (CMO hypotheses); Such as increased pain or discomfort while walking due to co-morbidities |
| 112 | CHF PSMS | Continued engagement with system | Outcomes (CMO hypotheses); This outcome can result from the combination of an acceptable System Usability Scale score (C) and a lack of critical problems with the technology (M) |
| 113 | CHF PSMS | eHealth literacy | Context (CMO hypotheses) |
| 114 | CHF PSMS | Engagement and happiness to use the system | Outcomes (CMO hypotheses); This outcome can result from the combination of usage of technology in a home setting (C) and the acceptability and fit of the hardware in every day life (M) |
| 115 | CHF PSMS | Engagement with variety of lifestyle changes | Context (CMO hypotheses) |
| 116 | CHF PSMS | Feedback on activity | *Practical application*s; Mechanisms (CMO hypotheses); My review [Touch screen computer]; Review daily walk as ‘too hard’, ‘just right’, or ‘too easy’; Adjust tomorrow’s walking |
| 117 | CHF PSMS | Feedback on activity performance | *Practical application*s; Mechanisms (CMO hypotheses) |
| 118 | CHF PSMS | Goal setting related to user’s lifestyle | *Practical application*s; Mechanisms (CMO hypotheses) |
| 119 | CHF PSMS | Improved symptom control, ideally reducing need for health professional involvement. | Outcomes (CMO hypotheses); This outcome can result from the combination of the patient's condition stability (C) and the provision of self-monitoring and tailored feedback (M) |
| 120 | CHF PSMS | Increased levels of knowledge about self-management. | Outcomes (CMO hypotheses); This outcome can result from the combination of the patient's current level of knowledge (C) and the provision of an information and advice section (M) |
| 121 | CHF PSMS | Information and advice section | *Practical application*s; Mechanisms (CMO hypotheses); [Touch screen computer]; View heart failure related information; Complete quizzes to test knowledge |
| 122 | CHF PSMS | Knowledge about self-management | Context (CMO hypotheses) |
| 123 | CHF PSMS | Lack of critical problems with technology | Mechanisms (CMO hypotheses) |
| 124 | CHF PSMS | Over activity / rest cycle | Context (CMO hypotheses) |
| 125 | CHF PSMS | Patient activation (CMO hypothesis) | Context (CMO hypotheses) |
| 126 | CHF PSMS | Self-management tailored to patient’s condition | Outcomes (CMO hypotheses); This outcome can result from the combination of the patient's co-morbidities (C) and the consideration of physical and psychological barriers to lifestyle changes (M) |
| 127 | CHF PSMS | Self-monitoring and tailored feedback provision | *Practical application*s; Mechanisms (CMO hypotheses); My daily plan [Touch screen computer]; Input blood pressure and weight measurements; Answer symptom questions; Produce a daily plan of activities; My progress [Touch screen computer]; Get graphical feedback on weight, blood pressure, symptoms and activity levels; My maps and travel [Touch screen computer]; Review a map of the day’s walk using GPS |
| 128 | CHF PSMS | Self-monitoring of symptoms | *Practical application*s; Mechanisms (CMO hypotheses) |
| 129 | CHF PSMS | Technology usage in a home setting | Context (CMO hypotheses) |
| 130 | CHF PSMS | User-centred design process undertaken | Mechanisms (CMO hypotheses) |
| 131 | CHF PSMS | Walking intervention | *Practical application*s; Mechanisms (CMO hypotheses); [Mobile device] |
| 132 | CT | Self-monitoring | *Practical application*s; Self-monitoring is a theory-based technique. An intervention designer could hypothesize that including self-monitoring (theory-based mechanism) in their intervention will increase physical activity (outcome). |
| 133 | Authors | Meaningful feedback | *Self-management assumption*; For technology to support self-management, the information gathered by the technology must be fed back to the patient in a way that increases understanding and empowerment |
| 134 | Authors | Measuring changes in knowledge | Quantifying knowledge (e.g., about self-management) and ensuring that the change observed is neither random nor the result of measurement error is challenging, especially with a small sample size. This can be overcome, for example, via the TELER method. |
| 135 | MRC | Development phase | Identifying the evidence base, Identifying/developing theory, Modelling process and outcomes |
| 136 | REF | Formulate the initial CMO hypotheses | Formulating specific CMO hypotheses This stage can, for example, be operationalized through literature reviews with a multidisciplinary range. |
| 137 | REF | Analyse data in terms of CMO hypotheses | Through analysis of data the realist evaluators aim to understand the effects on outcomes and identify what works, for whom, in what circumstances [13] |
| 138 | REF | CMO hypotheses | The outcomes (O) that result from using an intervention result from a combination of the context (C) that it is used in, and the mechanisms (M) that form the intervention. The detailed hypotheses formed are referred to as CMO hypotheses. |
| 139 | REF | Collect mixed methods data | Collecting a range of data to evaluate the proposed CMO hypotheses. This stage can be operationalized, for example, by collecting tracking data, use of weight and blood pressure monitors, or usability and knowledge scored |
| 140 | REF | Design a field study to evaluate CMO hypotheses | In order to test and generate revised CMO hypotheses, the technology can be deployed in a realistic setting. |
| 141 | REF | Synthesis of new data into refine CMO hypotheses | The CMO hypotheses are revised, rejected, or new ones are proposed according to the analysis of the data |
| 142 | UCD (Monk) | Testing a top level design against your understanding of the work | Common processes in user centred design; Testing the design using story boards, scenario or cognitive walkthrough. |
| 143 | UCD (Monk) | Understanding the work | Common processes in user centred design; Understanding the particular work to be supported by the eHealth system. As with the work context, the data used to do this will come from interviews and observation in the work place. Common types of representations for work are Hierarchical Task Analysis and scenarios |
| 144 | UCD (Monk) | Understanding the work context | Common processes in user centred design; Understanding the work context involves identifying all the stakeholders and their concerns. eHealth changes the way people work, otherwise there would be no point in introducing them |
| 145 | UCD (Monk) | User testing of more detailed prototypes | Common processes in user centred design; Testing design using paper prototypes or simulations |
|  | **Bradbury et al. 2017** | | |
| 146 | CARE | Ask | Guidelines for patient-centred care to support online intervention (Blended care); Asking the patients how they are getting on, ask if they have any questions or concerns. |
| 147 | CARE | Congratulate | Guidelines for patient-centred care to support online intervention (Blended care); Congratulating the patients on anything they did well |
| 148 | CARE | Encourage | Guidelines for patient-centred care to support online intervention (Blended care); Encouraging the patients to keep monitoring their blood pressure, entering their blood pressure readings into HOME BP. |
| 149 | CARE | Reassure | Guidelines for patient-centred care to support online intervention (Blended care); Reassuring the patients about any concerns they have. |
| 150 | HOME BP | Clinical inertia | Key outcome of HOME BP model; The failure to establish appropriate targets and escalate treatment to achieve treatment goals. |
| 151 | PBA | Development and evaluation of acceptability and feasibility | All intervention components evaluated in detail and optimized from user perspective. This stage can be operationalized, for example, by collating and analyzing evidence derived from primary mixed- methods research such as a feasibility study. |
| 152 | PBA | Planning | Identification of key behavioral issues, needs, and challenges the intervention must address. This stage can be operationalized, for example, through collating and analyzing evidence via a quantitative or qualitative literature synthesis, as well as through primary qualitative research. |
| 153 | SCT | Outcome expectancy | *Parameters of effectiveness*; A judgement of the likely consequence a behavior will produce. |
| 154 | SCT | Self-efficacy | *Parameters of effectiveness*; People’s judgements of their capabilities to organize and execute courses of action required to attain designated types of performances. It is concerned not with the skills one has but with judgments of what one can do with whatever skills one possesses. |
| 155 | SDT | Autonomous motivation | Intrinsic motivation, the doing of an activity for its inherent satisfactions. It is highly autonomous and represents the prototypic instance of self-determination. |
| 156 | SDT | Need for autonomy | Psychological need to perceive that they have choices and that they can self-determine what to do. |
| 157 | SDT | Need for competence | Psychological need to exert a meaningful effect on one's environment. |
| 158 | SDT | Need for relatedness | Psychological need to have a sense of belonging and connectedness with others. |
|  | **Chantler et al. 2016** | | |
| 159 | Authors | Diversity of user experiences | *Development assumption*; *Heterogeneity (Heart Failure)*; *Technology acceptance*; Accounting for the diversity of user experiences in the development and maintenance of eHealth systems is likely to increase the extent of successful uptake and impacts on outcomes for patients and providers. In practice, this highlights the importance of investing time into understanding how diﬀerent types of users adopt and incorporate digital-health technology into their daily lives. |
| 160 | Authors | Connection | *Development assumption*; *Heterogeneity (Heart Failure)*; A key motivator was the sense of connection an eHealth system provides to a support team (e.g., research group), outweighing, for instance, the ability to view daily readings or health information |
| 161 | Authors | Personalization and tailoring | *Development assumption*; *Heterogeneity (Heart Failure)*; Users might be able to use the system with diﬀerent levels of involvement and understanding, some taking active control (engagement) of their health whereas others appreciate using the system more passively. This could correlate with their level of digital competency (literacy), which makes personalization desirable |
| 162 | Authors | Real-time feedback in self-care | *eHealth added value*; *eHealth state-of-the-art*; *Self-management state-of-the-art*; Real-time feedback enhances self-care by providing patients with timely instructions and support. Harnessing the value of these ‘teachable moments’ is critical and should be part of well-designed and supported eHealth systems to improve patients’ experience of living with their condition. |
| 163 | Authors | Routine | *Development assumption*; *Heterogeneity (Heart Failure)*; Continuous usage can be strongly associated with patients ﬁtting the self-monitoring of a condition into their daily routine |
| 164 | DTT | Appropriation | Dimensions of domestication of technology; Dimension that addresses questions such as why users are interested in using the system and what motivates them. This can include an interest to take part in an eHealth related study |
| 165 | DTT | Conversion | Dimensions of domestication of technology; Dimension that deals with the expressed aspirations and continuing interest of users in using an eHealth system |
| 166 | DTT | Domestication of technology | The processes of acceptance, rejection and use of technology by its users. Users are seen as social entities and this conceptual model aims to provide a framework for understanding how technology innovations change, and are changed, by their social contexts. For instance, according to the Domestication of Technology Theory this process can be divided in four dimensions: appropriation, objectification, incorporation, and conversion. |
| 167 | DTT | Incorporation | Dimensions of domestication of technology; Dimension that is about the practical hands-on use of the eHealth system by users, and how they continue to evaluate its usefulness becomes more or less integral to their daily lives |
| 168 | DTT | Objectification | Dimensions of domestication of technology; Dimension that is about determining what the eHealth system will be used for by the users |
| 169 | MRC | Exploratory or pilot trial | A phase that focuses on optimizing trial measures before clinical evaluation of an intervention |
| 170 | MRC | Modelling (MRC) | A phase that focuses on identifying answer to the question: ‘How does the intervention work?’ |
| 171 | MRC | Parallel approach | A parallel approach is proposed in practice by the MRC framework for the development and evaluation of complex interventions. This means combining phases 0-II of their framework into one larger activity to develop understanding of the problem, the intervention, and the evaluation |
| 172 | MRC | Preclinical or theoretical | A phase that focuses on identifying answer to the question: ‘Why should this intervention work?’ |
| 173 | MRC | Stepwise approach | A stepwise approach is proposed by the MRC framework for the development and evaluation of complex interventions: 0—Preclinical or theoretical (why should this intervention work?); 1—Modelling (how does it work?); 2—Exploratory or pilot trial (optimizing trial measures); 3—Definitive randomized controlled trial; 4—Implementation |
| 174 | MRC | User involvement | End-users should be involved in assessing the acceptability and usability of a behavioral intervention before scaling it up for further clinical evaluation. This can be done in practice through a multi-phased parallel approach of theoretical, modelling, and exploratory or piloting phases (for example as proposed by the MRC) |
| 175 | UF | Ethnography | An indirect testing method to test usability. |
|  | **Duff et al. 2018** | | |
| 176 | BCW | Education (Intervention function) | Intervention functions; Increasing knowledge or understanding |
| 177 | BCW | Enablement | Intervention functions; Increasing means/reducing barriers to increase capability |
| 178 | BCW | Environmental restructuring | Intervention functions; Changing the physical or social context |
| 179 | BCW | Modelling (BCW) | Intervention functions; Providing an example for people to aspire to or imitate |
| 180 | BCW | Persuasion | Intervention functions; Using communication to induce positive or negative feelings or stimulate action |
| 181 | BCW | Training | Intervention functions; Imparting skills |
| 182 | Authors | Potential for delivery of behavior change techniques | *eHealth added value*; *eHealth state-of-the-art*; eHealth (mobile) solutions deliver many additional behavior change techniques (BCTs) that are not possible, for instance, with standard pedometers, such as goal setting, social support, and cues to action. These new techniques embedded within an eHealth framework may move toward helping to tackle one of the key issues of long term sustained behavior |
| 183 | Authors | Technology knowledge gap | *Technology acceptance*; There exists a gap between younger and older generations when it comes to familiarization with technology (e.g., smartphones). The gap can become an issue during feasibility testing, and affect confidence to use a technology even after a familiarization process |
| 184 | DEP | Conceptualization (DEP) | Stage of development where experts decide on the theoretical basis, review the evidence, and plan the development process. Brainstorming sessions can cover how to translate the theory and evidence into practical methods and techniques |
| 185 | DEP | Theoretical basis (Cross-cutting theme) | This theme proposes that the overall development input from the target group and stakeholders must be balanced by ensuring the integration of underlying theory in the intervention |
| 186 | DEP | Implementation focus (Cross-cutting theme) | This theme endorses a focus on implementation from the beginning of the development process. There is little point developing an effective intervention if there is no chance of getting it to the target audience after the study |
| 187 | DEP | Formative research | Stage of development when research is performed with participants from the target population. In particular, to determine how the intended audience uses relevant technology and how a health-related program could be integrated within that context |
| 188 | DEP | Pilot study | Stage of development where the objective is to obtain further feedback from the target audience on the intervention as it would be delivered in its final form. Other objectives can be to test processes for recruitment, registration, data collection, and technical aspects |
| 189 | DEP | Pretesting | Stage of development that utilizes a variety of methods for pretesting early intervention content. At this stage it is important to accept that not all participants would appreciate or relate to every component of the intervention, so key aspects could be repeated in different formats and contexts as possible |
| 190 | DEP | Target population involvement (Cross-cutting theme) | This theme reflects the importance of target audience involvement in order to ensure an eHealth intervention is engaging and useful. Input should be appreciated and should be able to be acted upon by altering the intervention accordingly |
| 191 | MedFit | Contact us | *Practical application*s; Facilitating technical support and information |
| 192 | MedFit | Exercise guidance | *Practical application*s; Facilitation of video and teaching points used to guide participants through different exercises |
| 193 | MedFit | Healthy lifestyle guidance | *Practical application*s; Provision of tips and recommendations on healthy lifestyle components |
| 194 | MedFit | Notifications | *Practical application*s; Providing notifications to help initiate and maintain the behavior change |
| 195 | MedFit | Feedback on progress | *Practical application*s; Provision of feedback on activity level |
| 196 | MedFit | Social interaction | *Practical application*s; Provision of support to participants by encouraging social interaction through the eHealth technology |
| 197 | SCT | Goals | *Parameters of effectiveness*; The health goals people set for themselves and the concrete plans and strategies for realizing them |
| 198 | SCT | Knowledge | *Parameters of effectiveness*; Knowledge of health risks and benefits of different health practices |
| 199 | SCT | Outcome expectations | *Parameters of effectiveness*; Outcome expectations about the expected costs and benefits for different health habits |
| 200 | SCT | Perceived environmental impediments and facilitators | *Parameters of effectiveness*; The perceived facilitators and social and structural impediments to the desired changes |
| 201 | SCT | Perceived self-efficacy | *Parameters of effectiveness*; Perceived self-efficacy that one can exercise control over one’s health habits |
| 202 | UTAUT | Behavioral intention | Determinants of *Technology acceptance*; The intention to use a technology |
| 203 | UTAUT | Effort expectancy | Determinants of *Technology acceptance*; The degree of ease associated with consumers' use of technology |
| 204 | UTAUT | Experience | Determinants of *Technology acceptance*; The passage of time from the initial use of a technology by an individual |
| 205 | UTAUT | Facilitating conditions | Determinants of *Technology acceptance*; Consumers' perceptions of the resources and support available to perform a behavior |
| 206 | UTAUT | Habit | Determinants of *Technology acceptance*; The extent to which people tend to perform behaviors automatically because of learning, also equated with automaticity |
| 207 | UTAUT | Hedonic motivation | Determinants of *Technology acceptance*; The fun or pleasure derived from using a technology |
| 208 | UTAUT | Performance expectancy | Determinants of *Technology acceptance*; The degree to which using a technology will provide benefits to consumers in performing certain activities |
| 209 | UTAUT | Price value | Determinants of *Technology acceptance*; Consumers' cognitive tradeoff between the perceived benefits of the applications and the monetary cost for using them |
| 210 | UTAUT | Social influence | Determinants of *Technology acceptance*; The extent to which consumers perceive that important others (e.g., family and friends) believe they should use a particular technology |
|  | **McGillicuddy et al. 2012** | | |
| 211 | Authors | Clinical inertia | The failure to establish appropriate targets and escalate treatment to achieve treatment goals. |
| 212 | Authors | Positive feedback and medication reminders | *Development assumption*; *Self-management assumption*; Provision of positive feedback over time and motivational personalized messages can stimulate self-efficacy and autonomous regulation, leading to changes in desired behavioral outcomes |
| 213 | IDM | Converging | Initial development of a technology guided by the input of previous phases of data collection and proposed solutions |
| 214 | IDM | Diverging | Conducting qualitative data from target group to identify further refinements needed and to scale-up the technology. |
| 215 | IDM | Experimenting | Conducting initial proof-of-concept trials of the technology |
| 216 | IDM | Observing | Meeting with or directly observing the target group to discuss main issues and context of the problem |
| 217 | IDM | Problem selecting | Identification of most commonly reported issues and strategies for sustainable behavior change. For example, via literature reviews of theories, previous trials, or collecting views from the target group |
| 218 | IDM | Reframing and accommodating | Proposing initial solutions and contextual requirements for success to resolve the issues |
| 219 | IDM | Solution finding | Determine if viewpoints are consistent across larger number of patients, both in terms of the identified issues and the proposed solutions |
| 220 | SDT | Autonomous regulation | *Parameters of effectiveness*; According to Self-Determination Theory both identified regulation (when one personally endorses or identifies with the value or importance of a behavior or health practice) and integrated regulation (when a person doesn’t just value the behavior but has also aligned it with other central values and lifestyle patterns) are autonomous and are associated with enhanced maintenance and transfer of behavior change |
| 221 | SDT | Intrinsic motivation | *Parameters of effectiveness*; Doing an activity for the inherent satisfaction of the activity itself |
| 222 | SDT | Self-efficacy (perceived competence) | *Parameters of effectiveness*; Level of perceived competence |
|  | **Rahimi et al. 2015** | | |
| 223 | Authors | Adaptive and user friendly eHealth | An adaptive and user-friendly monitoring system can enable a wide range of patients to monitor their health status regularly. This can be achieved through automated collection of user interactions with the system (log data) and through remote automatic updates without required input from the patients |
| 224 | Authors | Tailoring to user’s capacity and preferences | The need to tailor eHealth (monitoring) systems to user’s capacity and preferences should be given sufficient priority during the development stage |
| 225 | TAM | Perceived ease of use | Determinants of technology adoption; Perceived ease of use is a significant secondary determinant of people's intentions to use computers |
| 226 | TAM | Perceived usefulness | Determinants of technology adoption; Perceived usefulness is a major determinant of people's intentions to use technology |
| 227 | TAM | Usage intentions | Determinants of technology adoption; People's technology use can be predicted reasonably well from their intentions |
|  | **Srinivas et al. 2017** | | |
| 228 | Authors | Publishing conventions and space constraints | *Development assumption*; *Development state-of-the-art*; The challenge in finding studies that describe multiple methods and a variety of data collection approaches used during eHealth development is suspected to be an artifact of publishing conventions and space constraints, as much as if not more than the nature of actual research being performed |
| 229 | Authors | Multidisciplinary non-participatory design | *Limitations of study/project*; A development team can be multidisciplinary by necessity but not actively involve patients in the design approach. This can occur due to logistic challenges and lack of experience with the target population. |
| 230 | Authors | Overcoming the inertia of disengagement | *Development assumption*; Design goals should not assume ‘baseline levels of engagement’ but rather promote it, if even for a relatively short period of time |
| 231 | Authors | Self-management (sociotechnical concept) | Self-management is a complex set of processes with multiple goals, embedded in a complex, interacting sociotechnical system, and associated with multiple outcomes. Chronic disease management work is collaborative in that it is performed by a collective group, with agents including patients, informal caregivers, and healthcare professionals. In many cases, however, there are multiple breakdowns and barriers, which can be addressed by technologies or other interventions. |
| 232 | Authors | Waterfall approach | *Limitations of study/project*; Long and sequential stages, of development, as opposed to an agile approach of rapid cycles of product development and feedback |
| 233 | PWL | Biomedical lens | Integrates aspects of patients and their environment such as age, gender, current and past diagnoses, genetic, behavioral risk factors, biomarkers, current treatment plan, past and future clinical appointments. |
| 234 | PWL | Clinical technologies | Biomedical lens |
| 235 | PWL | Collaborative technologies | Patient work lens |
| 236 | PWL | Consumer technologies | Personal skills and behavioral lens |
| 237 | PWL | Patient | Patient work lens; Includes physiology, social-behavioral-characteristics) |
| 238 | PWL | Patient work lens | The key premise that health work such as chronic disease management is not merely biological or psychological, but rather biopsychosocial. This integrates the broader aspects of the patient’s work system (person, tasks, tool, etc.) and the patient’s work activity (everyday life work, invisible work, etc.) |
| 239 | PWL | Patient’s work activity | Patient work lens |
| 240 | PWL | Patient’s work system | Patient work lens |
| 241 | PWL | Personal skills and behavior lens | Integrates aspects of patients and their environment such as knowledge, cognitive functioning, health literacy, attitude, motivation, readiness to change, behaviors, social support, self- efficacy, and self-regulatory skills |
| 242 | SEIPS 2.0 | Collaborative professional-patient work | Work processes; Work involving the active participation of both professionals and nonprofessionals |
| 243 | SEIPS 2.0 | Desirable / Undesirable | Outcomes; The outcomes might reflect the goals of different stakeholders such as clinicians, organizational leaders, regulators, payers and, perhaps most importantly, patients |
| 244 | SEIPS 2.0 | External environment | Work system |
| 245 | SEIPS 2.0 | Feedback loop | Planned and unplanned adaptation of work structures and processes over time |
| 246 | SEIPS 2.0 | Health-related work | Work processes; The exertion of effort and investment of time on the part of patients or family members to produce or accomplish something |
| 247 | SEIPS 2.0 | Internal environment | Work system |
| 248 | SEIPS 2.0 | Invisible work | Work processes; When a person’s collaborative work is unacknowledged |
| 249 | SEIPS 2.0 | Organization | Work system |
| 250 | SEIPS 2.0 | Organizational | Outcomes |
| 251 | SEIPS 2.0 | Outcomes | They are states or conditions resulting from the work process and can be proximal or distal, desirable or undesirable, they can refer to patient, professional, or organizational outcomes |
| 252 | SEIPS 2.0 | Patient (outcomes) | Outcomes |
| 253 | SEIPS 2.0 | Patient work | Work processes; Work performed by non-professionals without HCP involvement |
| 254 | SEIPS 2.0 | Person(s) | Work system |
| 255 | SEIPS 2.0 | Professional | Outcomes |
| 256 | SEIPS 2.0 | Professional work | Work processes; Works performed by HCPs without active patient or family involvement |
| 257 | SEIPS 2.0 | Proximal/distal | Outcomes; Proximal and distal outcomes can be distinguished given that some outcomes may be the immediate result of work processes while others are further down the causal chain and may only emerge over time |
| 258 | SEIPS 2.0 | Tasks | Work system |
| 259 | SEIPS 2.0 | Tools & Technology | Work system |
| 260 | SEIPS 2.0 | Work processes | Can be physical, cognitive, social/behavioral (performance) |
| 261 | SEIPS 2.0 | Work system | The sociotechnical systems model or work system model depicts the interactions between people and other social, technical, and environmental elements. |
| 262 | UCD-CHIT | Design (UCD-CHIT) | Design abstract representations or more traditional artifacts such as wireframes or user interface prototypes. |
| 263 | UCD-CHIT | Evaluate | Evaluate the designs against initial understandings of users and goals. |
| 264 | UCD-CHIT | Study (analysis) phase | Seek to understand the users, their tasks, goals, different aspects of the surrounding environment, and broader contexts. |
|  | **Triantafyllidis et al. 2015** | | |
| 265 | Authors | Iterative personalization | *Development assumption*; *Evidence gap*; Iterative personalization of eHealth services (e.g., remote health monitoring) according to the patient’s ongoing healthcare and usability needs is a vital element for successful implementation. Regular adaptation to changing circumstances is therefore necessary for widespread and sustained adoption. |
| 266 | IRA | Remote delivery of system refinements | Following delivery of the eHealth technology at the patient’s home, remote reﬁnements of the system can be initiated, thereby saving valuable human resources |
| 267 | IRA | Evaluation | The system’s evaluation corresponds to the systematic exploration of usability. User engagement is also considered an additional important factor for usability. This can be quantiﬁed by recording how often speciﬁc system features are used, indicating the extent to which the system is being adopted |
| 268 | IRA | Understanding patients’ monitoring needs | This cycle includes iterative qualitative and quantitative assessments of the patient’s monitoring requirements by the development team. The iterative approach is necessary because the patient’s monitoring requirements may change over time (e.g., monitoring devices used, frequency of system use, requested service features) during system run-time operation |
| 269 | IRA | Development | During this cycle, the development of the eHealth technology takes place. Functional features are developed and added by the engineers, if applicable, existing features are improved or removed to attain patient satisfaction. Design, coding, encapsulation into software applications and unit testing, are all included in this cycle |
|  | **Villalba et al. 2009** | | |
| 270 | Authors | Adaptation to personal routines | *Mode of delivery and implementation*; *Development assumption*; *Heterogeneity (Heart Failure)*; The technology must fit intuitively, naturally and in a user-friendly way the user’s health and mental status, preferences, and recommended medical protocol. |
| 271 | Authors | User interaction | *Development aim*; *Development assumption*; User interaction with the technology, both through explicit and implicit input. |
| 272 | HPI | Implicit (technology-human) interaction | The interaction of a human with the environment and with artifacts (e.g., sensors) which is aimed to accomplish a goal. Within this process the system acquires implicit input from the user and may present implicit output to the user |
| 273 | HPI | Implicit input | Actions and behavior of humans, which are done to achieve a goal and are not primarily regarded as interaction with a technology, but captured, recognized and interpreted by a system as input |
| 274 | HPI | Implicit output | The output of a technology which is not directly related to an explicit input by the user and which is seamlessly integrated with the environment and the task of the user |
| 275 | HPI | Medical context | It can integrate the treatment, protocols, vital signs, or symptoms which are monitored by the clinical or social services around the patient |
| 276 | HPI | Patient context | It can integrate the human factors, patient’s routine, health status, or the immediate system around the patient (e.g., sensors) |
| 277 | HPI | Social and business context | It integrates the familiar or social organization of the patient, as well as the business model that connects him or her with formal, informal caregivers, volunteers, or relatives |
| 278 | ISD | Conceptualization (ISD) | Through initial input and research, a first concept model of the eHealth solution is proposed as several contexts and several interaction loops, for instance, based on the Holistic Patient Interaction Model. The concept is validated with stakeholders via identification of requirements and through prototype design and implementation |
| 279 | ISD | Deployment | Technical bugs identified in the previous phase are fixed and the design is refine. The complete system is deployed and analyzed again with real users. Conclusions and future work are derived. |
| 280 | ISD | Implementation | The model is reviewed according to the previous phase and the eHealth solution is assessed in a real environment with real users. The system is validated with stakeholders to update the system requirements and through system design and implementation |
|  | **Walsh et al. 2018a** | | |
| 281 | 5E | Easy to learn | Dimensions of usability; The easy to learn dimension evaluates how the system supports both initial and long-term use |
| 282 | 5E | Effective | Dimensions of usability; The effective dimension explores how completely and accurately a user can complete a task |
| 283 | 5E | Efficient | Dimensions of usability; The efficient dimension refers to how quickly the task can be done |
| 284 | 5E | Engaging | Dimensions of usability; The engaging dimension highlights how well the interface guides the user intuitively through the task |
| 285 | 5E | Error | Dimensions of usability; The error tolerant dimension assesses how well a system can avoid user-generated errors and also how the system aids the user in overcoming this error |
| 286 | BCW/COM-B | Automatic Motivation | *Parameters of effectiveness*; Sources of behavior; Motivation describes the brain processes that energize and direct behavior and includes both automatic motivation (e.g., habits) and reflective motivation (e.g., cost-benefit decision making) |
| 287 | BCW/COM-B | Physical Capability | *Parameters of effectiveness*; Sources of behavior; Capability is the individual’s ability to perform a behavior and includes both physical capability (e.g., skills) and psychological capability (e.g., knowledge) |
| 288 | BCW/COM-B | Physical Opportunity | *Parameters of effectiveness*; Sources of behavior; Opportunity describes the factors that lie outside the individual that facilitate or prompt behavior and includes both physical opportunity (e.g., affordability) and social opportunity (e.g., cultural norms) |
| 289 | BCW/COM-B | Reflective Motivation | *Parameters of effectiveness*; Sources of behavior; Motivation describes the brain processes that energize and direct behavior and includes both automatic motivation (e.g., habits) and reflective motivation (e.g., cost-benefit decision making) |
| 290 | BCW/COM-B | Social Opportunity | *Parameters of effectiveness*; Sources of behavior; Opportunity describes the factors that lie outside the individual that facilitate or prompt behavior and includes both physical opportunity (e.g., affordability) and social opportunity (e.g., cultural norms) |
| 291 | BCW/COM-B | Policy categories | They represent types of decisions made by authorities that help to support and enact the interventions |
| 292 | BCW/COM-B | Intervention functions | Intervention functions are identified as the broad mechanisms through which an intervention can effect change |
| 293 | BCW/COM-B | Guidelines | Policy categories; Creating documents that recommend or mandate practice |
| 294 | BCW/COM-B | Service provision | Policy categories; Delivering a service |
| 295 | BCW/COM-B | Social planning | Policy categories; Designing and or controlling the physical or social environment |
| 296 | BCW/COM-B | Education (Intervention function) | *Parameters of effectiveness*; Intervention functions; Increasing knowledge or understanding |
| 297 | BCW/COM-B | Enablement | *Parameters of effectiveness*; Intervention functions; Increasing means/reducing barriers to increase capability |
| 298 | BCW/COM-B | Environmental restructuring | *Parameters of effectiveness*; Intervention functions; Changing the physical or social context |
| 299 | BCW/COM-B | Modelling (BCW) | *Parameters of effectiveness*; Intervention functions; Providing an example for people to aspire to or imitate |
| 300 | BCW/COM-B | Persuasion | *Parameters of effectiveness*; Intervention functions; Using communication to induce positive or negative feelings or stimulate action |
| 301 | BCW/COM-B | Psychological Capability | *Parameters of effectiveness*; Sources of behavior; Capability is the individual’s ability to perform a behavior and includes both physical capability (e.g., skills) and psychological capability (e.g., knowledge) |
| 302 | BCW/COM-B | Training | *Parameters of effectiveness*; Intervention functions; Imparting skills |
| 303 | Authors | Adequate reporting of intervention design and content | *eHealth state-of-the-art*; *Evidence gap*; Adequate description of intervention content, guiding theoretical frameworks, and user testing protocols need to be made available to aid future intervention development |
| 304 | Authors | Tailoring and personalization | *Heterogeneity (CVD)*; An individual assessment (e.g., psychological readiness for change) and tailored and personalized features can be useful to achieve health behavior change, to empower patients to make choices and direct them to the most appropriate content for them at a specific time |
| 305 | Authors | Incremental stepped development and evaluation | *Framework/theory/model state-of-the-art*; Incremental stepped approaches to developing and evaluating behavior change interventions using technology are vital, as per the Medical Research Council (MRC) and behavior change wheel (BCW) frameworks |
| 306 | Authors | Interdisciplinary methods | *Development state-of-the-art*; *Limitations of study/project*; There is a growing need to consider adopting methods from other disciplines rather than using deployment–evaluation cycles [54]. Theories, models, and methods to support this approach can be found in engineering and related sectors (e.g., use of factorial or fractionated evaluation designs that have been utilized well within the HCI sphere) |
| 307 | PATHway | Assessment | *Practical application*s; Inclusion of different types of assessment, such as lifestyle, behavioral change, and health and fitness |
| 308 | PATHway | Behavioral change | *Practical application*s; Inclusion of features such as health behavior change goal selection, calendar logging of health behaviors, or calendar for events/exercise sessions provided by the eHealth system |
| 309 | PATHway | Education (Practical application) | *Practical application*s; For example, through inclusion of ‘MyHealthyLifestyle’ content (e.g., ‘ask the expert’ videos) and health behavior change notifications (SMS/Emails) |
| 310 | PATHway | Familiarization phase | *Practical application*s; For example, through a preliminary phase of face-to-face exercise consultation |
| 311 | PATHway | Feedback | *Practical application*s; Inclusion of different types of feedback, such as: - Sensor instructions for wearing prior to use - On-screen visual/verbal avatar instructions prior to exercise - On-screen positive reinforcement during Exerclass (related to exercise accuracy) - Sensor physiological feedback (i.e. Microsoft Band 2, blood pressure monitor, Zensor) - End of exercise summary feedback - Good habits visualization (following lifestyle assessment) |
| 312 | PATHway | Home exercising | *Practical application*s; For example, through facilitating a way to practice individual exercise, promoting different physical activity options (e.g., ExerClass/ExerGame/Active lifestyle), and including several features such as multiplayer classes, decision-support for exercise adaptation, or exercise prescription |
| 313 | PATHway | Home platform access | *Practical application*s; For example, the inclusion of a dashboard (front-end visualization of use), technical support, or training videos/manual |
| 314 | PATHway | Mediators of behavior change | Outcomes of cardiac rehabilitation; Knowledge, goals, outcome expectations, perceived environmental impediments and facilitators) |
| 315 | PATHway | Self-efficacy (outcome of cardiac rehabilitation) | Outcomes of cardiac rehabilitation |
| 316 | PBA | Design (PBA) | Creation of guiding principles to help developers summarize and easily refer to features of the intervention identified as central to achieving the intervention objectives |
| 317 | PBA | Development and evaluation of acceptability and feasibility | All intervention components evaluated in detail and optimized from user perspective |
| 318 | PBA | Planning | Identification of key behavioral issues, needs, and challenges the intervention must address |
| 319 | SCT | Goals | *Parameters of effectiveness*; The health goals people set for themselves and the concrete plans and strategies for realizing them |
| 320 | SCT | Knowledge | *Parameters of effectiveness*; Knowledge of health risks and benefits of different health practices |
| 321 | SCT | Outcome expectations | *Parameters of effectiveness*; Outcome expectations about the expected costs and benefits for different health habits |
| 322 | SCT | Perceived environmental impediments and facilitators | *Parameters of effectiveness*; The perceived facilitators and social and structural impediments to the changes they seek |
| 323 | SCT | Self-efficacy | *Parameters of effectiveness*; Perceived self-efficacy that one can exercise control over one’s health habits |
|  | **Walsh et al. 2018b** | | |
| 324 | COM-B | Motivation (COM-B) | *Parameters of effectiveness*; Sources of behavior; Motivation describes the brain processes that energize and direct behavior and includes both automatic motivation (e.g., habits) and reflective motivation (e.g., cost-benefit decision making) |
| 325 | COM-B | Physical Capability | *Parameters of effectiveness*; Sources of behavior; Capability is the individual’s ability to perform a behavior and includes both physical capability (e.g., skills) and psychological capability (e.g., knowledge) |
| 326 | COM-B | Physical Opportunity | *Parameters of effectiveness*; Sources of behavior; Opportunity describes the factors that lie outside the individual that facilitate or prompt behavior and includes both physical opportunity (e.g., affordability) and social opportunity (e.g., cultural norms) |
| 327 | COM-B | Psychological Capability | *Parameters of effectiveness*; Sources of behavior; Capability is the individual’s ability to perform a behavior and includes both physical capability (e.g., skills) and psychological capability (e.g., knowledge) |
| 328 | COM-B | Social Opportunity | *Parameters of effectiveness*; Sources of behavior; Opportunity describes the factors that lie outside the individual that facilitate or prompt behavior and includes both physical opportunity (e.g., affordability) and social opportunity (e.g., cultural norms) |
| 329 | SEM | Community level | From the perspective of the Social Ecological Model, a wide range of individuals are involved at various points of the CVD illness journey, through various levels represented by a ‘rainbow band’. The fourth band of the SEM rainbow surrounds the organizational band and represents activities implemented at the community level. These activities are intended to facilitate individual behavior change through by leveraging resources and participation of community-level institutions such as comprehensive patient coalitions, tribal health departments, media, and community advocacy groups, which represent potential sources of community communication and support. |
| 330 | SEM | Individual level | From the perspective of the Social Ecological Model, a wide range of individuals are involved at various points of the CVD illness journey, through various levels represented by a ‘rainbow band’. The innermost band of the SEM represents the individual who might be affected by the eHealth technology and its aims |
| 331 | SEM | Interpersonal level | From the perspective of the Social Ecological Model, a wide range of individuals are involved at various points of the CVD illness journey, through various levels represented by a ‘rainbow band’. The second band of the SEM rainbow surrounds the individual band and represents prevention activities implemented at the interpersonal level. These activities are intended to facilitate individual behavior change by affecting social and cultural norms and overcoming individual-level barriers. Friends, family, health care providers, community health workers or promotors, and patient navigators represent potential sources of interpersonal messages and support |
| 332 | SEM | Organizational level | From the perspective of the Social Ecological Model, a wide range of individuals are involved at various points of the CVD illness journey, through various levels represented by a ‘rainbow band’. The third band of the SEM rainbow surrounds the interpersonal band and represents prevention activities implemented at the organization level. These activities are intended to facilitate individual behavior change through by influencing organizational systems and policies. Health care systems, employers or worksites, health care plans, local health departments, tribal urban health clinics, and professional organizations represent potential sources of organizational messages and support. |
| 333 | SEM | Policy level | From the perspective of the Social Ecological Model, a wide range of individuals are involved at various points of the CVD illness journey, through various levels represented by a ‘rainbow band’. The fifth and outermost band of the SEM rainbow surrounds the community band and represents prevention activities at the policy level. These activities involve interpreting and implementing existing policy. Federal, state, local, and tribal government agencies may support policies that promote healthy behavior, including screening. |

# Translation of key metaphors

## Clusters of key metaphors

| **Cluster 1: eHealth is a participatory development process** | |
| --- | --- |
| *Band et al 2017*   1. Integrating theory-, evidence- and person based approaches   *Chantler et al 2016*   1. Diversity of user experiences   *Development and Evaluation Process for mHealth*   1. Formative research 2. Pilot study 3. Pretesting 4. Target population involvement (Cross-cutting theme)   *Intervention Mapping*   1. Needs assessment   *Iterative Design Model*   1. Observing 2. Diverging   *Iterative Refinement and Patient Participatory Approach*   1. Understanding patients' monitoring needs 2. Evaluation   *Iterative Software Design Process*   1. Conceptualization (ISD) 2. Implementation 3. Deployment   *Medical Research Council’s (MRC) Guidance for Developing and Evaluating Complex Interventions*   1. User involvement | *Person-Based Approach*   1. Planning 2. Design (PBA) 3. Development and evaluation of acceptability and feasibility   *Social Ecological Model*   1. Individual level 2. Interpersonal level 3. Community level 4. Organizational level 5. Policy level   *Usability Framework*   1. Ethnography   *User-centred design (Ad hoc; Baek et al 2018)*   1. Surveys and interviews with patients 2. Focus group interview with HCP   *User-centred design (Monk 2000)*   1. Understanding the work 2. Understanding the work context 3. Testing a top level design against your understanding of the work 4. User testing of more detailed prototypes   *User-centred design of Consumer-facing Health*   1. Study (analysis) phase 2. Design (UCD-CHIT) 3. Evaluate |
|  |  |

| **Cluster 2: eHealth development creates new infrastructures for improving health care, health, and well-being** | |
| --- | --- |
| *Band et al 2016*   1. Tailored, personalized, and timely support   *Behavior Change Wheel/Capability, Opportunity, Motivation-Behavior model*   1. Policy categories 2. Guidelines 3. Service provision 4. Social planning   *Development and Evaluation Process for mHealth*   1. Conceptualization (DEP)   *Intervention* *Mapping*   1. Program plan | *Iterative Refinement and Patient Participatory Approach*   1. Remote delivery of system refinements   *Social Ecological Model*   1. Individual level 2. Interpersonal level 3. Community level 4. Organizational level 5. Policy level   *Unified Theory of Acceptance and Use of Technology model*   1. Facilitating conditions   *Walsh et al 2018a*   1. Interdisciplinary methods |

| **Cluster 3: eHealth development is intertwined with implementation** | |
| --- | --- |
| *Business Model Canvas*   1. Key partners 2. Key activities 3. Key resources 4. Value propositions 5. Customer relationships 6. Channels 7. Customer segments 8. Cost structure 9. Revenue streams   *Development and Evaluation Process for mHealth*   1. Formative research 2. Implementation focus (Cross-cutting theme)   *Intervention Mapping*   1. Adaptation and implementation plan | *Iterative Design Model*   1. Diverging   *Iterative Refinement and Patient Participatory Approach*   1. Remote delivery of system refinements   *Iterative Software Design Process*   1. Conceptualization (ISD) 2. Implementation   *Person-Based Approach*   1. Development and evaluation of acceptability and feasibility   *Realistic Evaluation Framework*   1. Design a field study to evaluate CMO hypotheses |
|  |  |

| **Cluster 4: eHealth development is coupled with persuasive design** | |
| --- | --- |
| *Band et al 2017*   1. Integrating theory-, evidence- and person based approaches   *Behavior Change Wheel/Capability, Opportunity, Motivation-Behavior model*   1. Automatic Motivation   *Unified Theory of Acceptance and Use of Technology model*   1. Facilitating conditions 2. Habit | 1. Hedonic motivation 2. Performance expectancy 3. Social influence   *Walsh et al 2018a*   1. Tailoring and personalization |

| **Cluster 5: eHealth development requires continuous evaluation cycles** | |
| --- | --- |
| *Development and Evaluation Process for mHealth*   1. Conceptualization (DEP) 2. Pretesting 3. Pilot study   *Intervention Mapping*   1. Evaluation plan   *Iterative Design Model*   1. Solution finding 2. Converging 3. Experimenting   *Iterative Refinement and Patient Participatory Approach*   1. Understanding patients' monitoring needs 2. Evaluation   *Iterative Software Design Process*   1. Conceptualization (ISD) 2. Implementation 3. Deployment   *Medical Research Council’s (MRC) Guidance for Developing and Evaluating Complex Interventions*   1. Preclinical or theoretical 2. Modelling (MRC) 3. Exploratory or pilot trial 4. Development phase 5. Parallel approach 6. Stepwise approach | *Person-Based Approach*   1. Development and evaluation of acceptability and feasibility   *Realistic Evaluation Framework*   1. Analyze data in terms of CMO hypotheses 2. Synthesis of new data into refine CMO hypotheses   *Srinivas et al 2017*   1. Agile vs Waterfall approach   *Usability Framework*   1. Ethnography   *User-centred design (Ad hoc; Baek et al 2018)*   1. Mock-up design 2. Usability test   *User-centred design of Consumer-facing Health*   1. Study (analysis) phase 2. Design (UCD-CHIT) 3. Evaluate   *Walsh et al 2018a*   1. Incremental stepped development and evaluation 2. Interdisciplinary methods |
|  | |
| **Cluster 6: Behavior change** | |
| *Athilingam et al 2018b*   1. Patient activation   *Band et al 2016*   1. Tailored, personalized, and timely support   *Bartlett et al 2014*   1. Meaningful feedback   *Behavior Change Wheel/Capability, Opportunity, Motivation-Behavior model*   1. Motivation (COM-B) 2. Automatic Motivation 3. Psychological Capability 4. Social Opportunity   *Chantler et al 2016*   1. Real-time feedback in self-care   *CHF PSMS (Practical applications^a^ of overarching project)*   1. Feedback on activity 2. Feedback on activity performance 3. Goal setting related to user’s lifestyle   *Control Theory Framework for Personality-Social, Clinical, and Health Psychology*   1. Self-monitoring   *Duff et al 2018*   1. Potential for delivery of behavior change techniques   *HeartMapp (Practical applications^a^ of overarching project)*   1. Assessment 2. CHF info/knowledge   *HOME BP (Practical applications^a^ of overarching project)*   1. Health care provider: Support for lifestyle changes & BP monitoring 2. Patient: Self-monitoring & BP entry 3. Patient: Self-selected lifestyle changes   *Information, Motivation, Behavioral skills model*   1. Information 2. Motivation (IMB) 3. Behavioral skills | *McGillicuddy et al 2012*   1. Positive feedback and medication reminders   *Normalization Process Theory*   1. Interactional workability 2. Skillset workability 3. Communal specification 4. Differentiation 5. Individual specification 6. Enrolment 7. Initiation 8. Legitimation 9. Individual appraisal 10. Reconfiguration 11. Systematization   *PATHway (Practical applications^a^ of overarching project)*   1. Behavioral change 2. Familiarization phase 3. Feedback 4. Home exercising 5. Home platform access   *Patient Work Lens for Consumer-facing Health*   1. Personal skills and behavior lens   *Social Cognitive Theory*   1. Goals 2. Outcome expectancy 3. Outcome expectations 4. Perceived environmental impediments and facilitators 5. Perceived self-efficacy 6. Self-efficacy   *Srinivas et al 2017*   1. Self-management (sociotechnical concept) |
| ^a^Practical applications were defined as the translations of theoretical methods of behavior change to practical intervention elements. | |

| **Cluster 7: Technology adoption** | |
| --- | --- |
| *5E (Usability) Approach*   1. Easy to learn 2. Effective 3. Efficient 4. Engaging 5. Error   *Baek et al 2018*   1. Bidirectional service model   *Band et al 2016*   1. Tailored, personalized, and timely support   *Business Model Canvas*   1. Key partners 2. Key activities 3. Key resources 4. Value propositions 5. Customer relationships 6. Channels 7. Customer segments 8. Cost structure 9. Revenue streams   *Chantler et al 2016*   1. Connection 2. Diversity of user experiences 3. Personalization and tailoring 4. Routine   *CHF PSMS (Practical applications^a^ of overarching project)*   1. Acceptability and fit of hardware in everyday life 2. User-centred design process undertaken   *Congratulate, Ask, Reassure, Encourage approach*   1. Ask   *Domestication of Technology Theory*   1. Domestication of technology | 1. Appropriation 2. Objectification 3. Incorporation 4. Conversion   *Duff et al 2018*   1. Technology knowledge gap   *Normalization Process Theory*   1. Relational integration   *Rahimi et al 2015*   1. Adaptive and user friendly eHealth 2. Tailoring to user’s capacity and preferences   *Srinivas et al 2017*   1. Overcoming the inertia of disengagement   *Technology acceptance models*   1. Perceived ease of use 2. Perceived usefulness 3. Usage intentions   *Triantafyllidis et al 2015*   1. Iterative personalization   *Unified Theory of Acceptance and Use of Technology model*   1. Behavioral intention 2. Effort expectancy 3. Experience 4. Facilitating conditions 5. Habit 6. Hedonic motivation 7. Performance expectancy 8. Price value 9. Social influence   *Villalba et al 2009*   1. Adaptation to personal routines 2. User interaction |
| ^a^Practical applications were defined as the translations of theoretical methods of behavior change to practical intervention elements. | |

| **Cluster 8: Health-related outcomes** | |
| --- | --- |
| *Bartlett et al 2014*   1. Measuring changes in knowledge   *Behavior Change Wheel/Capability, Opportunity, Motivation-Behavior model*   1. Education (Intervention function) 2. Enablement 3. Environmental restructuring 4. Policy categories   *CHF PSMS (Practical applications^a^ of overarching project)*   1. Ability to walk further, lose weight or meet personal goals 2. All patients are able to use the system and continue to use it for the duration of the evaluation 3. Balance between activity and rest 4. Behavior change that is sustainable over the long term 5. Continued engagement with system 6. Improved symptom control, ideally reducing need for health professional involvement. 7. Increased levels of knowledge about self-management. 8. Self-management tailored to patient’s condition   *HeartMapp (Practical applications^a^ of overarching project)*   1. Adherence to medication 2. Diet 3. Global health 4. HF knowledge 5. HF self-management 6. Improved self-care skills 7. Physical activity 8. Quality of life 9. Reduce readmissions | *HOME BP (Practical applications^a^ of overarching project)*   1. Patient lifestyle change 2. Patient medication adherence and titration   *McGillicuddy et al 2012*   1. Clinical inertia   *PATHway (Practical applications^a^ of overarching project)*   1. Mediators of behavior change 2. Self-efficacy (outcome of cardiac rehabilitation)   *Systems Engineering Initiative for Patient Safety 2.0*   1. Collaborative professional-patient work 2. Desirable / Undesirable 3. Health-related work 4. Outcomes 5. Patient (outcomes) 6. Patient work 7. Proximal/distal 8. Work processes |
| ^a^Practical applications were defined as the translations of theoretical methods of behavior change to practical intervention elements. | |

| **Key metaphors without a cluster (Part 1)** | |
| --- | --- |
| *Band et al 2017*   1. Theoretical modelling   *Behavior Change Wheel/Capability, Opportunity, Motivation-Behavior model*   1. Modelling (BCW) 2. Persuasion 3. Training 4. Intervention functions 5. Physical Capability 6. Physical Opportunity 7. Reflective Motivation   *CHF PSMS (Practical applications^a^ of overarching project)*   1. Access to technical support 2. Co-morbidities 3. Condition stability 4. Consideration of physical and psychological barriers to lifestyle changes 5. eHealth literacy 6. Engagement and happiness to use the system 7. Engagement with variety of lifestyle changes 8. Information and advice section 9. Knowledge about self-management 10. Lack of critical problems with technology 11. Over activity / rest cycle 12. Patient activation (CMO hypothesis) 13. Self-monitoring and tailored feedback provision 14. Self-monitoring of symptoms 15. Technology usage in a home setting 16. Walking intervention   *Common-Sense Model of Self-Regulation*   1. Illness beliefs 2. Treatment beliefs   *Congratulate, Ask, Reassure, Encourage approach*   1. Congratulate 2. Encourage 3. Reassure | *Development and Evaluation Process for mHealth*   1. Theoretical basis (Cross-cutting theme)   *HeartMapp (Practical applications^a^ of overarching project)*   1. Education (Practical application) 2. Exercises and feedback 3. Medication list and reminder alerts 4. Personal Health Buddy/Coach 5. Real-time vital signs monitoring 6. Stats   *Holistic Patient Interaction model*   1. Implicit (technology-human) interaction 2. Implicit input 3. Implicit output 4. Medical context 5. Patient context 6. Social and business context   *HOME BP (Practical applications^a^ of overarching project)*   1. Automated BP feedback 2. Automated e-mails prompts/reminders 3. Behavioral support provision 4. Enacting medication titration procedures 5. Health care provider: Pre-planned medication titration schedule 6. Health professional engagement with the intervention 7. Patient engagement with the intervention 8. Patient home blood pressure self-monitoring 9. Website sessions (Patient & Health care provider)   *Intervention Mapping*   1. Proximal program objective(s) 2. Selection of theory-based methods   *Iterative Design Model*   1. Problem selecting 2. Reframing and accommodating   *Iterative Refinement and Patient Participatory Approach*   1. Development |
| ^a^Practical applications were defined as the translations of theoretical methods of behavior change to practical intervention elements. | |
| **Key metaphors without a cluster (Part 2)** | |
| *MedFit (Practical applications^a^ of overarching project)*   1. Contact us 2. Exercise guidance 3. Feedback on progress 4. Healthy lifestyle guidance 5. Notifications 6. Social interaction   *Medical Research Council’s (MRC) Guidance for Developing and Evaluating Complex Interventions*   1. Modelling process and outcomes   *Multidimensional Framework For Patient And Family Engagement In Health And Health*   1. Patient engagement   *Normalization Process Theory*   1. CA: Contextual integration 2. CH: Internalization 3. Cognitive participation (CP) 4. Coherence (CH) 5. Collective action (CA) 6. CP: Activation 7. Reflexive monitoring (RM) 8. RM: Communal appraisal   *Patient Work Lens for Consumer-facing Health*   1. Biomedical lens 2. Clinical technologies 3. Collaborative technologies 4. Consumer technologies 5. Patient 6. Patient work lens 7. Patient’s work activity 8. Patient’s work system | *Realistic Evaluation Framework*   1. CMO hypotheses 2. Collect mixed methods data 3. Formulate the initial CMO hypotheses   *Self-Determination Theory*   1. Autonomous motivation 2. Autonomous regulation 3. Intrinsic motivation 4. Need for autonomy 5. Need for competence 6. Need for relatedness 7. Self-efficacy (perceived competence)   *Social Cognitive Theory*   1. Knowledge   *Srinivas et al 2017*   1. Multidisciplinary non-participatory design 2. Publishing conventions and space constraints   *Systems Engineering Initiative for Patient Safety 2.0*   1. External environment 2. Feedback loop 3. Internal environment 4. Invisible work 5. Organization 6. Organizational 7. Person(s) 8. Professional 9. Professional work 10. Tasks 11. Tools & Technology 12. Work system   *Walsh et al 2018a*   1. Adequate reporting of intervention design and content |
| ^a^Practical applications were defined as the translations of theoretical methods of behavior change to practical intervention elements. | |

## Idiomatic reciprocal and refutational translations

| Key metaphor used for synthesis *[source]* | Translated with *[source]* |
| --- | --- |
| **Reciprocal** |  |
| - *Adaptation to personal routines:* Mode of delivery and implementation; Development assumption; Heterogeneity (Heart Failure); The technology must fit intuitively, naturally and in a user-friendly way the user’s health and mental status, preferences, and recommended medical protocol *[Villalba et al. 2009]* | - *Routine [Chantler et al. 2016]* - *Acceptability and fit of hardware in everyday life [Mechanisms; CMO hypotheses; CHF PSMS]* - *Adaptive and user friendly eHealth [Rahimi et al. 2015]* |
| - *Behavioral intention:* Determinants of technology acceptance; The intention to use a technology *[Unified Theory of Acceptance and Use of Technology model]* | - *Usage intentions [Technology acceptance models]* |
| - *Behavioral skills:* Parameters of effectiveness; It includes ensuring that the patient has the specific behavioral tools or strategies necessary to perform behaviors such as daily weighing, symptom assessment, and exercise *[Athilingam et al. 2016; Information, Motivation, Behavioral skills model]* | - *Behavioral skills [Athilingam et al. 2018a; Information, Motivation, Behavioral skills model]* - *Psychological Capability [Band et al. 2017; Walsh et al. 2018a, 2018b; Behavior Change Wheel/ COM-B model]* |
| - *Converging:* In this stage the intervention and technology are developed, integrating the insights from all the previous phases *[Iterative Design Model]* | - *Conceptualization (ISD) [Iterative Software Design process]* - *Design (UCD-CHIT) [User-centred design of Consumer-facing Health IT]* |
| - *Development and evaluation of acceptability and feasibility:* All intervention components evaluated in detail and optimized from user perspective, as proposed by the Person Based Approach. This stage can be operationalized, for example, by collating and analyzing evidence derived from primary mixed- methods research such as a feasibility study *[Person-Based Approach]* | - *Pretesting [Development and Evaluation Process for mHealth]* - *Evaluate [Person-Based Approach]* |
| - *Feedback:* Practical applications; Inclusion of different types of feedback, such as: Sensor instructions for wearing prior to use; On-screen visual/verbal avatar instructions prior to exercise; On-screen positive reinforcement during Exerclass (related to exercise accuracy); Sensor physiological feedback (i.e. Microsoft Band 2, blood pressure monitor, Zensor); End of exercise summary feedback; Good habits visualization (following lifestyle assessment) *[PATHway]* | - *Feedback on activity [Practical applications; CHF PSMS]* - *Feedback on activity performance [Practical applications; CHF PSMS]* |
| - *Implementation:* The model is reviewed according to the previous phase and the eHealth solution is assessed in a real environment with real users. The system is validated with stakeholders to update the system requirements and through system design and implementation *[Iterative Software Design process]* | - *Pilot study [Development and Evaluation Process for mHealth]* - *Experimenting [Iterative Design Model]* - *Exploratory or pilot trial [MRC Guidance for Developing and Evaluating Complex Interventions]* |
| - *Incremental stepped development and evaluation:* Theory/model state-of-the-art; Incremental stepped approaches to developing and evaluating behavior change interventions using technology are vital, as per the Medical Research Council (MRC) and behavior change wheel (BCW) frameworks *[Walsh et al. 2018a]* | - *Stepwise approach [MRC Guidance for Developing and Evaluating Complex Interventions]* |
| - *Self-efficacy*: Parameters of effectiveness; Proposed mediating variables (Logic model); The expectation that one can successfully execute the behavior required to produce the outcomes *[Band et al. 2017; Social Cognitive Theory].* | - *Self-efficacy [Bradbury et al. 2017; Social Cognitive Theory]* - *Self-efficacy [Walsh et al. 2018a; Social Cognitive Theory]* - *Perceived self-efficacy [Social Cognitive Theory]* |
| - *Self-monitoring:* Practical applications; Self-monitoring is a theory-based technique. An intervention designer could hypothesize that including self- monitoring (theory-based mechanism) in their intervention will increase physical activity (outcome) *[Control Theory Framework for Personality-Social, Clinical, and Health Psychology]* | - *Patient: Self-monitoring & BP entry [Practical applications; HOME BP]* |
| - *Tailored, personalized, and timely support:* eHealth added value; eHealth state-of-the-art; Increasingly widespread access to the internet and mobile phones means that eHealth can be accessible to the majority of patients and can be used to provide information and support at any time the patient needs it. eHealth can empower patients by providing better access to personalized information and support for active involvement in treatment and self-management *[Band et al. 2016]* | - *Meaningful feedback [Bartlett et al. 2014]* - *Positive feedback and medication reminders [McGillicuddy et al. 2012]* - *Real-time feedback in self-care [Chantler et al. 2016]* - *Iterative personalization [Triantafyllidis et al. 2015]* - *Personalization and tailoring [Chantler et al. 2016]* - *Tailoring to user’s capacity and preferences [Rahimi et al. 2015]* |
| - *Outcome expectancy:* Parameters of effectiveness; Proposed mediating variables (Logic model); Outcome expectancy is defined as a person’s estimate that a given behavior will lead to certain outcomes *[Band et al. 2017; Social Cognitive Theory]* | - *Outcome expectancy [Bradbury et al. 2017; Social Cognitive Theory]* - *Outcome expectations [Duff et al. 2018; Walsh et al. 2018a; Social Cognitive Theory]* |
| - *User involvement:* End-users should be involved in assessing the acceptability and usability of a behavioral intervention before scaling it up for further clinical evaluation. This can be done in practice through a multi-phased parallel approach of theoretical, modelling, and exploratory or piloting phases *[MRC Guidance for Developing and Evaluating Complex Interventions]* | - *Target population involvement (cross-cutting theme) [Development and Evaluation Process for mHealth]* |

| **Refutational** |  |
| --- | --- |
|  |  |
| - *Parallel approach:* A parallel approach is proposed in practice by the MRC framework for the development and evaluation of complex interventions. This means combining phases 0-II of their framework into one larger activity to develop understanding of the problem, the intervention, and the evaluation *[MRC Guidance for Developing and Evaluating Complex Interventions]* | - *Stepwise approach:* A stepwise approach is proposed by the MRC framework for the development and evaluation of complex interventions. 0—Preclinical or theoretical (why should this intervention work?); 1—Modelling (how does it work?); 2—Exploratory or pilot trial (optimizing trial measures); 3—Definitive randomized controlled trial; 4—Implementation *[MRC Guidance for Developing and Evaluating Complex Interventions]* |
