# Supplementary material for: Supporting Self-Management of Cardiovascular Diseases Through Remote Monitoring Technologies: Metaethnography Review of Frameworks, Models, and Theories Used in Research and Development
Source: J Med Internet Res. 2020 May 21;22(5):e16157. doi: 10.2196/16157 (PMC7273239; doi:10.2196/16157)
Supplement: Multimedia Appendix 7 [file jmir_v22i5e16157_app7.docx]

Multimedia Appendix 7 – Frameworks and models that informed the system’s development, implementation, or evaluation, and their operationalized key ingredients by included studies

| Name | Project and aim(s) | Operationalized key ingredients |
| --- | --- | --- |
| 5E Usability Approach [63] | *PATHway* [60]: To aggregate system requirements into features across five dimensions of usability. | *Dimensions of usability:* Easy to learn; Effective; Efficient; Engaging; Error. |
| Action research [64, 65] | *SUPPORT HF* [48-50]: To inform the iterative and participatory approach by actively inquiring patients and providers to understand problems and provoke changes. | Unclear operationalization of ingredients. |
| Agile Software Development [66] | *SUPPORT HF* [48, 50]: To inform the iterative and participatory approach through simultaneous design and implementation, encouraging rapid response to requirements. | Unclear operationalization of ingredients. |
| A Practical Guide to Usability Testing [67] | *MyHeart* [45]: To design usability validation according to heuristic rules. | Unclear operationalization of ingredients. |
| Behavior Change Wheel / Capability, Opportunity, Motivation and Behavior (COM-B) model [68, 69] | *HOME BP* [52]: To undertake a behavioral analysis of the components of an intervention.  *MedFit* [58]: To design how the best practice guidance and content will be delivered to the end user [58].  *PATHway* [60, 61]: To guide and encourage full consideration of all intervention options prior to implementation, and allow transparent translation of user requirements into intervention components. | *Sources of behavior:* Automatic motivation; Reflective motivation; Physical capability; Physical opportunity; Social opportunity; Psychological capability.  *Intervention functions:* Education; Enablement; Environmental restructuring; Modelling; Persuasion; Training.  *Policy categories:* Guidelines; Service provision; Social planning. |
| Business Model Canvas^a^ | *HeartMapp* [56]: To use the canvas template to guide interviews seeking to build a minimum viable product of a mobile application. | *Key insights for building a minimum viable product:* Key partners; Key activities; Key resources; Value propositions; Customer relationships; Channels; Customer segments; Cost structure; Revenue streams. |
| Business Research Method [70] | *HeartMapp* [56]: To exploratively test the proof-of-concept system using the Business Model Canvas. | Unclear operationalization of ingredients. |
| Development and Evaluation Process for mHealth [71] | *MedFit* [58]:To guide the development of the mobile app from conceptualization to usability and acceptability testing | *Stages*^b^*:* Conceptualization; Formative research; Pretesting; Pilot study.  *Cross-cutting themes*^c^*:* Theoretical basis; Implementation focus; Target population involvement. |
| Goal Directed Design [72] | *MyHeart* [45]: To provide design principles for the iterative software design process of the study. | Unclear operationalization of ingredients. |
| Holistic Patient Interaction model [73] | *MyHeart* [45]: To model the user interaction with the system across several contexts and interaction loops | *Human-technology interaction*^d^*:* Implicit interaction; Implicit input; Implicit output.  *Context:* Medical context; Patient context; Social and business context. |
| Intervention Mapping [74] | *HeartMapp* [55]: To assist the development of a conceptual framework, program objectives, and a program plan for the intervention. | *Stages*^b^*:* Needs assessment; Proximal program objective matrices; Selection of theory-based methods; Program plan; Adaptation and implementation plan; Evaluation plan. |
| Iterative Design Model [46] | *SMASH* [46]: To iteratively modify, optimize, and refine a prototype before implementation in a proof of concept trial. | *Stages*^b^*:* Problem selecting; Observing; Reframing and accommodating; Solution finding; Converging; Experimenting; Diverging. |
| Iterative Refinement and Patient Participatory Approach [49] | *SUPPORT HF* [48-50]: To iteratively refine a system based on patients’ experience while interacting with it. | *Stages*^b^*:* Understanding patients’ monitoring needs; Development; Remote delivery of system refinements; Evaluation. |
| Iterative Software Design Process [73] | *MyHeart* [45]: To evaluate a system in different maturity stages. | *Stages*^b^*:* Conceptualization; Implementation; Deployment. |
| Iterative Software Development [75] | *MyHeart* [45]: To apply an iterative software design process in the study. | Unclear operationalization of ingredients. |
| Medical Research Council’s (MRC) Guidance for Developing and Evaluating Complex Interventions [76-78] | *HOME BP* [52]: To construct a theory-based logic model drawing on the latest evidence.  *CHF PSMS* [47]: To collect and analyze evidence during the development phase of an intervention.  *SUPPORT HF* [50]: To involve end users in assessing the acceptability and usability of an intervention before scaling it up.  *SMASH* [46]: To inform an iterative design model. | *Stages*^b^*:* Preclinical or theoretical; Modelling; Exploratory or pilot trial.  *Development/Modelling stage*^b^*:* Modelling process and outcomes.  *Approaches to development:* Stepwise approach (on paper); Parallel approach (in practice). *User involvement.* |
| Multi-Phase Optimization Strategy [79] | *SMASH* [46]: To inform the iterative design model applied in the study. | Unclear operationalization of ingredients. |
| Patient Work Lens for Consumer-facing Health [80] | *Engage* [57]: To highlight the broader context in which patient health-related work occurs and design the system to align with it. | *Design perspectives*^e^*:* Biomedical lens; Personal skills and behavior lens; Patient work lens.  *Type of technologies:* Clinical technologies; Consumer technologies; Collaborative technologies.  *Patient work lens:* Patient; Patient’s work system; Patient’s work activity. |
| Person-Based Approach [81, 82] | *HOME BP* [52, 53]: To generate a deep understanding of the intervention users through iterative use of qualitative research.  *PATHway* [61]: To adopt an iterative codesign process with extensive user testing. | *Stages*^b^*:* Planning; Design; Development and evaluation of acceptability and feasibility. |
| Realistic Evaluation Framework [83, 84] | *CHF PSMS* [47]: To understand not just whether an intervention works, but for whom, in what circumstances, and how, through the formulation of Context, Mechanisms, and Outcomes (CMO) hypotheses. | *CMO Hypotheses:* Context; Mechanism; Outcome.  *Stages*^b^*:* Formulate the CMO hypotheses; Design a field study to evaluate CMOs; Collect mixed methods data; Analyze data in terms of CMOs; Synthesis of new data into refined CMO hypotheses. |
| Systems Engineering Initiative for Patient Safety 2.0 [85] | *Engage* [57]: To understand, from a macroergonomic perspective, the patient work system, processes, and outcomes for the design of a health application. | *Work system*^f^*:* Person(s); Tools & technology; Tasks; Organization; Internal environment; External environment.  *Work processes:* Health-related work; Patient work; Professional work; Collaborative professional-patient work.  *Outcomes:* Proximal/distal; Desirable/undesirable; Patient; Professional; Organizational.  *Feedback loop.* |
| The Startup Owner's Manual [86] | *HeartMapp* [56]: To frame the scaling up of a new mobile application. | Unclear operationalization of ingredients. |
| User-Centered Design (Ad hoc) [87, 88]^g^ | *“Mock-up”* [59]: To increase patient engagement with a health mobile app by designing from the user’s point of view. | *Stages*^b^*:* Focus group interview with cardiologists; Surveys and interviews with patients; Mock-up design; Usability test. |
| User-Centered Design [89] | *CHF PSMS* [47]: To integrate users’ opinions in cycles of re-design throughout the development phase. | *Stages*^b^*:* Understanding the work; Understanding the work context; Testing a top level design against your understanding of the work; User testing of more detailed prototypes. |
| User-Centered Design of Consumer-facing Health IT [90] | *Engage* [57]: To ensure better integration of the system into daily life and promote acceptance and use. | *Stages*^b^*:* Study (analysis) phase; Design; Evaluate. |
| User Centered Design (ISO, 1999) [91] | *MyHeart* [45]: To provide design principles for the iterative software design process of the study. | Unclear operationalization of ingredients. |
| Usability Framework [92] | *SUPPORT HF* [50]: An ethnographic approach was adopted in the study by recommendation of this framework. | *Indirect method for usability testing:* Ethnography. |
| ^a^The Business Model Canvas had no clear reference provided by the authors of the study, it was interpreted that the choice of the canvas model was derived from business-oriented approaches that informed the study [70, 86].  ^b^Stages of the research or development process.  ^c^Themes that should be maintained throughout the entire development process [71].  ^d^Human-technology/computer interaction is the interaction of a human with the environment and with artifacts which is aimed to accomplish a goal. The idea of implicit interaction (input-output) is that a system can perceive the users interaction with the physical environment and the situation in which an action takes place, without explicit “dialog” from the user [73].  ^e^Design perspectives or lenses target different aspects of patients and their environment, producing different insights into patients’ lives. Insights from different lenses lead to different consumer health informatics design opportunities [80].  ^f^The sociotechnical work system is a structure-process-outcome model where any number of components can interact simultaneously, at a moment in time, to shape performance processes and outcomes. The feedback loop represents intended and unintended adaptions of the work system [85].  ^g^This approach had no clear references and thus was considered a purposely-tailored user-centered design derived from the cited background literature in the introduction and discussion of the paper. | | |
