# Supplementary material for: Supporting Self-Management of Cardiovascular Diseases Through Remote Monitoring Technologies: Metaethnography Review of Frameworks, Models, and Theories Used in Research and Development
Source: J Med Internet Res. 2020 May 21;22(5):e16157. doi: 10.2196/16157 (PMC7273239; doi:10.2196/16157)
Supplement: Multimedia Appendix 8 [file jmir_v22i5e16157_app8.docx]

Multimedia Appendix 8 – Theoretical models that informed the system’s content, and their operationalized key ingredients by included studies

| Name | Project and aim(s) | Operationalized key ingredients |
| --- | --- | --- |
| Cognitive Load Theory [93] | *HeartMapp* [55]: To inform the educational feature of a health mobile app. | Unclear operationalization of ingredients. |
| Cognitive Theory of Multimedia Learning [94] | *HeartMapp* [55]: To inform the educational feature of a health mobile app. | Unclear operationalization of ingredients. |
| Common-Sense Model of Self-Regulation [95] | *HOME BP* [52]: To inform a theory-based intervention logic model that includes determinants of target behaviors. | *Beliefs:* Illness beliefs; treatment beliefs. |
| Congratulate, Ask, Reassure, Encourage Approach [53] | *HOME BP* [53]: To facilitate guidelines to health care practitioners for provision of patient-centered support within an online intervention. | *Guidelines for patient-centered care to support online interventions:* Congratulate; Ask; Reassure; Encourage. |
| Control Theory Framework for Personality-Social, Clinical, and Health Psychology [96] | *CHF PSMS* [47]: To provide theory-based mechanisms for the design an intervention. | *Self-monitoring* |
| Domestication of Technology Theory [97, 98] | *SUPPORT HF* [50]: To describe and analyze the processes of acceptance, rejection, and use of a technology. | *Dimensions of domestication of technology^a^:* Appropriation; Objectification; Incorporation; Conversion. |
| Information, Motivation, Behavioral skills model [99] | *HeartMapp* [54, 55]: To inform the design of the intervention through key determinants for long-term behavior. | *Psychological determinants:* Information; Motivation; Behavioral skills. |
| Instructional Design Approach utilizing a Pedagogical Agent [100] | *HeartMapp* [55]: To inform the educational feature of a health mobile app. | Unclear operationalization of ingredients. |
| Multidimensional Framework For Patient And Family Engagement In Health And Health [113] | *HeartMapp* [54, 55]: To inform the design of the intervention through key mediators for targeted and persistent behavioral change. | *Patient engagement* |
| Normalization Process Theory [101-103] | *HOME BP* [52]: To undertake a behavioral analysis of the components of an intervention. | *Normalization components^b^:* Coherence; Cognitive participation; Collective action; Reflexive monitoring.  *Coherence:* Communal specification; Differentiation; Individual specification; Internalization.  *Cognitive participation:* Activation; Enrolment; Initiation; Legitimation.  *Collective action:* Contextual integration; Interactional workability; Relational integration; Skillset workability.  *Reflexive monitoring:* Communal appraisal; Individual appraisal; Reconfiguration; Systematization. |
| Problem Based Learning [104] | *HeartMapp* [55]: To inform the educational feature of a health mobile app. | Unclear operationalization of ingredients. |
| Self-Determination Theory [105, 106] | *HOME BP* [53]: To inform the design of patient-centered care guidelines to support online interventions.  *SMASH* [46]: To design the intervention program to enhance self-efficacy and intrinsic motivation for sustained behavioral change. | *Types of motivation:* Autonomous regulation; Intrinsic motivation.  *Basic psychological needs:* Need for autonomy; Need for competence; Need for relatedness. Autonomous motivation. |
| Social Cognitive Theory [107-109] | *HOME BP* [52, 53]: To inform a theory-based intervention logic model that includes determinants of target behaviors.  *MedFit* [58]: To design how the best practice guidance and content will be delivered to the end user.  *PATHway* [60]: To understand the complexities of initiating health behavior change. | *Psychological determinants:* Knowledge; Self-efficacy; Outcome expectation; Goals; Perceived environmental impediments and facilitators. |
| Social Ecological Model^c^ | *PATHway* [61]: To ensure that the views and opinions of the full health ecosystem were included. | *Ecosystem levels:* Individual level; Interpersonal level; Community level; Organizational level; Policy level. |
| Technology Acceptance Model [110] | *SUPPORT HF* [48]: To inform the design of a questionnaire to assess technology acceptance. | *Determinants of technology acceptance:* Perceived ease of use; Perceived usefulness; Usage intentions. |
| Unified Theory of Acceptance and Use of Technology model [111, 112] | *MedFit* [58]: To develop a theoretically informed focus group script that would pose questions relating to core constructs identified as impacting the acceptance and use of mobile apps. | *Determinants of technology acceptance:* Behavioral intention; Effort expectancy; Experience; Facilitating conditions; Habit; Hedonic motivation; Performance expectancy; Price value; Social influence. |
| ^a^Domestication of technology refers to the processes of acceptance, rejection, and use of technology by its users [97, 98].  ^b^The normalization process components assist in understanding the practical problems of workability and integration that complex interventions pose [101-103].  ^c^No clear reference was provided by the study for the Social Ecological Model | | |
