# Supplementary material for: Supporting Self-Management of Cardiovascular Diseases Through Remote Monitoring Technologies: Metaethnography Review of Frameworks, Models, and Theories Used in Research and Development
Source: J Med Internet Res. 2020 May 21;22(5):e16157. doi: 10.2196/16157 (PMC7273239; doi:10.2196/16157)
Supplement: Multimedia Appendix 9 [file jmir_v22i5e16157_app9.docx]

Multimedia Appendix 9 – Comparison of included projects and their underlying approaches

Contents

[Highlights of comparison of included projects and their underlying approaches 2](#_Toc18599143)

[Comparison of included projects 3](#_Toc18599144)

[Comparison of underlying approaches through data extraction matrix 6](#_Toc18599145)

[Section I. Study description 7](#_Toc18599146)

[Section II. eHealth intervention 8](#_Toc18599147)

[Section III. Underlying framework, model or theory 9](#_Toc18599148)

[Section IV. Characterization of key ingredients 9](#_Toc18599149)

[Explorative bibliometric analysis of underlying approaches 10](#_Toc18599150)

[Highlights of bibliometric analysis 10](#_Toc18599151)

[Topic labels and example frameworks after four iterations of backward snowballing 11](#_Toc18599152)

[Initial set of studies for snowballing process 12](#_Toc18599153)

[Results after the first iteration of snowballing 13](#_Toc18599154)

[Results after the second iteration of snowballing 14](#_Toc18599155)

[Results after the third iteration of snowballing 15](#_Toc18599156)

[Results after the fourth iteration of snowballing 16](#_Toc18599157)

# Highlights of comparison of included projects and their underlying approaches

**Similarities across the overarching projects were also observed via tabular comparison.** Projects had similar development aims, such as improving self-management behaviors (eg, adherence to medication), delivering adaptive and personalized interventions, as well as facilitating healthcare processes (eg, medication titration). *Main content features* were also similar across interventions, including mostly self-monitoring of symptoms, lifestyle or condition-specific assessments (eg, questionnaires), behavioral change support for lifestyle change (eg, goal setting), educational modules, and in some cases technical support or social connection with peers. In terms of their *mode of delivery and implementation*, most of them made use of familiarization sessions or tutorials to introduce the technology, while some also provided booklets or manuals to assist the users. The time of deployment ranged from “1 to 2 hours” to 50 weeks, which was mostly determined according to their research aims (eg, testing usability or adherence), although in most cases the system was not considered a final product. Overall, the *instructions given to the participants* were to use the system on a daily basis, which mostly involved self-monitoring activities or complying with the prescribed treatment (eg, intake of medication).

**Similarities were searched in the reported data and the context of the studies through the use of the data extraction matrix.** The matrix analysis confirmed that all of the studies reported data of interest about eHealth development (see [element 3e](#_Section_III._Underlying)). *Development* was broadly defined as an iterative process of development of eHealth, entailing activities for pre-design, design, implementation and evaluation. Contextual data about development of the interventions was often perceived as clear. For example, 15 out 17 (88%) studies were characterized as reporting a clear *general aim of development*, while 8 out 17 (47%) also provided *specific objectives of development* (see [element 2c](#_Section_II._eHealth)). Regarding implementation, 11 out of 17 (65%) operationalized frameworks, models, or theories to this end (see [element 3e](#_Section_III._Underlying)). *Implementation* was defined as conducting activities specifically undertaken to realize the adoption, dissemination and long-term use of a product in its intended context. Contextual data about the implementation of interventions was often perceived as unclear. For instance, only two studies were characterized as clearly reporting the *mode of delivery and implementation* of their intervention, including *how participants accessed the intervention* or clarifying the *use parameters* of the intervention and the *instructions given to participants* (see [element 2f](#_Section_II._eHealth)). On evaluation, 12 out of 17 (71%) studies operationalized frameworks, models, or theories focused on this (see [element 3e](#_Section_III._Underlying)). *Evaluation* was conceptualized as conducting either *formative evaluation* (ongoing assessments to improve development) or *summative evaluation* (assessment of the influence, role, impact, and uptake of eHealth). In this case, 13 out of 17 (76%) studies were characterized as reporting their *formative evaluations* in a clear manner (see [element 2h](#_Section_II._eHealth)). Another highlight regarding evaluation was that only two cases measured *process outcomes* (eg, adherence; [element 1j](#_Section_I._Study)), and only one included a clear reporting of results about this ([see element 2i](#_Section_II._eHealth)).

# Comparison of included projects

| Project | Development aim | Devices | Main content features | Mode of delivery and implementation | Type of feedback for patients |
| --- | --- | --- | --- | --- | --- |
| **Heart failure** | | | | | |
| HeartMapp | Engaging patients in self-management (adherence to medication, diet, and PA^a^) | Mobile app; HR^b^ sensor; Bluetooth network | **Patients:** Symptom assessment; Behavior support (walking and deep breathing); Vital signs monitoring (sensors);  Education  **HCP**^c^**:** Access to patients who have given access to daily data; Responding to alerts | Asked patients to navigate for 1-2 hours (usability research: Athilingam et al. 2016); Introductory session; Patients can self- initiate app features or responding to alerts/reminders | **Blended:** Feedback on symptom’s severity (NYHA^d^’s functional classification); Tailored, personalized alerts and reminders; Educational SMS^i^; Bio-feedback (walking and deep breathing); Historical graphical information |
| SUPPORT HF | Adaptive system for remote health monitoring and self-management support | Touch Screen Tablet with app; HR^b^ sensor; BP^e^ monitor; Electronic weighing scale; Pulse oximeter  Bluetooth network; Backend server with log data | **Patients:** Assessment (questionnaires every 3 months); Self-monitoring (sensors); Education;  Technical support; Remote updating (via app store)  **HCP**^c^**:** View patient data; Add notes on medical status or usability; Contact patients | Complementary to routine care;  12-50 weeks deployment; Introductory session; Asked patients to complete daily measurement and symptom diaries for use | **Blended:** Graphical display of personal readings; Two-way messaging with HCP; Alerts in case of deterioration |
| CHF PSMS | Increase symptom awareness, knowledge of condition, and PA | Touch screen computer;  Touch screen mobile device; BP^e^ monitor; Weighing scale; Home router device | **Patients:** Self-monitoring (BP, weight, symptoms); Daily planner / Goal setting; Education | 4 weeks deployment; Introductory session at home; Instruction booklet provided; Asked patients to use system on daily basis | **Automated:** Tailored graphical feedback (self-monitoring and PA^a^) |
| Engage | System that is minimally burdensome, personalized and aligned to patient’s self-care routines | Tablet (high-fi prototype); Backend server with log data | **Patients:** Self-monitoring (manual entry symptoms and behaviors); Education; Goal setting / Action planning  **HCP**^c^**:** View patient data reports | Introductory tutorial; Evaluation session in controlled, laboratory setting | **Blended:** Tailored feedback according to trends in log-data or via HCP communication |
| MyHeart | Improve healthcare efficiency and maximizing cost-benefit rate | Mobile device; HR^b^ sensor; BP^e^ monitor; ECG^f^ sensor; Weighing scale; Bluetooth network; Backend server with clinical interface | **Patients:** Self-monitoring (sensors); Exercise/Walking support (prototype scenario)  **HCP**^c^**:** Portal with overview of patients data | System demonstration (prototype) | **Blended:** Graphical feedback on activity |
| **Hypertension** | | | | | |
| HOME BP | Support BP^e^ self-monitoring, medication titration and healthy behavior change | BP^e^ Monitor;  Website | **Patients:** Self-monitoring (manual entry); Lifestyle change support;  **HCP**^c^**:** Support for prescribing medication titration procedures; Guide to provide behavioral support | 24-48 weeks (RCT^g^ protocol: Band et al. 2016); Face-to-face instruction to BP self-monitor; Online demonstration video for BP monitor; Asked patients to perform daily self-monitoring; Informational website sessions accessible for both patients and HCP^c^ | **Blended:** NICE^h^’s guidelines; Automated and instant feedback after BP^e^ input; Automated e-mail reminders (for patients and HCP^c^) |
| SMASH | Improve medication adherence, minimize clinical inertia, sustained behavior change | Mobile app; BP^e^ monitor; Wirelesss medication tray-monitor; Bluetooth network | **Patients:** Reminders and motivational messages (see feedback)  **HCP**^c^**:** Weekly or bimonthly summaries; Alerts if BP^e^ exceeds safety thresholds | 12 weeks (Proof-of-concept RCT^g^) Introductory session; Written and oral adherence criteria (taking medications within 90 minutes range, BP monitoring every 3 days) | **Blended:** Medication tray reminder signals; Tailored motivational SMS^i^ messages |
| **Cardiovascular diseases** | | | | | |
| PATHway | Empower patients to self-manage risk factors after hospital based CR^j^ | Portable computer; HR^b^/PA^a^ sensor; BP^e^ Monitor; ECG^f^ sensor; Motion sensor camera | **Patients:** Lifestyle assessment; Health behavior change program for risk factors; Tailored exercise program; Social interaction; Technical support  **HCP**^c^**:** Health data management system | Complementary to CR^j^ management; 4 face-to-face familiarization classes; Home-based system accessible when desired to patients (intervention not yet delivered) | **Blended:** ‘Good habits visualization’ based on lifestyle assessment; Feedback on exercise execution (motion sensor camera); Summaries of PA^a^ data (HR^b^/PA^a^ sensor) |
| MedFit | Allow tailored, personalized, remote exercise-based rehabilitation | Mobile app; HR^b^/PA^a^ sensor | **Patients:** Exercise support; Education | Introductory session; Manual and helpline access; 2 weeks deployment | **Automated:** Graphical feedback (PA); SMS notifications |
| ‘Mock-up’ | Design customized service for patients and HCP^c^, and improve its usability | Mobile app (low-fi prototype) | **Patients:** Assessment (health questionnaire and mood and symptoms diary); Self-monitoring (mood and symptoms diary); Health status overview; Daily (prescribed) mission; Education | Not applicable (mock-up) | Not applicable  (mock-up, no detailed description) |
| ^a^PA: Physical activity  ^b^HR: Heart rate  ^c^HCP: Healthcare provider  ^d^NYHA: New York Heart Association  ^e^BP: Blood pressure  ^f^ECG: Electrocardiogram  ^g^RCT: Randomized controlled trial  ^h^NICE: National Institute for Health and Care Excellence  ^i^SMS: Short message service  ^j^CR: Cardiac rehabilitation | | | | | |

# Comparison of underlying approaches through data extraction matrix

**Section IV.** Characterization of key ingredients

**Section III.** Underlying framework, model or theory

**Section III**. Underlying framework, model or theory

**Section I.** Study description

**Section II.** eHealth intervention

| **HeartMapp** | **1** | Athilingam et al. 2016 | - Features and usability assessment of a patient-centered mobile application (HeartMapp) for self-management of heart failure |
| --- | --- | --- | --- |
|  | **2** | Athilingam et al. 2018a | - Intervention Mapping Approach in the Design of an Interactive Mobile Health Application to Improve Self-care in Heart Failure |
|  | **3** | Athilingam 2018b | - Mobile technology to improve heart failure outcomes: A proof of concept paperâ€ |
| **HOME BP** | **4** | Band et al. 2016 | - Home and Online Management and Evaluation of Blood Pressure (HOME BP) digital intervention for self-management of uncontrolled, essential hypertension: a protocol for the randomised controlled HOME BP trial |
|  | **5** | Band et al. 2017 | - Intervention planning for a digital intervention for self-management of hypertension: a theory-, evidence- and person-based approach |
|  | **6** | Bradbury et al. 2017 | - Understanding how primary care practitioners perceive an online intervention for the management of hypertension |
| **SUPPORT HF** | **7** | Chantler et al. 2016 | - Creating connections - the development of a mobile-health monitoring system for heart failure: Qualitative findings from a usability cohort study |
|  | **8** | Rahimi et al. 2015 | - A user-centred home monitoring and self-management system for patients with heart failure: a multicentre cohort study |
|  | **9** | Triantafyllidis et al. 2015 | - A personalised mobile-based home monitoring system for heart failure: The SUPPORT-HF Study |
| **PATHway** | **10** | Walsh et al. 2018a | - The development and codesign of the PATHway intervention: a theory-driven eHealth platform for the self-management of cardiovascular disease |
|  | **11** | Walsh et al. 2018b | - Electronic Health Physical Activity Behavior Change Intervention to Self-Manage Cardiovascular Disease: Qualitative Exploration of Patient and Health Professional Requirements |
| **Mock-up** | **12** | Baek, et al. 2018 | - Enhancing user experience through user study: Design of an mhealth tool for self-management and care engagement of cardiovascular disease patients |
| **CHF PSMS** | **13** | Bartlett et al. 2014 | - The SMART personalised self-management system for congestive heart failure: results of a realist evaluation |
| **MedFit App** | **14** | Duff et al. 2018 | - MedFit app, a behavior-changing, theoretically informed mobile app for patient self-management of cardiovascular disease: User-centered development |
| **SMASH** | **15** | McGillicuddy et al. 2012 | - Invited paper: Facilitating medication adherence and eliminating therapeutic inertia using wireless technology: Proof of concept findings with uncontrolled hypertensives and kidney transplant recipients |
| **Engage** | **16** | Srinivas et al. 2017 | - Human factors analysis, design, and evaluation of engage, a consumer health IT application for geriatric heart failure self-care |
| **MyHeart** | **17** | Villalba et al. 2009 | - Validation Results of the User Interaction in a Heart Failure Management System |

## Section I. Study description

Go back to **Matrix** **Overview**

| **Project** | | **HeartMapp** | | | **HOME BP** | | | **SUPPORT HF** | | | **PATHway** | | **Mock-up** | **CHF PSMS** | **MedFit** | **SMASH** | **Engage** | **MyHeart** |
| --- | --- | --- | --- | --- | --- | --- | --- | --- | --- | --- | --- | --- | --- | --- | --- | --- | --- | --- |
| **Paper** | | *1* | *2* | *3* | *4* | *5* | *6* | *7* | *8* | *9* | *10* | *11* | *12* | *13* | *14* | *15* | *16* | *17* |
| **1g Aim** | i) General study aim | 🗸 | 🗸 | 🗸 | 🗸 | 🗸 | 🗸 | 🗸 | 🗸 | 🗸 | 🗸 | 🗸 | 🗸 | 🗸 | 🗸 | 🗸 | 🗸 | 🗸 |
|  | ii) Research question(s) and study objective(s) | 🗸 | ~ | 🗸 | 🗸 | 🗸 | 🗸 | 🗸 | 🗸 | 🗸 | 🗸 | 🗸 | 🗸 | 🗸 | 🗸 | 🗸 | 🗸 | ~ |
| **1h Design** | i) Study classification | 🗸 |  | 🗸 |  |  | 🗸 | 🗸 | 🗸 |  |  | 🗸 | 🗸 | 🗸 | 🗸 | 🗸 | 🗸 |  |
|  | ii) Setting | 🗸 |  | 🗸 | 🗸 |  | 🗸 | 🗸 | 🗸 |  | 🗸 | 🗸 | 🗸 | ~ | 🗸 | ~ | ~ | 🗸 |
|  | iii) Institutions involved |  |  | 🗸 | 🗸 |  | 🗸 | ~ | ~ |  | ~ | 🗸 | ~ |  | ~ | ~ | ~ |  |
| **1i Participants** | i) Eligibility criteria |  |  | ~ | 🗸 |  |  | 🗸 | 🗸 | 🗸 | 🗸 |  | 🗸 |  | ~ | 🗸 |  |  |
|  | ii) Recruitment procedure | 🗸 |  | 🗸 | 🗸 | ~ | ~ | 🗸 | 🗸 | 🗸 | 🗸 | 🗸 | 🗸 | ~ | ~ | 🗸 | 🗸 |  |
|  | iii) Sample characteristics | 🗸 | ~ | ~ |  | ~ | 🗸 | 🗸 | 🗸 | ~ | ~ | 🗸 | 🗸 |  | 🗸 |  | ~ | ~ |
|  | iv) Computer / Internet literacy | 🗸 |  |  |  |  |  | 🗸 | 🗸 |  |  | 🗸 | 🗸 |  | 🗸 |  | 🗸 |  |
| **1j Study outcomes** | i) Primary outcome(s) | 🗸 | ~ | ~ | 🗸 | 🗸 | 🗸 | 🗸 | 🗸 | 🗸 | 🗸 | 🗸 | 🗸 | 🗸 | 🗸 | ~ | ~ | ~ |
|  | ii) Secondary outcomes(s) |  | ~ |  | 🗸 |  |  |  | 🗸 |  |  |  |  |  |  |  |  |  |
|  | iii) Process outcome(s) |  | ~ |  | 🗸 |  |  |  | 🗸 |  |  |  |  |  |  |  |  |  |
|  | iv) Data collection method(s), tools, and analaysis | 🗸 | ~ | 🗸 | 🗸 | 🗸 | 🗸 | 🗸 | 🗸 | 🗸 | 🗸 | 🗸 | 🗸 | 🗸 | 🗸 | ~ | 🗸 | ~ |
| **Codes: 🗸** = Data element was clearly identifiable; ~ = Data element was partially identifiable or incomplete; (Blank cell) = Data element was not applicable or was not reported | | | | | | | | | | | | | | | | | | |

Go back to **Matrix Overview**

## Section II. eHealth intervention

| **Project** | | **HeartMapp** | | | **HOME BP** | | | **SUPPORT HF** | | | **PATHway** | | **Mock-up** | **CHF PSMS** | **MedFit** | **SMASH** | **Engage** | **MyHeart** |
| --- | --- | --- | --- | --- | --- | --- | --- | --- | --- | --- | --- | --- | --- | --- | --- | --- | --- | --- |
| **Paper** | | *1* | *2* | *3* | *4* | *5* | *6* | *7* | *8* | *9* | *10* | *11* | *12* | *13* | *14* | *15* | *16* | *17* |
| **2b**, i) Developers & sponsors | | ~ | ~ | 🗸 | 🗸 | 🗸 | ~ | 🗸 | 🗸 | 🗸 | ~ | ~ | ~ | ~ | ~ | ~ | 🗸 | 🗸 |
| **2b**, ii) Owners | | ~ | ~ | ~ |  |  |  |  |  |  |  |  |  |  |  |  |  |  |
| **2c Development aim** | i) General aim of development | 🗸 | 🗸 | 🗸 | 🗸 | 🗸 | ~ | 🗸 | 🗸 | 🗸 | 🗸 | 🗸 | ~ | 🗸 | 🗸 | 🗸 | 🗸 | 🗸 |
|  | ii) Specific objectives of development | ~ | 🗸 | ~ | 🗸 | 🗸 | ~ | ~ |  | 🗸 | 🗸 | ~ |  | 🗸 | ~ | ~ | 🗸 | 🗸 |
| **2d Device(s) and main technical functionalities** | | 🗸 | 🗸 |  | ~ | ~ | ~ | 🗸 | 🗸 | 🗸 | 🗸 | 🗸 | ~ | 🗸 | ~ | 🗸 | ~ | 🗸 |
| **2e Main content features** | i) Summary of main content features | 🗸 | 🗸 |  | 🗸 | ~ | ~ | 🗸 | 🗸 | 🗸 | 🗸 | ~ | 🗸 | 🗸 | 🗸 | 🗸 | 🗸 | ~ |
|  | ii) In-depth description of content components | 🗸 | 🗸 |  | 🗸 | 🗸 | ~ | ~ |  | 🗸 | 🗸 |  | ~ | ~ | 🗸 | ~ | 🗸 | ~ |
| **2f Mode of delivery and implementation** | i) How participants accessed the intervention | ~ |  |  | 🗸 |  | 🗸 |  | ~ | 🗸 | 🗸 |  |  | 🗸 | 🗸 | ~ | ~ | ~ |
|  | ii) Use parameters |  | 🗸 |  | 🗸 |  |  | 🗸 | 🗸 |  | 🗸 |  |  |  | 🗸 | ~ | ~ | ~ |
|  | iii) Instructions of use given to participants | ~ |  |  | 🗸 |  |  | 🗸 | 🗸 |  | 🗸 |  |  | 🗸 |  | 🗸 |  |  |
| **2g Feedback** | i) Main description of feedback process and features | 🗸 | 🗸 |  | ~ | 🗸 | ~ | ~ | ~ | ~ | 🗸 |  | ~ |  | ~ | 🗸 | 🗸 | ~ |
|  | ii) Level of human involvement | ~ |  |  | 🗸 | 🗸 | 🗸 | ~ | 🗸 | ~ | 🗸 |  |  | ~ | ~ | ~ | ~ | ~ |
|  | iii) Communication channels | ~ | ~ |  | 🗸 | 🗸 | 🗸 | ~ | ~ |  | 🗸 |  |  |  | ~ | ~ |  |  |
|  | iv) Presentation principles or strategies |  |  |  |  |  |  |  |  |  | 🗸 |  |  |  |  |  | 🗸 |  |
| **2h Development process** | i) Historical summary |  | 🗸 | 🗸 |  | ~ | 🗸 |  | ~ | ~ | ~ |  |  |  |  | 🗸 |  |  |
|  | ii) Formative evaluations | ~ | 🗸 | ~ |  | 🗸 | 🗸 | 🗸 | 🗸 | 🗸 | 🗸 |  | 🗸 | 🗸 | 🗸 | 🗸 | 🗸 | 🗸 |
|  | iii) Digital preservation |  |  |  | ~ | 🗸 | 🗸 | ~ |  |  | 🗸 |  |  |  |  |  |  |  |
|  | iv) Published studies or grey literature | 🗸 | 🗸 | 🗸 | 🗸 | 🗸 | 🗸 | 🗸 | 🗸 | 🗸 | 🗸 | 🗸 | 🗸 | 🗸 | 🗸 | 🗸 | 🗸 | 🗸 |
| **2i Intervention results** | i) Results on primary and secondary outcome(s) | 🗸 |  | 🗸 |  | 🗸 | 🗸 | 🗸 | 🗸 | 🗸 | 🗸 | 🗸 | 🗸 | 🗸 | 🗸 | ~ | 🗸 | 🗸 |
|  | ii) Report on process outcome(s) |  |  |  |  |  |  |  | 🗸 |  |  |  |  |  |  |  |  |  |
|  | iii) Report on technical problems or unintended effects |  |  |  |  |  |  | 🗸 | 🗸 | 🗸 |  |  |  |  |  |  |  |  |
|  | iv) Interpretation and principal findings | 🗸 | ~ | 🗸 |  | 🗸 | 🗸 | 🗸 | 🗸 | 🗸 | 🗸 | 🗸 | 🗸 | 🗸 | 🗸 | 🗸 | 🗸 | ~ |

**Codes: 🗸** = Data element was clearly identifiable; ~ = Data element was partially identifiable or incomplete; (Blank cell) = Data element was not applicable or was not reported

Go back to **Matrix Overview**

## Section III. Underlying framework, model or theory

| **Project** | | **HeartMapp** | | | **HOME BP** | | | **SUPPORT HF** | | | **PATHway** | | **Mock-up** | **CHF PSMS** | **MedFit** | **SMASH** | **Engage** | **MyHeart** |
| --- | --- | --- | --- | --- | --- | --- | --- | --- | --- | --- | --- | --- | --- | --- | --- | --- | --- | --- |
| **Paper** | | *1* | *2* | *3* | *4* | *5* | *6* | *7* | *8* | *9* | *10* | *11* | *12* | *13* | *14* | *15* | *16* | *17* |
| **3b Description** | i) Original source(s) referenced by the study author(s) | 🗸 | 🗸 | ~ | 🗸 | 🗸 | 🗸 | 🗸 | 🗸 | 🗸 | 🗸 | 🗸 | ~ | 🗸 | 🗸 | 🗸 | 🗸 | 🗸 |
|  | ii) General description | 🗸 | 🗸 | ~ | 🗸 | 🗸 | 🗸 | 🗸 | ~ | 🗸 | 🗸 | 🗸 | ~ | 🗸 | 🗸 | 🗸 | 🗸 | 🗸 |
|  | iii) Key framework, model or theory ingredients | ~ | 🗸 | ~ | 🗸 | 🗸 | 🗸 | 🗸 | ~ | 🗸 | 🗸 | 🗸 | ~ | 🗸 | 🗸 | 🗸 | 🗸 | 🗸 |
|  | iv) Visual representation | ~ | 🗸 | 🗸 |  | 🗸 |  | ~ | ~ | 🗸 | 🗸 | 🗸 | ~ | 🗸 | 🗸 | 🗸 | 🗸 | 🗸 |
| **3c Operationalization** | | 🗸 | 🗸 | ~ |  | 🗸 | 🗸 | 🗸 |  | 🗸 | 🗸 | 🗸 | 🗸 | 🗸 | 🗸 | 🗸 | 🗸 | 🗸 |
| **3d Categorization** | Framework | 1 | 2 |  | 1 | 3 | 1 | 5 | 3 | 3 | 2 |  |  | 3 | 2 |  | 3 | 1 |
|  | Model |  |  | 1 |  | 2 | 1 |  | 1 |  | 2 | 2 | 1 |  |  | 1 |  | 1 |
|  | Theory | 1 | 1 |  |  | 2 | 1 | 1 |  |  | 1 |  |  | 1 | 2 | 1 |  |  |
| **3e Approach to eHealth** | Development | 2 | 3 | 1 | 1 | 7 | 2 | 5 | 3 | 3 | 4 | 2 | 1 | 4 | 4 | 2 | 3 | 2 |
|  | Implementation |  | 1 |  |  | 1 | 3 | 1 | 3 | 3 | 2 |  |  | 2 | 2 | 1 |  | 1 |
|  | Evaluation |  | 1 |  |  |  | 1 | 2 | 4 | 3 | 2 |  | 1 | 2 | 1 | 1 | 1 | 1 |
|  | | | | | | | | | | | | | | | | | | |

## Section IV. Characterization of key ingredients

| **Project** | | **HeartMapp** | | | **HOME BP** | | | **SUPPORT HF** | | | **PATHway** | | **Mock-up** | **CHF PSMS** | **MedFit** | **SMASH** | **Engage** | **MyHeart** |
| --- | --- | --- | --- | --- | --- | --- | --- | --- | --- | --- | --- | --- | --- | --- | --- | --- | --- | --- |
| **Paper** | | *1* | *2* | *3* | *4* | *5* | *6* | *7* | *8* | *9* | *10* | *11* | *12* | *13* | *14* | *15* | *16* | *17* |
| **4a CeHRes principles** | Participatory development |  | 1 |  |  | 1 | 1 | 5 | 3 | 3 | 1 | 1 | 1 | 1 | 1 | 1 | 1 | 1 |
|  | Creation of new ecosystems for improving health and healthcare |  | 1 |  |  | 1 | 1 |  |  | 3 | 1 | 1 |  | 1 | 2 |  |  | 1 |
|  | Intertwined with implementation |  | 1 | 1 |  | 1 | 1 | 5 | 3 | 3 | 1 |  |  | 2 | 1 | 1 |  | 1 |
|  | Persuasive technology design |  | 1 |  |  | 2 | 1 |  |  |  | 1 |  |  | 1 | 2 |  |  |  |
|  | Continuous evaluation cycles |  | 1 | 1 |  |  | 1 | 5 | 3 | 3 | 1 |  | 1 | 2 | 1 | 1 | 1 | 1 |
| **4b Effectiveness** | Behavior change | 2 | 2 |  |  | 3 | 1 |  |  |  | 3 | 1 |  | 2 | 1 | 1 | 2 |  |
|  | Technology adoption |  |  | 1 |  | 1 | 2 | 1 | 1 |  | 1 |  |  | 1 | 1 |  | 1 | 1 |
|  | Health-related outcomes | 2 | ~ |  |  | 1 | ~ |  |  |  | 2 |  |  | 1 | 1 |  | 1 | 1 |
| **Codes: 🗸** = Data element was clearly identifiable; ~ = Data element was partially identifiable or incomplete; (Blank cell) = Data element was not applicable or was not reported  **Numbers:** In section III, numbers quantify frameworks, models, or theories identified per paper. For example, Paper 1 includes data about one framework and one theory, both are applied to development. In section IV, numbers quantify how many frameworks, models, or theories include certain key ingredients. For example, Paper 1 includes two frameworks, models, or theories that include key ingredients addressing behavior change and two that include key ingredients about health-related outcomes. Numbers shouldn’t be read or added row-wise, for instance, because the framework identified in Paper 1 is the same one identified in Paper 2. | | | | | | | | | | | | | | | | | | |

# Explorative bibliometric analysis of underlying approaches

## Highlights of bibliometric analysis

**Backward snowballing was performed on the references of cited underlying approaches until no further records were deemed relevant to include.** The importance of each article can be determined using the number of times it was cited by the set of studies considered (*InDegree*) and the *PageRank*, which is a recursive importance measure considering the relative importance of the studies citing other studies. *Topic modelling* was used to identify common logical topics across multiple studies based on their keywords and abstracts.

**The bibliometric analysis explored relations across the identified underlying approaches.** The initial set of studies for the snowball process was defined as the overlap between the original sources of underlying approaches identified through the manual review process. To conduct this process, the initial set of was also restricted to those references identified in the Web of Science database by DOI (n=22; [see initial set of studies](#_Initial_set_of)). External references were added to the initial set and the process was repeated. To make sure that the process converged, the requirement for the number of references to an external study increased every step, starting from 2 at the initial step. Through topic modelling, four topics were identified using *Latent Dirichlet Allocation (LDA)* performed on the set of studies obtained after each step in the snowballing process. The tables below show the results of topic modelling at the four iteration of backward snowballing. The results of all iterations along with descriptive graphs about the most popular publications, productive and most cited authors for each step are shown [below](#_Results_after_first).

**Topic modelling revealed the importance of the *Medical Research Council’s (MRC) Guidance for Developing and Evaluating Complex Interventions* in the selected literature (Σ_InDegree_=78 after four iterations).** This influence was also observed during data extraction, as the MRC guidelines were often referred as informing underlying approaches. Additionally, the *Normalization Process Theory*, the *Information, Motivation, Behavioral skills mode*l, *Self-Determination Theory*, and *Agile Software Development* were also frameworks with a degree of importance that appeared in the included literature.

**Topic modelling revealed “behavior change” and “health care” as separate themes in the literature, constituted by different types of frameworks.** This contrast was also observed in the underlying approaches used by included studies. For example, by the frequent use of a *behavioral perspective* such as the Behavior Change Wheel, in contrast to the Systems Engineering Initiative for Patient Safety 2.0 which promotes a broader perspective of the health care system.

**Finally, topic modelling also identified “intervention evaluation and reporting” as a unique theme, exemplified by several works related to the Consolidated Standards of Reporting Trials (CONSORT).** However, this topic was not visible during data extraction as only one study made use of one reporting guidance, the Template for Intervention Description and Replication (TIDieR). This topic could be arguably explained by the wide discussion on the use of effectiveness trials and a potential paradigm shift to measure outcomes rather than just effects.

## Topic labels and example frameworks after four iterations of backward snowballing

| Topic label^a^ (Importance measures) | Keywords | Example framework |
| --- | --- | --- |
| Intervention development (Σ_InDegree_ = 78; *M*_PageRank_ = 0,0002372) | health; develop; evalu; implement; process; practic; model; research; theory; complex | Medical Research Council’s (MRC) Guidance for Developing and Evaluating Complex Interventions  Normalization Process Theory  Information, Motivation, Behavioral skills model  Self-Determination Theory  Agile Software Development |
| Behavior change (Σ_InDegree_ = 73; *M*_PageRank_ = 0,0002390) | intervent; effect; chang; behavior; behaviour; outcom; techniqu; method; activ; weight | Behavior Change Wheel |
| Intervention evaluation and reporting (Σ_InDegree_ = 55; *M*_PageRank_ = 0,0002424) | trial; report; improv; evid; control; consort; result; statement; studi; randomis | Consolidated Standards of Reporting Trials (CONSORT) |
| Health care (Σ_InDegree_ = 19; *M*_PageRank_ = 0,0002282) | care; patient; system; support; work; approach; design; condit; health; factor | Systems Engineering Initiative for Patient Safety 2.0  Multidimensional Framework For Patient And Family Engagement In Health And Health  Person-Based Approach |
| ^a^Topic labels were added by the main reviewer after relating each reference to a framework, model, or theory. This was done either by observing the title, abstract, or authors of a study. | | |

## Initial set of studies for snowballing process

| Document title | | | DOI | |
| --- | --- | --- | --- | --- |
| 1. Designing and evaluating complex interventions to improve health care | | | 10.1136/BMJ.39108.379965.BE | |
| 1. Developing and evaluating complex interventions: the new Medical Research Council guidance | | | 10.1136/BMJ.A1655 | |
| 1. The Person-Based Approach to Intervention Development: Application to Digital Health-Related Behavior Change Interventions | | | 10.2196/JMIR.4055 | |
| 1. SEIPS 2.0: a human factors framework for studying and improving the work of healthcare professionals and patients | | | 10.1080/00140139.2013.838643 | |
| 1. Patient And Family Engagement: A Framework For Understanding The Elements And Developing Interventions And Policies | | | 10.1377/HLTHAFF.2012.1133 | |
| 1. The behaviour change wheel: A new method for characterising and designing behaviour change interventions | | | 10.1186/1748-5908-6-42 | |
| 1. A rational model for assessing and evaluating complex interventions in health care | | | 10.1186/1472-6963-6-86 | |
| 1. Agile software development: Adaptive systems principles and best practices | | | 10.1201/1078.10580530/46108.23.3.20060601/93704.3 | |
| 1. Understanding how primary care practitioners perceive an online intervention for the management of hypertension | | | 10.1186/S12911-016-0397-X | |
| 1. Designing, Implementing, and Evaluating Mobile Health Technologies for Managing Chronic Conditions in Older Adults: A Scoping Review | | | 10.2196/MHEALTH.5127 | |
| 1. A personalised mobile-based home monitoring system for heart failure: The SUPPORT-HF Study | | | 10.1016/J.IJMEDINF.2015.05.003 | |
| 1. Transforming consumer health informatics through a patient work framework: connecting patients to context | | | 10.1136/AMIAJNL-2014-002826 | |
| 1. Developing and evaluating complex interventions: The new Medical Research Council guidance | | | 10.1016/J.IJNURSTU.2012.09.010 | |
| 1. Is realist evaluation keeping its promise? A review of published empirical studies in the field of health systems research | | | 10.1177/1356389012442444 | |
| 1. A Development and Evaluation Process for mHealth Interventions: Examples From New Zealand | | | 10.1080/10810730.2011.649103 | |
| 1. Normalisation process theory: a framework for developing, evaluating and implementing complex interventions | | | 10.1186/1741-7015-8-63 | |
| 1. Process evaluation for complex interventions in primary care: understanding trials using the normalization process model | | | 10.1186/1471-2296-8-42 | |
| 1. A strategy for optimizing and evaluating behavioral interventions | | | 10.1207/S15324796ABM3001_8 | |
| 1. Health promotion by social cognitive means | | | 10.1177/1090198104263660 | |
| 1. Heuristics for iterative software development | | | 10.1109/52.922728 | |
| 1. Cognitive principles of multimedia learning: The role of modality and contiguity | | | 10.1037/0022-0663.91.2.358 | |
| 1. User acceptance of computer-technology - a comparison of 2 theoretical-models | | | 10.1287/MNSC.35.8.982 | |
| Results after the first iteration of snowballing \|Topic.1 \|Topic.2 \|Topic.3 \|Topic.4 \|  \|:-------\|:---------\|:----------\|:----------\|  \|health \|design \|develop \|intervent \|  \|patient \|chang \|approach \|evalu \|  \|system \|care \|home \|process \|  \|studi \|framework \|personbas \|implement \|  \|work \|develop \|result \|model \|  \|engag \|behaviour \|user \|complex \|  \|discuss \|mhealth \|behavior \|practic \|  \|factor \|condit \|view \|potenti \|  \|applic \|research \|accept \|trial \|  \|human \|technolog \|practition \|understand \| | | 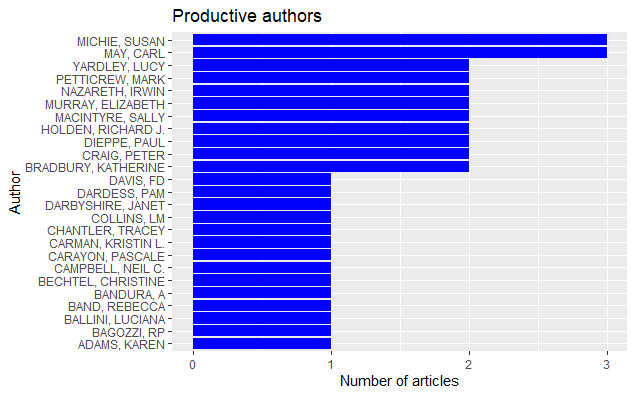 | |  |
| 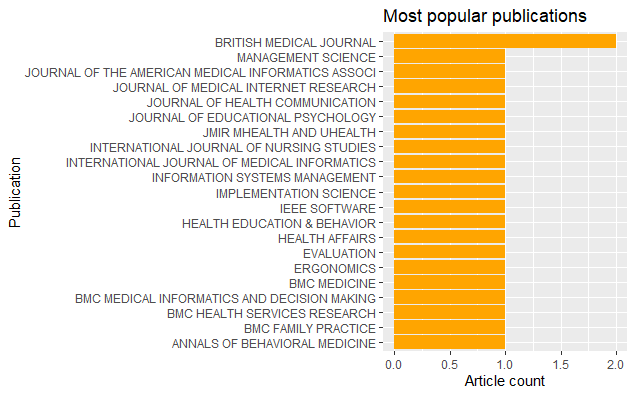 | | 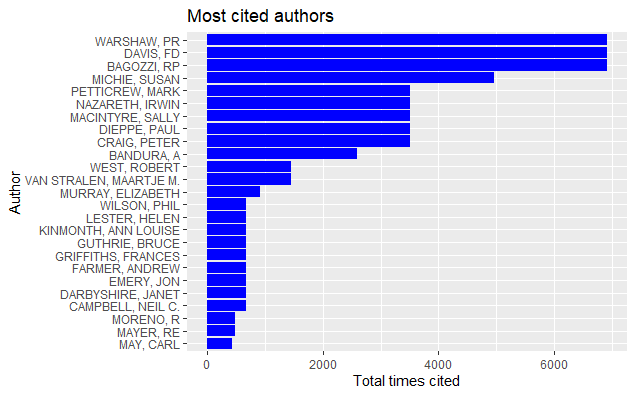 | |  |
| Results after the second iteration of snowballing \|Topic.1 \|Topic.2 \|Topic.3 \|Topic.4 \|  \|:---------\|:---------\|:---------\|:-------\|  \|health \|patient \|intervent \|care \|  \|develop \|system \|trial \|effect \|  \|evalu \|approach \|chang \|studi \|  \|implement \|framework \|behaviour \|support \|  \|process \|work \|design \|control \|  \|practic \|paper \|techniqu \|group \|  \|research \|design \|report \|measur \|  \|complex \|healthcar \|behavior \|result \|  \|theori \|human \|outcom \|death \|  \|model \|home \|applic \|month \| | | 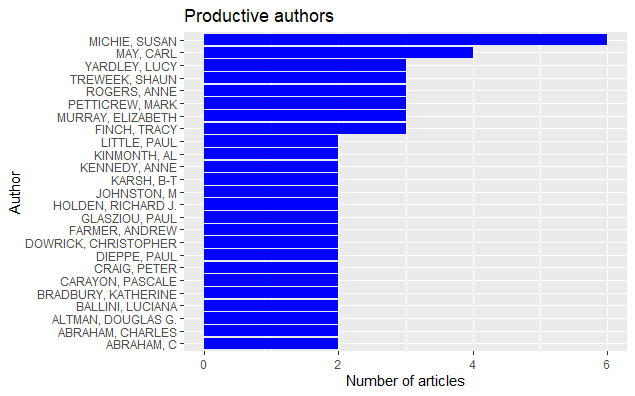 | |  |
| 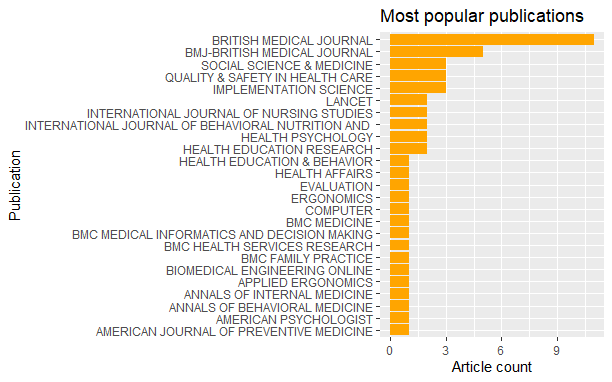 | | 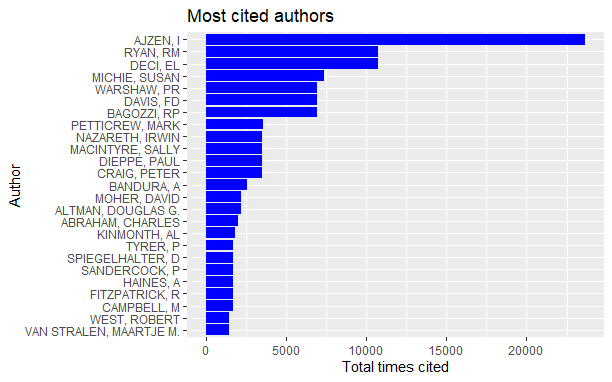 | |  |
| Results after the third iteration of snowballing \|Topic.1 \|Topic.2 \|Topic.3 \|Topic.4 \|  \|:---------\|:---------\|:---------\|:-------\|  \|effect \|patient \|intervent \|trial \|  \|studi \|health \|develop \|design \|  \|context \|implement \|evalu \|report \|  \|increas \|practic \|chang \|improv \|  \|activ \|process \|research \|result \|  \|weight \|system \|method \|care \|  \|health \|model \|theori \|control \|  \|measur \|complex \|behaviour \|evid \|  \|physic \|care \|review \|outcom \|  \|communiti \|work \|health \|support \| | | 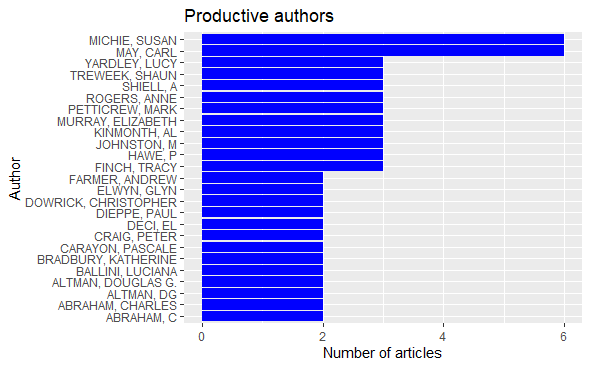 | |  |
| 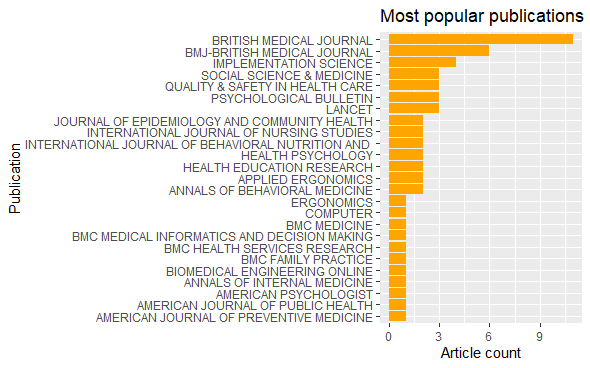 | | 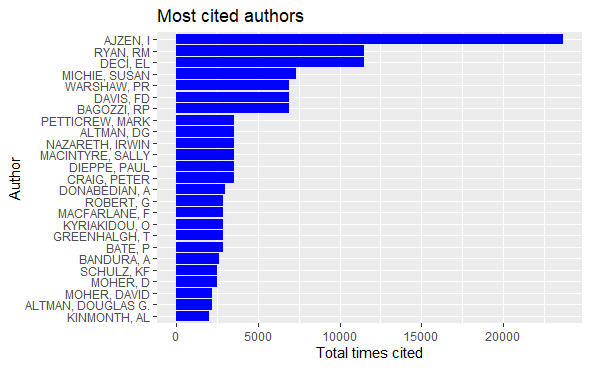 | |  |
| Results after the fourth iteration of snowballing \|Topic.1 \|Topic.2 \|Topic.3 \|Topic.4 \|  \|:---------\|:---------\|:---------\|:--------\|  \|trial \|intervent \|health \|care \|  \|report \|effect \|develop \|patient \|  \|improv \|chang \|evalu \|system \|  \|evid \|behavior \|implement \|support \|  \|control \|behaviour \|process \|work \|  \|consort \|outcom \|practic \|approach \|  \|result \|techniqu \|model \|design \|  \|statement \|method \|research \|condit \|  \|studi \|activ \|theori \|health \|  \|randomis \|weight \|complex \|factor \| | 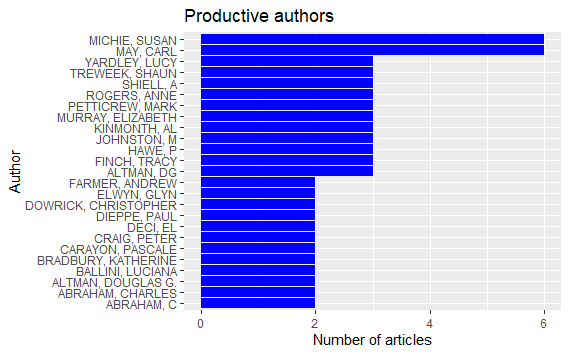 | | |  |
| 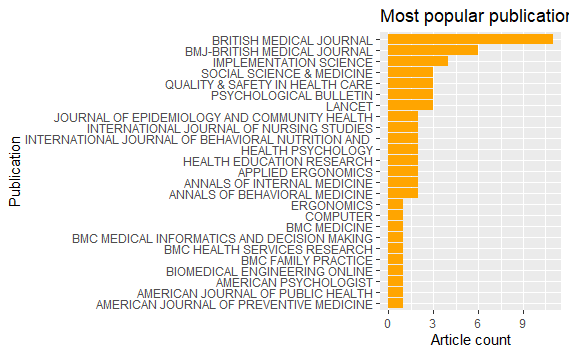 | 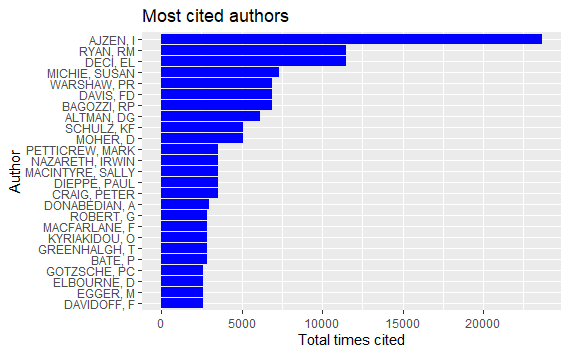 | | |  |
