# Supplementary material for: Supporting Self-Management of Cardiovascular Diseases Through Remote Monitoring Technologies: Metaethnography Review of Frameworks, Models, and Theories Used in Research and Development
Source: J Med Internet Res. 2020 May 21;22(5):e16157. doi: 10.2196/16157 (PMC7273239; doi:10.2196/16157)
Supplement: Multimedia Appendix 10 [file jmir_v22i5e16157_app10.docx]

Multimedia Appendix 10 – Perceived level of clarity and extent of the reported data related to development and design in included articles and projects^a^

|  | **Development aim^b^** | | **Main content features^b^** | | **Mode of delivery and implementation^b^** | | | **Development process^a^** | |
| --- | --- | --- | --- | --- | --- | --- | --- | --- | --- |
| Article | General aim of development^b^ | Specific objectives of development^b^ | Summary of main content features^b^ | In-depth description of content components^b^ | How participants accessed the intervention^b^ | Use parameters^b^ | Instructions of use given to participants^b^ | Historical summary^b^ | Formative evaluations^b^ |
| **HeartMapp** |  |  |  |  |  |  |  |  |  |
| [54] | **🗸** | ~ | **🗸** | **🗸** | ~ |  | ~ |  | ~ |
| [55] | **🗸** | **🗸** | **🗸** | **🗸** |  | **🗸** |  | **🗸** | **🗸** |
| [56] | **🗸** | ~ |  |  |  |  |  | **🗸** | ~ |
| **HOME BP** |  |  |  |  |  |  |  |  |  |
| [51] | **🗸** | **🗸** | **🗸** | **🗸** | **🗸** | **🗸** | **🗸** |  |  |
| [52] | **🗸** | **🗸** | ~ | **🗸** |  |  |  | ~ | **🗸** |
| [53] | ~ | ~ | ~ | ~ | **🗸** |  |  | **🗸** | **🗸** |
| **SUPPORT HF** |  |  |  |  |  |  |  |  |  |
| [50] | **🗸** | ~ | **🗸** | ~ |  | **🗸** | **🗸** |  | **🗸** |
| [48] | **🗸** |  | **🗸** |  | ~ | **🗸** | **🗸** | ~ | **🗸** |
| [49] | **🗸** | **🗸** | **🗸** | **🗸** | **🗸** |  |  | ~ | **🗸** |
| **PATHway** |  |  |  |  |  |  |  |  |  |
| [60] | **🗸** | **🗸** | **🗸** | **🗸** | **🗸** | **🗸** | **🗸** | ~ | **🗸** |
| [61] | **🗸** | ~ | ~ |  |  |  |  |  |  |
| **Mock-up** |  |  |  |  |  |  |  |  |  |
| [59] | ~ |  | **🗸** | ~ |  |  |  |  | **🗸** |
| **CHF PSMS** |  |  |  |  |  |  |  |  |  |
| [47] | **🗸** | **🗸** | **🗸** | ~ | **🗸** |  | **🗸** |  | **🗸** |
| **MedFit** |  |  |  |  |  |  |  |  |  |
| [58] | **🗸** | ~ | **🗸** | **🗸** | **🗸** | **🗸** |  |  | **🗸** |
| **SMASH** |  |  |  |  |  |  |  |  |  |
| [46] | **🗸** | ~ | **🗸** | ~ | ~ | ~ | **🗸** | **🗸** | **🗸** |
| **Engage** |  |  |  |  |  |  |  |  |  |
| [57] | **🗸** | **🗸** | **🗸** | **🗸** | ~ | ~ |  |  | **🗸** |
| **MyHeart** |  |  |  |  |  |  |  |  |  |
| [45] | **🗸** | **🗸** | ~ | ~ | ~ | ~ |  |  | **🗸** |
| Total (N=17) | 15 (88%) | 8 (47%) | 12 (71%) | 8 (47%) | 6 (35%) | 6 (35%) | 6 (35%) | 4 (24%) | 13 (76%) |
| ^a^For the full table and highlights of comparison of included projects see Multimedia Appendix 6  ^b^For definitions of each data element see the data extraction matrix in Multimedia Appendix 1  **🗸** = Data element was clearly identifiable  ~ = Data element was partially identifiable or incomplete  (Blank cell) = Data element was not applicable or was not reported | | | | | | | | | |
