# Supplementary material for: Supporting Self-Management of Cardiovascular Diseases Through Remote Monitoring Technologies: Metaethnography Review of Frameworks, Models, and Theories Used in Research and Development
Source: J Med Internet Res. 2020 May 21;22(5):e16157. doi: 10.2196/16157 (PMC7273239; doi:10.2196/16157)
Supplement: Multimedia Appendix 11 [file jmir_v22i5e16157_app11.pdf]

Multimedia Appendix 11 - Conceptual networks of clustered metaphors

______ Reciprocal translation (=)

______ Refutational translation (<>)

**Grey boxes are themes and organizers for the synthesis**

eHealth is a participatory development process

Line-of-argument (Conceptual network)

PRINCIPLES

Target population involvement (Cross-cutting theme)

The target audience must be involved in order to ensure the intervention is engaging and useful. Input should be appreciated and should be able to be acted upon by altering the intervention accordingly [Development and Evaluation Process for mHealth]

User involvement

End-users should be involved in assessing the acceptability and usability of a behavioral intervention before scaling it up for further clinical evaluation. This can be done in practice through a multi-phased parallel approach of theoretical, modelling, and exploratory or piloting phases [MRC Guidance for Developing and Evaluating Complex Interventions]

OPERATIONALIZATION

Integrating theory-, evidence- and person based approaches

Approach to eHealth; A combination of theory-, evidence- and person based approaches are important to increase the acceptability, engagement with, and effectiveness of an intervention [Band et al. 2017]

SOCIOECOLOGICAL PERSPECTIVE

Diversity of user experiences

Development assumption; Heterogeneity (Heart failure); Technology acceptance; Accounting for the diversity of user experiences in the development and maintenance of eHealth systems is likely to increase the extent of successful uptake and impacts on outcomes for patients and providers. In practice, this highlights the importance of investing time into understanding how different types of users adopt and incorporate digital-health technology into their daily lives [Chantler et al. 2016]

Individual level

The SEM incorporates a wide range of individuals involved at various points of the CVD illness journey through its various levels (eg, individual, interpersonal, organizational, community, and policy) [Social Ecological Model]

Interpersonal level

The SEM incorporates a wide range of individuals involved at various points of the CVD illness journey through its various levels (eg, individual, interpersonal, organizational, community, and policy) [Social Ecological Model]

Community level

The SEM incorporates a wide range of individuals involved at various points of the CVD illness journey through its various levels (eg, individual, interpersonal, organizational, community, and policy) [Social Ecological Model]

Organizational level

The SEM incorporates a wide range of individuals involved at various points of the CVD illness journey through its various levels (eg, individual, interpersonal, organizational, community, and policy) [Social Ecological Model]

Policy level

The SEM incorporates a wide range of individuals involved at various points of the CVD illness journey through its various levels (eg, individual, interpersonal, organizational, community, and policy) [Social Ecological Model]

AIMS

Understanding the work

Common processes in user centred design; Understanding the particular work to be supported by the eHealth system. As with the work context, the data used to do this will come from interviews and observation in the work place. Common types of representations for work are Hierarchical Task Analysis and scenarios [User-centred design]

Understanding the work context

Common processes in user centred design; Understanding the work context involves identifying all the stakeholders and their concerns. eHealth changes the way people work, otherwise there would be no point in introducing them [User-centred design]

Understanding patients' monitoring needs

This cycle includes iterative qualitative and quantitative assessments of the patient's monitoring requirements by the development team. The iterative approach is necessary because the patient's monitoring requirements may change over time (e.g., monitoring devices used, frequency of system use, requested service features) during system run-time operation [Iterative Refinement and Patient Participatory Approach]

PHASES

Study (analysis) phase

Seek to understand the users, their tasks, goals, different aspects of the surrounding environment, and broader contexts [User-centred design of Consumer-facing Health IT]

Planning

Identification of key behavioral issues, needs, and challenges the intervention must address. This stage can be operationalized, for example, through collating and analyzing evidence via a quantitative or qualitative literature synthesis, as well as through primary qualitative research [Person-Based Approach]

Needs assessment

A systematic study of quality of life and health status and those factors that influence them, such as health behavior and environment [Intervention Mapping]

Observing

In this stage the most common issues can be discussed with the target group to identify barriers to conduct the behavior [Iterative Design Model]

Design (PBA)

Creation of guiding principles to help developers summarize and easily refer to features of the intervention identified as central to achieving the intervention objectives. This stage can be operationalized, for example, through theoretical modelling via a theory-based behavioral analysis or the creation of a logic model [Person-Based Approach]

Design (UCD-CHIT)

Design abstract representations or more traditional artifacts such as wireframes or user interface prototypes [User-centred design of Consumer-facing Health IT]

Conceptualization (ISD)

Through initial input and research, a first concept model of the eHealth solution is proposed as several contexts and several interaction loops, for instance, based on the Holistic Patient Interaction Model. The concept is validated with stakeholders via identification of requirements and through prototype design and implementation [Iterative Software Design process]

Development and evaluation of acceptability and feasibility

All intervention components evaluated in detail and optimized from user perspective, as proposed by the Person Based Approach. This stage can be operationalized, for example, by collating and analyzing evidence derived from primary mixed- methods research such as a feasibility study [Person-Based Approach]

Pretesting

In this stage a variety of methods can be used for pretesting early intervention content [Development and Evaluation Process for mHealth]

Evaluate

Evaluate the designs against initial understandings of users and goals [User-centred design of Consumer-facing Health IT]

Implementation

The model is reviewed according to the previous phase and the eHealth solution is assessed in a real environment with real users. The system is validated with stakeholders to update the system requirements and through system design and implementation [Iterative Software Design process]

Pilot Study

In this stage further feedback can be obtained from the target audience on the intervention as it would be delivered in its final form. Other objectives can be to test processes for recruitment, registration, data collection, and technical aspects of delivering content [Development and Evaluation Process for mHealth]

Evaluation

The system's evaluation corresponds to the systematic exploration of usability. In our work, we also consider user engagement with the system as an additional important factor for usability. This can be quantified by recording how often specific system features are used, indicating the extent to which the system is being adopted [31,32] [Iterative Refinement and Patient Participatory Approach]

Deployment

Technical bugs identified in the previous phase are fixed and the design is refine. The complete system is deployed and analyzed again with real users. Conclusions and future work are derived [Iterative Software Design process]

Diverging

In this stage further the intervention is scaled up with a larger and longer feasibility experimental designs, further iterative refinements can then be followed by efficacy and effectiveness trials [Iterative Design Model]

METHODS

Formative Research

Focus groups can be conducted with participants from the target population. For example, to determine how the intended audience uses their mobile phones and how a health-related program can be integrated within that context [Development and Evaluation Process for mHealth]

Surveys and interviews with patients

User research can be conducted through face-to-face interviews and a survey targeting patients with CVD [User-centred design (Ad hoc; Baek et al. 2018)]

Focus group interview with HCP

The focus group interviews method can be used to obtain detailed information on the thoughts, emotions, attitudes, or experiences of participants. It is a suitable model for sharing patient care experiences [User-centred design (Ad hoc; Baek et al. 2018)]

Ethnography

An ethnographic approach can be undertaken as an indirect testing method, for example, to assess usability [Usability Framework]

Testing a top level design against your understanding of the work

Common processes in user centred design; Testing the design using story boards, scenario or cognitive walkthrough [User-centred design]

User testing of more detailed prototypes

Common processes in user centred design; Testing design using paper prototypes or simulations [User-centred design]

eHealth development creates new infrastructures for improving health care, health, and well-being

Line-of-argument (Conceptual network)

OPERATIONALIZATION

Interdisciplinary methods

Development state-of-the-art; Limitations of study/project; There is a growing need to consider adopting methods from other disciplines rather than using deployment–evaluation cycles [54]. Theories, models, and methods to support this approach can be found in engineering and related sectors (e.g., use of factorial or fractionated evaluation designs that have been utilized well within the HCI sphere) [Walsh et al. 2018a]

SOCIOECOLOGICAL PERSPECTIVE

Organizational level

The SEM incorporates a wide range of individuals involved at various points of the CVD illness journey through its various levels (eg, individual, interpersonal, organizational, community, and policy) [Social Ecological Model]

Individual level

The SEM incorporates a wide range of individuals involved at various points of the CVD illness journey through its various levels (eg, individual, interpersonal, organizational, community, and policy) [Social Ecological Model]

Interpersonal level

The SEM incorporates a wide range of individuals involved at various points of the CVD illness journey through its various levels (eg, individual, interpersonal, organizational, community, and policy) [Social Ecological Model]

Community level

The SEM incorporates a wide range of individuals involved at various points of the CVD illness journey through its various levels (eg, individual, interpersonal, organizational, community, and policy) [Social Ecological Model]

Policy level

The SEM incorporates a wide range of individuals involved at various points of the CVD illness journey through its various levels (eg, individual, interpersonal, organizational, community, and policy) [Social Ecological Model]

AIMS

Tailored, personalized, and timely support

eHealth added value; eHealth state-of-the-art; Increasingly widespread access to the internet and mobile phones means that eHealth can be accessible to the majority of patients and can be used to provide information and support at any time the patient needs it. eHealth can empower patients by providing better access to personalized information and support for active involvement in treatment and self-management [Band et al. 2016]

Remote delivery of system refinements

Following delivery of the eHealth technology at the patient's home, remote refinements of the system can be initiated, thereby saving valuable human resources [Iterative Refinement and Patient Participatory Approach]

Facilitating conditions

Determinants of technology acceptance; Consumers' perceptions of the resources and support available to perform a behavior [Unified Theory of Acceptance and Use of Technology model]

Policy categories

Policy categories represent types of decisions made by authorities that help to support and enact an intervention [Behavior Change Wheel/COM-B model]

Service provision

Policy categories; Delivering a service [Behavior Change Wheel/COM-B model]

Guidelines

Policy categories; Creating documents that recommend or mandate practice [Behavior Change Wheel/COM-B model]

Social planning

Policy categories; Designing and or controlling the physical or social environment [Behavior Change Wheel/COM-B model]

PHASES

Conceptualization (DEP)

Stage of development where experts decide on the theoretical basis, review the evidence, and plan the development process. Brainstorming sessions can cover how to translate the theory and evidence into practical methods and techniques [Development and Evaluation Process for mHealth]

METHODS

Program plan

Stage of intervention mapping that includes a description of the scope and sequence of the components of the intervention, the completion of program materials, and protocols for implementation [Intervention Mapping]

eHealth development is intertwined with implementation

Line-of-argument (Conceptual network)

PRINCIPLES

Implementation focus (Cross-cutting theme)

This theme endorses a focus on implementation from the beginning of the development process. There is little point developing an effective intervention if there is no chance of getting it to the target audience after the study [Development and Evaluation Process for mHealth]

AIMS

Key partners

Key insights for building a minimum viable product; The key partners and their motivations to participate in the business model [Business Model Canvas]

Key activities

Key insights for building a minimum viable product; The key activities necessary to implement the business model [Business Model Canvas]

Key resources

Key insights for building a minimum viable product; The key resources needed to make the business model possible [Business Model Canvas]

Value propositions

Key insights for building a minimum viable product; The value proposition of what is offered to the market [Business Model Canvas]

Customer relationships

Key insights for building a minimum viable product; The relationships established with clients [Business Model Canvas]

Channels

Key insights for building a minimum viable product; The communication and distribution channels to reach clients and offer them the value proposition [Business Model Canvas]

Customer segments

Key insights for building a minimum viable product; The segment(s) of clients that are addressed by the value proposition [Business Model Canvas]

Cost structure

Key insights for building a minimum viable product; The cost structure resulting from the business model [Business Model Canvas]

Revenue streams

Key insights for building a minimum viable product; The revenue streams generated by the business model (constituting the revenue model) [Business Model Canvas]

Remote delivery of system refinements

Following delivery of the eHealth technology at the patient's home, remote refinements of the system can be initiated, thereby saving valuable human resources [Iterative Refinement and Patient Participatory Approach]

PHASES

Adaptation and implementation plan

Stage that includes consideration of program sustainability. Matrices are developed with adoption and implementation objectives juxtaposed to personal and external determinants [Intervention Mapping]

Conceptualization (ISD)

Through initial input and research, a first concept model of the eHealth solution is proposed as several contexts and several interaction loops, for instance, based on the Holistic Patient Interaction Model. The concept is validated with stakeholders via identification of requirements and through prototype design and implementation [Iterative Software Design process]

Development and evaluation of acceptability and feasibility

All intervention components evaluated in detail and optimized from user perspective, as proposed by the Person Based Approach. This stage can be operationalized, for example, by collating and analyzing evidence derived from primary mixed- methods research such as a feasibility study [Person-Based Approach]

Implementation

The model is reviewed according to the previous phase and the eHealth solution is assessed in a real environment with real users. The system is validated with stakeholders to update the system requirements and through system design and implementation [Iterative Software Design process]

Diverging

In this stage further the intervention is scaled up with a larger and longer feasibility experimental designs, further iterative refinements can then be followed by efficacy and effectiveness trials [Iterative Design Model]

METHODS

Formative Research

Focus groups can be conducted with participants from the target population. For example, to determine how the intended audience uses their mobile phones and how a health-related program can be integrated within that context [Development and Evaluation Process for mHealth]

Design a field study to evaluate CMO hypotheses

In order to test and generate revised CMO hypotheses, the technology can be deployed in a realistic setting [Realistic Evaluation Framework]

eHealth development integrates theory, evidence, and participatory approaches for persuasive design

Line-of-argument (Conceptual network)

OPERATIONALIZATION

Integrating theory-, evidence- and person based approaches

Approach to eHealth; A combination of theory-, evidence- and person based approaches are important to increase the acceptability, engagement with, and effectiveness of an intervention [Band et al. 2017]

AIMS

Tailoring and personalization

Heterogeneity (CVD); An individual assessment (e.g., on psychological readiness for change) and tailored and personalized features can be useful to achieve health behavior change, to empower patients to make choices and direct them to the most appropriate content for them at a specific time [Walsh et al. 2018a]

Habit

Determinants of technology acceptance; The extent to which people tend to perform behaviors automatically because of learning, also equated with automaticity [Unified Theory of Acceptance and Use of Technology model]

Facilitating conditions

Determinants of technology acceptance; Consumers' perceptions of the resources and support available to perform a behavior [Unified Theory of Acceptance and Use of Technology model]

Hedonic motivation

Determinants of technology acceptance; The fun or pleasure derived from using a technology [Unified Theory of Acceptance and Use of Technology model]

Performance expectancy

Determinants of technology acceptance; The degree to which using a technology will provide benefits to consumers in performing certain activities [Unified Theory of Acceptance and Use of Technology model]

Social influence

Determinants of technology acceptance; The extent to which consumers perceive that important others (e.g., family and friends) believe they should use a particular technology [Unified Theory of Acceptance and Use of Technology model]

Automatic Motivation

Parameters of effectiveness; Target constructs (sources of behaviors); Automatic processes involving emotional reactions, desires (wants and needs), impulses, inhibitions, drive states and reflex responses [Band et al. 2017; Behavior Change Wheel/COM-B model]

Parameters of effectiveness; Sources of behavior; Motivation describes the brain processes that energize and direct behavior and includes both automatic motivation (e.g., habits) and reflective motivation (e.g., cost-benefit decision making) [Walsh et al. 2018a; Behavior Change Wheel/ COM-B model]

eHealth development requires continuous evaluation cycles

Line-of-argument (Conceptual network)

OPERATIONALIZATION

Parallel approach

A parallel approach is proposed in practice by the MRC framework for the development and evaluation of complex interventions. This means combining phases 0-II of their framework into one larger activity to develop understanding of the problem, the intervention, and the evaluation [MRC Guidance for Developing and Evaluating Complex Interventions]

Stepwise approach

A stepwise approach is proposed by the MRC framework for the development and evaluation of complex interventions: 0—Preclinical or theoretical (why should this intervention work?); 1—Modelling (how does it work?); 2—Exploratory or pilot trial (optimizing trial measures); 3—Definitive randomized controlled trial; 4—Implementation [MRC Guidance for Developing and Evaluating Complex Interventions]

Incremental stepped development and evaluation

Theory/model state-of-the-art; Incremental stepped approaches to developing and evaluating behavior change interventions using technology are vital, as per the Medical Research Council (MRC) and behavior change wheel (BCW) frameworks [Walsh et al. 2018a]

Agile vs Waterfall approach

Limitations of study project; A waterfall approach performs analysis, design, and evaluation in relatively long and sequential stages, as opposed to the agile approach which uses many cycles of rapid product development and user feedback [Srinivas et al. 2017]

Interdisciplinary methods

Development state-of-the-art; Limitations of study/project; There is a growing need to consider adopting methods from other disciplines rather than using deployment–evaluation cycles [54]. Theories, models, and methods to support this approach can be found in engineering and related sectors (e.g., use of factorial or fractionated evaluation designs that have been utilized well within the HCI sphere) [Walsh et al. 2018a]

AIMS

Understanding patients' monitoring needs

This cycle includes iterative qualitative and quantitative assessments of the patient's monitoring requirements by the development team. The iterative approach is necessary because the patient's monitoring requirements may change over time (e.g., monitoring devices used, frequency of system use, requested service features) during system run-time operation [Iterative Refinement and Patient Participatory Approach]

PHASES

Development phase

Identifying the evidence base; Identifying/developing theory; Modelling process and outcomes [MRC Guidance for Developing and Evaluating Complex Interventions]

Preclinical or theoretical

A phase that focuses on identifying answer to the question: 'Why should this intervention work?' [MRC Guidance for Developing and Evaluating Complex Interventions]

Study (analysis) phase

Seek to understand the users, their tasks, goals, different aspects of the surrounding environment, and broader contexts [User-centred design of Consumer-facing Health IT]

Solution finding

In this stage a larger number of members of the target group should be involved to identify inconsistencies in the proposed solution(s) [Iterative Design Model]

Conceptualization (DEP)

Stage of development where experts decide on the theoretical basis, review the evidence, and plan the development process. Brainstorming sessions can cover how to translate the theory and evidence into practical methods and techniques [Development and Evaluation Process for mHealth]

Modelling (MRC)

A phase that focuses on identifying answer to the question: 'How does the intervention work?' [MRC Guidance for Developing and Evaluating Complex Interventions]

Evaluation plan

Stage of intervention mapping where variables are defined in a measurable way regarding the decisions about learning and change objectives, methods, strategies, and implementation [Intervention Mapping]

Converging

In this stage the intervention and technology are developed, integrating the insights from all the previous phases [Iterative Design Model]

Conceptualization (ISD)

Through initial input and research, a first concept model of the eHealth solution is proposed as several contexts and several interaction loops, for instance, based on the Holistic Patient Interaction Model. The concept is validated with stakeholders via identification of requirements and through prototype design and implementation [Iterative Software Design process]

Design (UCD-CHIT)

Design abstract representations or more traditional artifacts such as wireframes or user interface prototypes [User-centred design of Consumer-facing Health IT]

Development and evaluation of acceptability and feasibility

All intervention components evaluated in detail and optimized from user perspective, as proposed by the Person Based Approach. This stage can be operationalized, for example, by collating and analyzing evidence derived from primary mixed- methods research such as a feasibility study [Person-Based Approach]

Pretesting

In this stage a variety of methods can be used for pretesting early intervention content [Development and Evaluation Process for mHealth]

Evaluate

Evaluate the designs against initial understandings of users and goals [User-centred design of Consumer-facing Health IT]

Implementation

The model is reviewed according to the previous phase and the eHealth solution is assessed in a real environment with real users. The system is validated with stakeholders to update the system requirements and through system design and implementation [Iterative Software Design process]

Pilot Study

In this stage further feedback can be obtained from the target audience on the intervention as it would be delivered in its final form. Other objectives can be to test processes for recruitment, registration, data collection, and technical aspects of delivering content [Development and Evaluation Process for mHealth]

Experimenting

In this stage the intervention is tested in a small scale, for example through a proof-of-concept RCT [Iterative Design Model]

Exploratory or pilot trial

Optimizing the trial measures of the intervention [MRC Guidance for Developing and Evaluating Complex Interventions]

Evaluation

The system's evaluation corresponds to the systematic exploration of usability. In our work, we also consider user engagement with the system as an additional important factor for usability. This can be quantified by recording how often specific system features are used, indicating the extent to which the system is being adopted [31,32] [Iterative Refinement and Patient Participatory Approach]

Deployment

Technical bugs identified in the previous phase are fixed and the design is refine. The complete system is deployed and analyzed again with real users. Conclusions and future work are derived [Iterative Software Design process]

METHODS

Ethnography

An ethnographic approach can be undertaken as an indirect testing method, for example, to assess usability [Usability Framework]

Analyse data in terms of CMO hypotheses

Through analysis of data the realist evaluators aim to understand the effects on outcomes and identify what works, for whom, in what circumstances [Realistic Evaluation Framework]

Synthesis of new data into refine CMO hypotheses

The CMO hypotheses are revised, rejected, or new ones are proposed according to the analysis of the data [Realistic Evaluation Framework]

Mock-up design

Mock-ups are visualizations that enable users to experience the functions that will be implemented in a technology [User-centred design (Ad hoc; Baek et al. 2018)]

Usability test

Method to evaluate how easily the end user understands, learns, and uses software or an app under specific conditions [User-centred design (Ad hoc; Baek et al. 2018)]

Behavior change

Line-of-argument (Conceptual network)

OPERATIONALIZATION

Self-management (sociotechnical concept)

Self-management is a complex set of processes with multiple goals, embedded in a complex, interacting sociotechnical system, and associated with multiple outcomes. Chronic disease management work is collaborative in that it is performed by a collective group, with agents including patients, informal caregivers, and healthcare professionals. In many cases, however, there are multiple breakdowns and barriers, which can be addressed by technologies or other interventions [Srinivas et al. 2017]

Tailored, personalized, and timely support

eHealth added value; eHealth state-of-the-art; Increasingly widespread access to the internet and mobile phones means that eHealth can be accessible to the majority of patients and can be used to provide information and support at any time the patient needs it. eHealth can empower patients by providing better access to personalized information and support for active involvement in treatment and self-management [Band et al. 2016]

Meaningful feedback

Self-management assumption; For technology to support self-management, the information gathered by the technology must be fed back to the patient in a way that increases understanding and empowerment [Bartlett et al. 2014]

Positive feedback and medication reminders

Development assumption; Self-management assumption; Provision of positive feedback over time and motivational personalized messages can stimulate self-efficacy and autonomous regulation, leading to changes in desired behavioral outcomes [McGillicuddy et al. 2012]

Real-time feedback in self-care

eHealth added value; eHealth state-of-the-art; Self-management state-of-the-art; Real-time feedback enhances self-care by providing patients with timely instructions and support. Harnessing the value of these 'teachable moments' is critical and should be part of well-designed and supported eHealth systems to improve patients' experience of living with their condition [Chantler et al. 2016]

Potential for delivery of behavior change techniques

eHealth added value; eHealth state-of-the-art; eHealth (mobile) solutions deliver many additional behavior change techniques (BCTs) that are not possible, for instance, with standard pedometers, such as goal setting, social support, and cues to action. These new techniques embedded within an eHealth framework may move toward helping to tackle one of the key issues of long term sustained behavior [Duff et al. 2018]

SOCIOTECHNICAL SYSTEMS PERSPECTIVE

Personal skills and behavior lens

Integrates aspects of patients and their environment such as knowledge, cognitive functioning, health literacy, attitude, motivation, readiness to change, behavior, social support, self- efficacy, and self-regulatory skills [Patient Work Lens for Consumer-facing Health]

PSYCHOLOGICAL & BEHAVIORAL DETERMINANTS

Patient activation

Self-management state-of-the-art; The term 'patient activation' is employed to represent patients who are 'more effective managers of their health and healthcare' (Lupton, 2013). However, even these digitally engaged, tech savvy patients may find the obligation of self-care surveillance overwhelming, forcing them to confront their illness, engage in routine actions they would rather avoid, or deal with digital interactions that are tiresome (Oudshoorn, 2012) [Athilingam et al. 2018b]

Self-efficacy

Parameters of effectiveness; Proposed mediating variables (Logic model); The expectation that one can successfully execute the behavior required to produce the outcomes [Band et al. 2017; Social Cognitive Theory]
Parameters of effectiveness; People's judgements of their capabilities to organize and execute courses of action required to attain designated types of performances. It is concerned not with the skills one has but with judgments of what one can do with whatever skills one possesses [Bradbury et al. 2017; Social Cognitive Theory]
Parameters of effectiveness; Perceived self-efficacy that one can exercise control over one's health habits [Walsh et al. 2018a; Social Cognitive Theory]

Perceived self-efficacy

Parameters of effectiveness; Perceived self-efficacy that one can exercise control over one's health habits [Social Cognitive Theory]

Outcome expectancy

Parameters of effectiveness; Proposed mediating variables (Logic model); Outcome expectancy is defined as a person's estimate that a given behavior will lead to certain outcomes [Band et al. 2017; Social Cognitive Theory]
Parameters of effectiveness; A judgement of the likely consequence a behavior will produce [Bradbury et al. 2017; Social Cognitive Theory]

Outcome expectations

Parameters of effectiveness; Outcome expectations about the expected costs and benefits for different health habits [Duff et al. 2018; Walsh et al. 2018a; Social Cognitive Theory]

Goals

Parameters of effectiveness; The health goals people set for themselves and the concrete plans and strategies for realizing them [Duff et al. 2018; Walsh et al. 2018a; Social Cognitive Theory]

Perceived environmental impediments and facilitators

Parameters of effectiveness; The perceived facilitators and social and structural impediments to the desired changes [Duff et al. 2018; Walsh et al. 2018a; Social Cognitive Theory]

Information

Parameters of effectiveness; Information about ones' health and/ or ways to change unhealthy behavior [Athilingam et al. 2016; Information, Motivation, Behavioral skills model]
Parameters of effectiveness; It is the basic knowledge about a medical condition that might include how it develops, its expected course, and effective strategies for its management [Athilingam et al. 2018a; Information, Motivation, Behavioral skills model]

Motivation (IMB)

Parameters of effectiveness; Personal or intrinsic motivation and social motivation from support of family and friends to change the unhealthy behavior [Athilingam et al. 2016; Information, Motivation, Behavioral skills model]
Parameters of effectiveness; It encompasses intrinsic motivation of personal attitudes toward adherence to the behavior and perceived social support for such behavior [Athilingam et al. 2018a; Information, Motivation, Behavioral skills model]

Motivation (COM-B)

Parameters of effectiveness; Sources of behavior; Motivation describes the brain processes that energize and direct behavior and includes both automatic motivation (e.g., habits) and reflective motivation (e.g., cost-benefit decision making) [COM-B model]

Automatic Motivation

Parameters of effectiveness; Target constructs (sources of behaviors); Automatic processes involving emotional reactions, desires (wants and needs), impulses, inhibitions, drive states and reflex responses [Band et al. 2017; Behavior Change Wheel/COM-B model]
Parameters of effectiveness; Sources of behavior; Motivation describes the brain processes that energize and direct behavior and includes both automatic motivation (e.g., habits) and reflective motivation (e.g., cost-benefit decision making) [Walsh et al. 2018a; Behavior Change Wheel/ COM-B model]

Behavioral skills

Parameters of effectiveness; It includes ensuring that the patient has the specific behavioral tools or strategies necessary to perform behaviors such as daily weighing, symptom assessment, and exercise [Athilingam et al. 2016; Information, Motivation, Behavioral skills model]
Parameters of effectiveness; The adoption or acting out of healthy behavior skills that result in desired behavior change [Athilingam et al. 2018a; Information, Motivation, Behavioral skills model]

Psychological Capability

Parameters of effectiveness; Target constructs (sources of behaviors); Knowledge or psychological skills, strength or stamina to engage in the necessary mental processes [Band et al. 2017; Behavior Change Wheel/ COM-B model]
Parameters of effectiveness; Sources of behavior; Capability is the individual's ability to perform a behavior and includes both physical capability (e.g., skills) and psychological capability (e.g., knowledge) [Walsh et al. 2018a, 2018b; Behavior Change Wheel/ COM-B model]

Social Opportunity

Parameters of effectiveness; Target constructs (sources of behaviors); Opportunity afforded by interpersonal [Band et al. 2017; Behavior Change Wheel/COM-B model]
Parameters of effectiveness; Sources of behavior; Opportunity describes the factors that lie outside the individual that facilitate or prompt behavior and includes both physical opportunity (e.g., affordability) and social opportunity (e.g., cultural norms) (e.g., cost-benefit decision making) [Walsh et al. 2018a, 2018b; Behavior Change Wheel/COM-B model]

NORMALIZATION PROCESS COMPONENTS

CH: Communal specification

Target constructs (Logic model); Gaining info about the condition and its management with the help of others; Discussing or altering current management plans with others (family, HCPs) [Normalization Process Theory]

CH: Differentiation

Target constructs (Logic model); Understanding and differentiating between aspects of the illness & treatment – for example, when titration is necessary and when it is not [Normalization Process Theory]

CH: Individual specification

Target constructs (Logic model); Gathering own info about condition, or developing own understanding; Patient chooses to continue with self-monitoring or treatment escalation regime [Normalization Process Theory]

CP: Enrolment

Target constructs (Logic model); Engaging with others (family, HCP) to enable them to support BP management [Normalization Process Theory]

CP: Initiation

Target constructs (Logic model); Organizational aspects of behavior involved in management of blood pressure [Normalization Process Theory]

CP: Legitimation

Target constructs (Logic model); Seeking reassurance from others about appropriateness of management plans [Normalization Process Theory]

CA: Interactional workability

Target constructs (Logic model); Taking medications, medication side-effects, engaging with DI, lifestyle changes – integrating all of these in to everyday life [Normalization Process Theory]

CA: Skillset workability

Target constructs (Logic model); Developing the skills and routine to self-monitor [Normalization Process Theory]

RM: Individual appraisal

Patient chooses to continue with self-monitoring or treatment escalation regime [Normalization Process Theory]

RM: Reconfiguration

Target constructs (Logic model); Altering a set routine (such as medication escalation) when required [Normalization Process Theory]

RM: Systematization

Ways to keep up with newly available treatments [Normalization Process Theory]

PRACTICAL APPLICATIONS

Familiarization phase

Practical applications; For example, through a preliminary phase of face-to-face exercise consultation [PATHway]

Home platform access

Practical applications; For example, the inclusion of a dashboard (front-end visualization of use), technical support, or training videos/manual [PATHway]

Assessment

Practical applications; This feature includes daily patient alerts to check weight and complete a symptom questionnaire [HeartMapp]
Practical applications; Inclusion of different types of assessment, such as lifestyle, behavioral change, and health and fitness [PATHway]

Self-monitoring

Practical applications; Self-monitoring is a theory-based technique. An intervention designer could hypothesize that including self-monitoring (theory-based mechanism) in their intervention will increase physical activity (outcome) [Control Theory Framework for Personality-Social, Clinical, and Health Psychology]

Patient: Self-monitoring & BP entry

Practical applications [HOME BP]

Feedback

Practical applications; Inclusion of different types of feedback, such as:
- Sensor instructions for wearing prior to use
- On-screen visual/verbal avatar instructions prior to exercise
- On-screen positive reinforcement during Exercise (related to exercise accuracy)
- Sensor physiological feedback (i.e. Microsoft Band 2, blood pressure monitor, Zensor)
- End of exercise summary feedback
- Good habits visualization (following lifestyle assessment)
[PATHway]

Feedback on activity

Practical applications; Mechanisms (CMO hypotheses); My review [Touch screen computer]; Review daily walk as 'too hard', 'just right', or 'too easy'; Adjust tomorrow's walking [CHF PSMS]

Feedback on activity performance

Practical applications; Mechanisms (CMO hypotheses) [CHF PSMS]

Behavioral change

Practical applications; Inclusion of features such as health behavior change goal selection, calendar logging of health behavior, or calendar for events/exercise sessions provided by the eHealth system [PATHway]

Goal setting related to user's lifestyle

Practical applications; Mechanisms (CMO hypotheses) [CHF PSMS]

Patient: Self-selected lifestyle changes

Practical applications [HOME BP]

Home exercising

Practical applications; For example, through facilitating a way to practice individual exercise, promoting different physical activity options (e.g., ExerClass/ExerGame/Active lifestyle), and including several features such as multiplayer classes, decision-support for exercise adaptation, or exercise prescription [PATHway]

CHF info/knowledge

Practical application; Enabling patients to read and listen to CHF educational information on daily self-management, medications, diet, and physical activity, managing other chronic diseases or conditions, and managing feelings (emotions) about CHF [HeartMapp]

BLENDED CARE

HCP: Support for lifestyle changes & BP monitoring

Practical applications [HOME BP]

Technology adoption

Line-of-argument (Conceptual network)

OPERATIONALIZATION

Diversity of user experiences

Development assumption; Heterogeneity (Heart failure); Technology acceptance; Accounting for the diversity of user experiences in the development and maintenance of eHealth systems is likely to increase the extent of successful uptake and impacts on outcomes for patients and providers. In practice, this highlights the importance of investing time into understanding how different types of users adopt and incorporate digital-health technology into their daily lives [Chantler et al. 2016]

User-centred design process undertaken

Mechanisms (CMO hypotheses) [CHF PSMS]

Tailored, personalized, and timely support

eHealth added value; eHealth state-of-the-art; Increasingly widespread access to the internet and mobile phones means that eHealth can be accessible to the majority of patients and can be used to provide information and support at any time the patient needs it. eHealth can empower patients by providing better access to personalized information and support for active involvement in treatment and self-management [Band et al. 2016]

Iterative personalization

Development assumption; Evidence gap; Iterative personalization of eHealth services (e.g., remote health monitoring) according to the patient's ongoing healthcare and usability needs is a vital element for successful implementation. Regular adaptation to changing circumstances is therefore necessary for widespread and sustained adoption [Triantafyllidis et al. 2015]

Personalization and tailoring

Development assumption; Heterogeneity (Heart failure); Users might be able to use the system with different levels of involvement and understanding, some taking active control (engagement) of their health whereas others appreciate using the system more passively. This could correlate with their level of digital competency (literacy), which makes personalization desirable [Chantler et al. 2016]

Tailoring to user's capacity and preferences

The need to tailor eHealth (monitoring) systems to user's capacity and preferences should be given sufficient priority during the development stage [Rahimi et al. 2015]

Adaptation to personal routines

Mode of delivery and implementation; Development assumption; Heterogeneity (Heart Failure); The technology must fit intuitively, naturally and in a user-friendly way the user's health and mental status, preferences, and recommended medical protocol [Villalba et al. 2009]

Routine

Development assumption; Heterogeneity (Heart failure); Continuous usage can be strongly associated with patients fitting the self-monitoring of a condition into their daily routine [Chantler et al. 2016]

Acceptability and fit of hardware in everyday life

Mechanisms (CMO hypotheses) [CHF PSMS]

Adaptive and user friendly eHealth

An adaptive and user-friendly monitoring system can enable a wide range of patients to monitor their health status regularly. This can be achieved through automated collection of user interactions with the system (log data) and through remote automatic updates without required input from the patients [Rahimi et al. 2015]

Bidirectional service model

Development assumption; Considering the patients' perspective, advice from doctors or communication with them is proposed as an important factor in a successful eHealth support model [Baek et al. 2018]

Connection

Development assumption; Heterogeneity (Heart failure); A key motivator was the sense of connection an eHealth system provides to a support team (e.g., research group), outweighing, for instance, the ability to view daily readings or health information [Chantler et al. 2016]

Ask

Guidelines for patient-centred care to support online intervention (Blended care); Asking the patients how they are getting on, ask if they have any questions or concerns [CARE model]

CHALLENGES

Overcoming the inertia of disengagement

Development assumption; Design goals should not assume 'baseline levels of engagement' but rather promote it, if even for a relatively short period of time [Srinivas et al. 2017]

Technology knowledge gap

Technology acceptance; There exists a gap between younger and older generations when it comes to familiarization with technology (e.g., smartphones). The gap can become an issue during feasibility testing, and affect confidence to use a technology even after a familiarization process [Duff et al. 2018]

USER INTERACTION PERSPECTIVE

User interaction

Development aim; Development assumption; User interaction with the technology, both through explicit and implicit input [Villalba et al. 2009]

SYSTEM USABILITY

Easy to learn

Dimensions of usability; The easy to learn dimension evaluates how the system supports both initial and long-term use [5E Usability model]

Efficient

Dimensions of usability; The efficient dimension refers to how quickly the task can be done [5E Usability model]

Effective

Dimensions of usability; The effective dimension explores how completely and accurately a user can complete a task [5E Usability model]

Engaging

Dimensions of usability; The engaging dimension highlights how well the interface guides the user intuitively through the task [5E Usability model]

Error

Dimensions of usability; The error tolerant dimension assesses how well a system can avoid user-generated errors and also how the system aids the user in overcoming this error [5E Usability model]

DETERMINANTS & PROCESSES OF TECHNOLOGY ADOPTION

Domestication of technology

The processes of acceptance, rejection and use of technology by its users. Users are seen as social entities and this conceptual model aims to provide a framework for understanding how technology innovations change, and are changed, by their social contexts. For instance, according to the Domestication of Technology Theory this process can be divided in four dimensions: appropriation, objectification, incorporation, and conversion [Domestication of Technology Theory]

Appropriation

Dimensions of domestication of technology; Dimension that addresses questions such as why users are interested in using the system and what motivates them. This can include an interest to take part in an eHealth related study [Domestication of Technology Theory]

Objectification

Dimensions of domestication of technology; Dimension that is about determining what the eHealth system will be used for by the users [Domestication of Technology Theory]

Incorporation

Dimensions of domestication of technology; Dimension that is about the practical hands-on use of the eHealth system by users, and how they continue to evaluate its usefulness becomes more or less integral to their daily lives [Domestication of Technology Theory]

Conversion

Dimensions of domestication of technology; Dimension that deals with the expressed aspirations and continuing interest of users in using an eHealth system [Domestication of Technology Theory]

Behavioral intention

Determinants of technology acceptance; The intention to use a technology [Unified Theory of Acceptance and Use of Technology model]

=

Usage intentions

Determinants of technology adoption; People's technology use can be predicted reasonably well from their intentions [Technology acceptance models]

Performance expectancy

Determinants of technology acceptance; The degree to which using a technology will provide benefits to consumers in performing certain activities [Unified Theory of Acceptance and Use of Technology model]

Perceived usefulness

Determinants of technology adoption; Perceived usefulness is a major determinant of people's intentions to use technology [Technology acceptance models]

Effort expectancy

Determinants of technology acceptance; The degree of ease associated with consumers' use of technology [Unified Theory of Acceptance and Use of Technology model]

Perceived ease of use

Determinants of technology adoption; Perceived ease of use is a significant secondary determinant of people's intentions to use computers [Technology acceptance models]

Experience

Determinants of technology acceptance; The passage of time from the initial use of a technology by an individual [Unified Theory of Acceptance and Use of Technology model]

Habit

Determinants of technology acceptance; The extent to which people tend to perform behaviors automatically because of learning, also equated with automaticity [Unified Theory of Acceptance and Use of Technology model]

Facilitating conditions

Determinants of technology acceptance; Consumers' perceptions of the resources and support available to perform a behavior [Unified Theory of Acceptance and Use of Technology model]

Hedonic motivation

Determinants of technology acceptance; The fun or pleasure derived from using a technology [Unified Theory of Acceptance and Use of Technology model]

Social influence

Determinants of technology acceptance; The extent to which consumers perceive that important others (e.g., family and friends) believe they should use a particular technology [Unified Theory of Acceptance and Use of Technology model]

Price value

Determinants of technology acceptance; Consumers' cognitive tradeoff between the perceived benefits of the applications and the monetary cost for using them [Unified Theory of Acceptance and Use of Technology model]

NORMALIZATION PROCESS COMPONENTS

CA: Relational integration

Target constructs (Logic model); Developing relationships with HCPs; Building confidence in the system and in relationships with HCPs [Normalization Process Theory]

BUSINESS MODELLING

Key partners

Key insights for building a minimum viable product; The key partners and their motivations to participate in the business model [Business Model Canvas]

Key activities

Key insights for building a minimum viable product; The key activities necessary to implement the business model [Business Model Canvas]

Key resources

Key insights for building a minimum viable product; The key resources needed to make the business model possible [Business Model Canvas]

Value propositions

Key insights for building a minimum viable product; The value proposition of what is offered to the market [Business Model Canvas]

Customer relationships

Key insights for building a minimum viable product; The relationships established with clients [Business Model Canvas]

Channels

Key insights for building a minimum viable product; The communication and distribution channels to reach clients and offer them the value proposition [Business Model Canvas]

Customer segments

Key insights for building a minimum viable product; The segment(s) of clients that are addressed by the value proposition [Business Model Canvas]

Cost structure

Key insights for building a minimum viable product; The cost structure resulting from the business model [Business Model Canvas]

Revenue streams

Key insights for building a minimum viable product; The revenue streams generated by the business model (constituting the revenue model) [Business Model Canvas]

Healt-related outcomes

Line-of-argument (Conceptual network)

OPERATIONALIZATION

Enablement

Intervention functions [Band et al. 2017; Behavior Change Wheel/COM-B model]
Intervention functions; Increasing means/reducing barriers to increase capability [Duff et al. 2018; Behavior Change Wheel]
Parameters of effectiveness; Intervention functions; Increasing means/reducing barriers to increase capability [Walsh et al. 2018a; Behavior Change Wheel/COM-B model]

Education (Intervention function)

Intervention functions [Band et al. 2017; Behavior Change Wheel/COM-B model]
Intervention functions; Increasing knowledge or understanding [Duff et al. 2018; Behavior Change Wheel]
Parameters of effectiveness; Intervention functions; Increasing knowledge or understanding [Walsh et al. 2018a; Behavior Change Wheel/COM-B model]

Environmental Restructuring

Intervention functions [Band et al. 2017; Behavior Change Wheel/COM-B model]
Intervention functions; Changing the physical or social context [Duff et al. 2018; Behavior Change Wheel]
Parameters of effectiveness; Intervention functions; Changing the physical or social context [Walsh et al. 2018a; Behavior Change Wheel/COM-B model]

Policy categories

Policy categories represent types of decisions made by authorities that help to support and enact an intervention [Behavior Change Wheel/COM-B model]

SOCIOTECHNICAL SYTEMS PERSPECTIVE

Work processes

Can be physical, cognitive, social/behavioral (performance) [Systems Engineering Initiative for Patient Safety 2.0]

Health-related work

Work processes; The exertion of effort and investment of time on the part of patients or family members to produce or accomplish something [Systems Engineering Initiative for Patient Safety 2.0]

Patient work

Work processes; Work performed by non-professionals without HCP involvement [Systems Engineering Initiative for Patient Safety 2.0]

Collaborative professional-patient work

Work processes; Work involving the active participation of both professionals and nonprofessionals [Systems Engineering Initiative for Patient Safety 2.0]

Outcomes

They are states or conditions resulting from the work process and can be proximal or distal, desirable or undesirable; they can refer to patient, professional, or organizational outcomes [Systems Engineering Initiative for Patient Safety 2.0]

Proximal/distal

Outcomes; Proximal and distal outcomes can be distinguished given that some outcomes may be the immediate result of work processes while others are further down the causal chain and may only emerge over time [Systems Engineering Initiative for Patient Safety 2.0]

Desirable / Undesirable

Outcomes; The outcomes might reflect the goals of different stakeholders such as clinicians, organizational leaders, regulators, payers and, perhaps most importantly, patients [Systems Engineering Initiative for Patient Safety 2.0]

Patient (outcomes)

Outcomes [Systems Engineering Initiative for Patient Safety 2.0]

CHALLENGES

Clinical inertia

The failure to establish appropriate targets and escalate treatment to achieve treatment goals [McGillicuddy et al. 2012]
Key outcome of HOME BP model [HOME BP]

Measuring changes in knowledge

Quantifying knowledge (e.g., about self-management) and ensuring that the change observed is neither random nor the result of measurement error is challenging, especially with a small sample size. This can be overcome, for example, via the TELER method [Bartlett et al. 2014]

ENGAGEMENT OUTCOMES

All patients are able to use the system and continue to use it for the duration of the evaluation

Outcomes (CMO hypotheses); This outcome can result from a combination of the eHealth literacy of the users (C) and the application of a user-centred design process (M) [CHF PSMS]

Continued engagement with system

Outcomes (CMO hypotheses); This outcome can result from the combination of an acceptable System Usability Scale score (C) and a lack of critical problems with the technology (M) [CHF PSMS]

BEHAVIORAL OUTCOMES

Mediators of behavior change

Outcomes of cardiac rehabilitation; Knowledge, goals, outcome expectations, perceived environmental impediments and facilitators) [PATHway]

Increased levels of knowledge about self-management.

Outcomes (CMO hypotheses); This outcome can result from the combination of the patient's current level of knowledge (C) and the provision of an information and advice section (M) [CHF PSMS]

HF knowledge

Health outcomes [HeartMapp]

Self-efficacy (outcome of cardiac rehabilitation)

Outcomes of cardiac rehabilitation [PATHway]

Improved self-care skills

Health outcomes [HeartMapp]

HF self-management

Health outcomes [HeartMapp]

Self-management tailored to patient's condition

Outcomes (CMO hypotheses); This outcome can result from the combination of the patient's co-morbidities (C) and the consideration of physical and psychological barriers to lifestyle changes (M) [CHF PSMS]

Behavior change that is sustainable over the long term

Outcomes (CMO hypotheses); This outcome can result from the combination of the patient's engagement with a variety of lifestyle changes (C) and the provision of behavioral change techniques such as self-monitoring of symptoms, goal setting according to the user's lifestyle, or feedback on activity performance (M) [CHF PSMS]

Patient lifestyle change

Patient intervention components [HOME BP]

Physical activity

Health outcomes [HeartMapp]

Ability to walk further, lose weight or meet personal goals

Outcomes (CMO hypotheses); This outcome can result from the combination of the patient's activation (C) and the provision of a walking intervention (e.g., via a mobile device) (M) [CHF PSMS]

Diet

Health outcomes [HeartMapp]

Balance between activity and rest

Outcomes (CMO hypotheses); This outcome can result from the combination of the over activity/ rest cycle of the patient (C) and the provision of feedback on activity (M) [CHF PSMS]

Patient medication adherence and titration

Patient intervention components [HOME BP]

Adherence to medication

Health outcomes [HeartMapp]

HEALTH OUTCOMES

Improved symptom control, ideally reducing need for health professional involvement.

Outcomes (CMO hypotheses); This outcome can result from the combination of the patient's condition stability (C) and the provision of self-monitoring and tailored feedback (M) [CHF PSMS]

Reduce readmissions

Health outcomes [HeartMapp]

Global health

Health outcomes [HeartMapp]

Quality of life

Health outcomes [HeartMapp]
